# Dipolar Coupling Errors in Nuclear Spin Qubits

**DOI:** 10.1002/advs.77080

**Published:** 2026-09-27

**Authors:** I. D. Thorvaldson, P. Macha, J. Reiner, S. H. Misha, Y. Chung, L. Kranz, D. Keith, S. Monir, D. Poulos, S. Sutherland, Y.‐L. Hsueh, B. Thorgrimsson, R. Rahman, J. G. Keizer, S. K. Gorman, M. Y. Simmons

**Affiliations:** ^1^ Silicon Quantum Computing Pty. Ltd. UNSW Sydney Australia; ^2^ Centre of Excellence for Quantum Computation and Communication Technology School of Physics UNSW Sydney NSW Australia

**Keywords:** anisotropic hyperfine, dipolar coupling, measurement, nuclear spins, phosphorus, quantum computing, silicon

## Abstract

Nuclear spins have been studied since the beginning of quantum information science since they form the prototypical qubit with long coherence times and high‐fidelity operation. To perform practical fault‐tolerant quantum computations, however, not only do we require precision manufacturing techniques to produce millions of qubits, but these techniques need to consistently yield low‐error qubits. A deep understanding and control of the interactions between the qubits and their environment are needed as we scale so that devices can be engineered to avoid high error rates. In this work, we experimentally and theoretically characterize two often‐overlooked error mechanisms affecting nuclear spin qubits in silicon caused by local interactions. The first is the direct magnetic interactions between nuclear spins, causing dipolar coupling errors, and the second is the interactions between the nuclear spins and their associated electron spin, causing “hotspots” of anisotropic hyperfine coupling errors. By modeling the properties of the combined electron–nuclear spin system as a function of magnetic field, contact hyperfine strength, and electron tunnel rate, we show how these errors can be reliably mitigated, giving rise to readout fidelities of ≳99.9%.

## Graphical abstract

Multi‐nuclear spin registers of phosphorus‐atom qubits in silicon have demonstrated 99.99% single and >99.9% two qubit gate fidelities. Despite nanometer separation, read‐out fidelities between individual nuclear spins within the register are not identical. Researchers show how previously unexplored dipolar coupling between nuclear spins and anisotropic hyperfine interactions can be mitigated to achieve read‐out fidelities of all nuclear spins >99.9%.

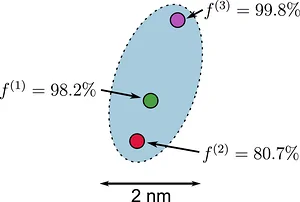

## 1 Introduction

To mitigate errors when performing quantum computation, error‐correcting codes are utilized to minimize errors, using redundancy to distribute logical quantum information over a large number of physical qubits [1, 2]. For this reduction in error to be practically realized, however, the error rate of the constituent physical qubits must be sufficiently small, typically ≪0.1% [3]. As such, it is important to understand which mechanisms limit physical qubit error rates so that they can be mitigated or otherwise engineered to improve qubit performance.

One long‐standing candidate for the realization of low‐error physical qubits are the spins of certain atomic nuclei [4, 5, 6, 7, 8, 9, 10, 11, 12, 13, 14]. In particular, by utilizing the nuclear spins of phosphorus atoms in silicon, Kane's proposal [15] married the remarkable coherence properties of nuclear spins [16] with silicon‐based nanofabrication technologies. Recent proposals [17, 18] have refined the Kane architecture to show how such systems can be realised in an all‐epitaxial system using Scanning Tunneling Microscope (STM) lithography [19, 20]. Precision lithography has also shown that closely spaced (≲2 nm) phosphorus atoms [21, 22, 23] can be used as multi‐nuclear spin registers, in which a single electron can interact with multiple nuclear qubits, addressable by their unique hyperfine couplings to the electron. Nuclear spin registers, first realized in 2007 [7], have been investigated in nitrogen‐vacancy (NV) centers [10, 11], as well as phosphorus atoms in silicon [13, 14, 24, 25, 26, 27]. Importantly, in these multi‐nuclear spin registers, a single electron can be used to address multiple nuclei, allowing for native multi‐qubit gates such as the multi‐controlled‐Z gate [10, 13, 14]. As a promising candidate for scalable quantum computation, it remains important to understand, and mitigate, any error sources in these multi‐nuclear spin registers from a materials perspective.

Nuclear spins can be measured with high accuracy by leveraging repeated quantum non‐demolition readout via the electron spin [8]. Readout fidelities above 99% for all nuclear spins within a single register have already been demonstrated in silicon [14]. In this paper, we seek to both experimentally and theoretically characterize which error sources contribute most to these measurements to further optimize nuclear readout fidelities. We perform control and readout of both electron and nuclear spins in a device with three closely spaced (≲2 nm) phosphorus atoms in isotopically purified 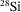 with a single bound electron. In previous work, nuclear spin errors during electron readout have been primarily attributed to “ionisation shock” arising from the isotropic hyperfine interaction between the electron and nuclear spins [8, 28], as well as a second‐order nuclear flip‐flop effect also caused by the isotropic hyperfine [29] (see Supporting Information V). However, in this work we identify two often‐overlooked mechanisms, which can limit nuclear readout fidelity, and characterize the strength of these mechanisms. First, we consider errors arising between pairs of nuclear spins via the direct through‐space nuclear–nuclear dipolar interaction, which can cause unwanted nuclear spin flip‐flops. Second, we consider errors from the anisotropic interaction of the electron and individual nuclear spins, which can, if not characterized and avoided, cause local “hotspots” in gatespace and magnetic field, with errors as large as 50%. We find strategies that can alleviate both sources of error, allowing devices to achieve readout fidelities above 99.9%, further improving on recent experimental results showing above 99.5% fidelity [30].

## 2 Readout of Triple‐Nuclear Spin Register

**FIGURE 1:**
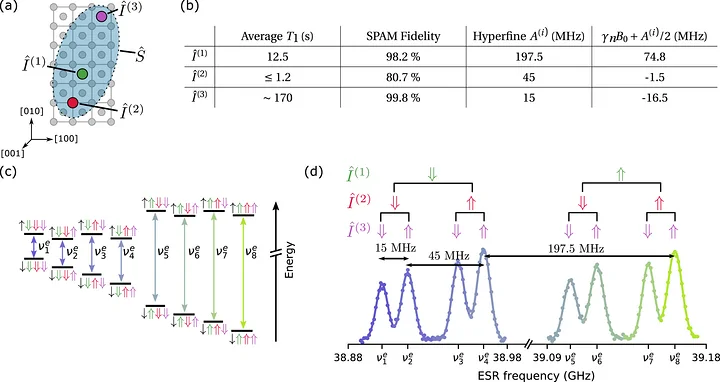
Four qubit nuclear spin register in silicon, with three nuclear and one electron spin qubit. (a) Locations of the three nuclear spin qubits studied here (${\hat{I}}^{\left(1\right)},{\hat{I}}^{\left(2\right)}$, and ${\hat{I}}^{\left(3\right)}$) in the 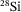 lattice, and the single bound electron ($\hat{S}$). The most likely locations of the phosphorus atoms within the silicon crystal can be identified using NEMO simulations to match the theoretical and observed hyperfine strengths (see Supporting Information I). (b) Summary of key parameters for the three nuclear spin qubits studied here. Note that the SPAM fidelity is described in Figure 2e–g with N=30 averages. (c) The energy level diagram of the four qubit nuclear spin register, illustrating the electron spin resonance (ESR) transitions (not to scale) for all eight nuclear spin configurations (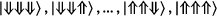). (d) ESR spectrum, with the corresponding nuclear spin states indicated above, indicating which transitions are used for readout of any particular nuclear spin configuration. Hyperfine coupling strengths of $A_{1}/2\pi =197.5\pm 1$ MHz, $A_{2}/2\pi =45\pm 1$ MHz, and $A_{3}/2\pi =15\pm 1$ MHz were extracted directly from the ESR spectrum.

In Figure 1a, we show the three phosphorus nuclear spins, ${\hat{I}}^{\left(1\right)},{\hat{I}}^{\left(2\right)}$, and ${\hat{I}}^{\left(3\right)}$, precisely placed in silicon using STM‐lithography [31] to form a multi‐nuclear spin register (see Experimental Section). The gray circles represent the surrounding silicon lattice, with the green, red, and purple circles showing the locations of the phosphorus atoms ${\hat{I}}^{\left(1\right)},{\hat{I}}^{\left(2\right)}$, and ${\hat{I}}^{\left(3\right)}$, respectively, and the blue oval representing the bound electron $\hat{S}$ shared between the atoms. By applying a static magnetic field of $B_{0}∼1.4$ T, parallel to the plane of the phosphorus atoms, electron, and nuclear spin states are split by the Zeeman interaction (∼2π·39 GHz and ∼2π·24 MHz, respectively). The standard Hamiltonian used to describe this system when the electron is present/loaded, ${\hat{H}}_{\mathrm{load}.}$, is [32]

(1) $$\begin{array}{cc} {\hat{H}}_{\mathrm{load}.}\left(B_{0}\right) & =\left(\gamma_{e}{\hat{S}}_{z}+\sum_{i=1}^{3}\gamma_{n}{\hat{I}}_{z}^{\left(i\right)}\right)B_{0}+\sum_{i=1}^{3}A^{\left(i\right)}\hat{S}\cdot {\hat{I}}^{\left(i\right)}\; \end{array}$$

where $\gamma_{e}/2\pi \approx 27.96$ GHz/T ($\gamma_{n}/2\pi \approx -17.24$ MHz/T) is the electron (phosphorus nuclear) gyromagnetic ratio, $\hat{S}$ (${\hat{I}}^{\left(i\right)}$) is the electron (ith phosphorus nuclear) spin operator, and $A^{\left(i\right)}$ is the contact hyperfine interaction between the electron and nuclear spin i. The hyperfines of the three nuclei, along with other measured parameters, are summarized in Figure 1b.

The energy levels given by Equation (1) are illustrated in Figure 1c, based on the hyperfine couplings observed on the device studied here (not to scale). Figure 1c is annotated with the eight electron spin resonance (ESR) transitions ($\nu_{i}^{e}$) that can be driven by applying an oscillating magnetic field $B_{1}$ perpendicular to $B_{0}$ at a frequency matching the respective energy splitting. Nuclear spins can also be driven using nuclear magnetic resonance (NMR) using the same broadband antenna used to control the electron spin [8]. Therefore, all spins (both electron and nuclear) of the register can be controlled using magnetic driving. From the splittings in the measured ESR spectrum (shown in Figure 1d), we extract the hyperfine coupling strengths of $A_{1}=197.5\pm 1$ MHz, $A_{2}=45\pm 1$ MHz, and $A_{3}=15\pm 1$ MHz. These experimental hyperfine strengths are compared to theoretical hyperfine values simulated for a variety of potential phosphorus atom locations using the 3D NanoElectronic MOdelling tool (NEMO‐3D) to establish the most likely locations of the phosphorus atoms [33, 34, 35, 36, 37, 38], as shown in Figure 1a (see Supporting Information I).

**FIGURE 2:**
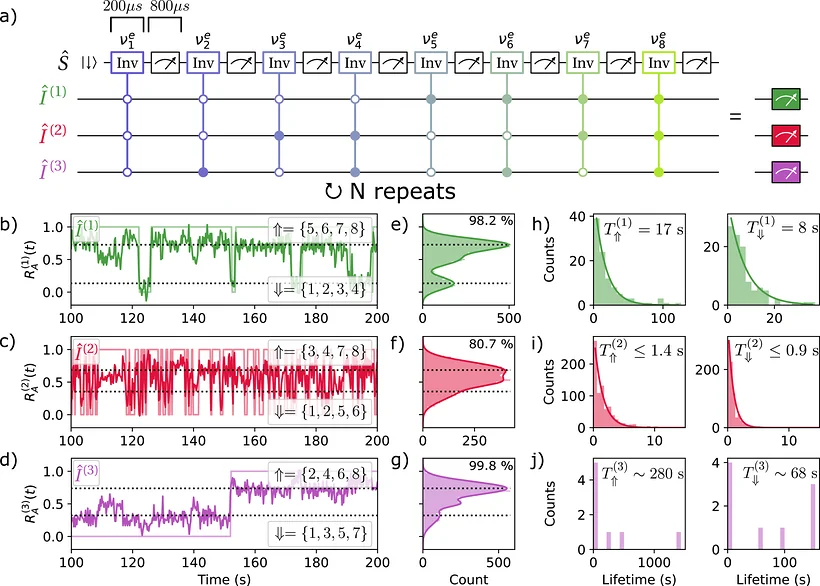
Non‐demolition measurement of the multi‐nuclear spin register to determine nuclear spin lifetimes. (a) Circuit implementing non‐demolition measurement of all three nuclear spin qubits via the electron spin. Each controlled inversion of the electron spin corresponds to a specific ESR frequency (shown above the inversion), inverted adiabatically (width=10 MHz, duration=200 μs). This circuit was repeated continuously over a 2880 s period to obtain statistics of nuclear spin flip behavior. (b–d) An example 100 s portion of the overall 2880 s trace, showing the states of nuclear spins ${\hat{I}}^{\left(1\right)},{\hat{I}}^{\left(2\right)}$, and ${\hat{I}}^{\left(3\right)}$, respectively, with N=30 averages. These traces show the raw signal (solid lines), upper and lower thresholds (dotted lines), and thresholded signal produced from double‐thresholding of the raw signal (faint solid lines), where the state only changes when the raw signal exceeds the opposite threshold. To obtain the raw signal, the circuit shown in (a) is performed N=30 times for a given time t, which provides probabilities of observing electron‐|↑⟩ over each ESR peak. These are then averaged together according to a given nuclear spin i being |⇑⟩ or |⇓⟩. Finally, the |⇑⟩ probability is averaged with the complement of the |⇓⟩ probability to produce $R_{A}^{\left(i\right)}\left(t\right)$ as plotted in the solid lines. The annotations note, which peaks correspond to spin‐⇑ and spin‐⇓ for that nuclear spin. (e–g) Histograms of the raw signal in (b–d) (faint), fitted to a triple‐Gaussian (solid line) to determine the thresholds (dotted lines) per Supporting Information III. (h–j) Histograms of the nuclear spin lifetimes of nuclear spins ${\hat{I}}^{\left(1\right)},{\hat{I}}^{\left(2\right)}$, and ${\hat{I}}^{\left(3\right)}$, respectively, for the |⇑⟩ (left) and |⇓⟩ (right) states, as well as exponential decay fits used to determine the $T_{1}$ time of nuclear spins ${\hat{I}}^{\left(1\right)}$ and ${\hat{I}}^{\left(2\right)}$. $T_{1}^{\left(3\right)}$ was estimated by taking the average of all observed lifetimes, giving values of $T_{⇑}∼280\pm 100$ s and $T_{⇓}∼68\pm 23$ s, respectively, with uncertainties $\propto 1/\sqrt{n}$, where n is the number of lifetimes observed.

Each ESR transition frequency ($\nu_{1}^{e}$ through $\nu_{8}^{e}$) corresponds to a unique nuclear configuration (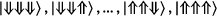), and hence can be used to flip the electron spin conditional on the nuclear spin configuration. Driving one of these transitions followed by electron spin readout, therefore, constitutes a quantum non‐demolition (QND) measurement of the nuclear spin configuration [8, 14, 32] (See Supporting Information II). By repeating this process for all eight ESR transitions and then repeating the entire process N times, we perform full nuclear spin readout of the multi‐nuclear spin register. This full process is illustrated in the circuit in Figure 2a. The nuclear spin configuration can then be determined by examining which ESR transition resulted in the electron spin being inverted.

Using this readout technique, we now look at the evolution of the three nuclear spins in time found by repeatedly applying the circuit in Figure 2a. For this particular processor, readout of all eight ESR transitions takes 8 ms, limited by the slow tunnel rate between the register and the SET of ∼200 μs, which in turn caused slow electron readout of 800 μs. Note that other phosphorus atom devices have been measured with significantly faster readout of 1 μs [39]. The readout process in Figure 2a is then repeated N = 30 times so that the full nuclear spin configuration is read out every 240 ms. To determine the state of a particular nuclear spin i within the register, we examine whether we are more likely to observe electron‐|↑⟩ on ESR transitions corresponding to i being |⇑⟩ as compared to ESR transitions where i is |⇓⟩. For example, as per the annotations above the peaks in Figure 1d, when measuring nuclear spin 1, we compare the probability of measuring electron‐|↑⟩ after driving peaks $\left\{\nu_{5}^{e},\nu_{6}^{e},\nu_{7}^{e},\nu_{8}^{e}\right\}$ (which would correspond to nuclear spin 1 being in the 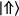 state) to the electron‐|↑⟩ after driving peaks $\left\{\nu_{1}^{e},\nu_{2}^{e},\nu_{3}^{e},\nu_{4}^{e}\right\}$ (nuclear spin 1 is 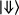). Quantitatively, we measure the following probabilities

(2) $$\begin{array}{cc} R_{⇑}^{\left(i\right)}\left(t\right) & \equiv P\left(\left|↑\right\rangle |{\hat{I}}_{z}^{\left(i\right)}\left(t\right)=⇑\right)\; \end{array}$$

(3) $$\begin{array}{cc} R_{⇓}^{\left(i\right)}\left(t\right) & \equiv P\left(\left|↑\right\rangle |{\hat{I}}_{z}^{\left(i\right)}\left(t\right)=⇓\right)\; \end{array}$$

which correspond to the probabilities of recording electron‐|↑⟩ after driving the peaks corresponding to ${\hat{I}}_{z}^{\left(i\right)}\left(t\right)=⇑$ (Equation 2) or ${\hat{I}}_{z}^{\left(i\right)}\left(t\right)=⇓$ (Equation 3). To construct a Random Telegraph Signal (RTS) of the nuclear spin state $R_{A}^{\left(i\right)}\left(t\right)$, we apply the following formula:

(4) $$\begin{array}{c} R_{A}^{\left(i\right)}\left(t\right)=\left[R_{⇑}^{\left(i\right)}\left(t\right)+1-R_{⇓}^{\left(i\right)}\left(t\right)\right]/2.\; \end{array}$$

By averaging $R_{⇑}^{\left(i\right)}\left(t\right)$ and $1-R_{⇓}^{\left(i\right)}\left(t\right)$ together in Equation (4), we improve the signal‐to‐noise for each spin (see Supporting Information II). The averaging is only possible because each nuclear spin has to be in one of two states (|⇑⟩ or |⇓⟩), which means that if an ${\hat{I}}_{z}^{\left(i\right)}\left(t\right)=⇑$ ESR peak is occupied, then the ${\hat{I}}_{z}^{\left(i\right)}\left(t\right)=⇓$ peaks will not be occupied, making $R_{⇑}^{\left(i\right)}\left(t\right)$ and $R_{⇓}^{\left(i\right)}\left(t\right)$ anti‐correlated. An example of the evolution of each individual spin (${\hat{I}}^{\left(1\right)},{\hat{I}}^{\left(2\right)}$, and ${\hat{I}}^{\left(3\right)}$) according to $R_{A}^{\left(i\right)}\left(t\right)$ is plotted in Figure 2b–d (solid lines).

The states of individual nuclei are discriminated according to a double threshold procedure (as shown by the histograms in Figure 2e–g), using the procedure outlined in Supporting Information III, where the spin will only be recorded as ⇑ if $R_{A}^{\left(i\right)}\left(t\right)$ exceeds an upper threshold, and as ⇓ only if $R_{A}^{\left(i\right)}\left(t\right)$ falls below a lower threshold, otherwise the recorded nuclear spin state will remain unchanged. For nuclear spins 1, 2 and 3, we obtain measurement fidelities of 98.2%, 80.7%, and 99.8%, respectively, as per Supporting Information IV. This is lower than nuclear readout fidelities for a single nuclear spin above 99.99% in previous work [8, 32], even with similar single‐shot electron readout speeds of 1 ms [8]. In this paper, we now investigate error channels that are specific to multi‐nuclear spin registers. We see that the measurement fidelity varies significantly between the three nuclear spins despite their nanometer‐scale proximity. We investigate the physical mechanisms, which give rise to the difference of fidelities between each nuclear spin in this multi‐nuclear spin register, and reconcile the difference in observed readout fidelity compared to previous work.

By recording statistics of the lifetimes for each nuclear spin state, the nuclear relaxation ($T_{⇓/⇑}$) times can be determined, as per the histograms in Figure 2h–j. Specifically, these measure the nuclear relaxation times under constant measurement (similar to [8]), which—as found later in this work—are primarily limited by the measurement process itself. The resulting $T_{⇓/⇑}$ times, annotated in Figure 2h–j, show a notable difference for each nuclear spin, with a few seconds for ${\hat{I}}^{\left(1\right)}$ and ≲1 s for ${\hat{I}}^{\left(2\right)}$, in contrast to timescales of minutes for nuclear spin ${\hat{I}}^{\left(3\right)}$. This latter value is much closer to the observed constant‐measurement $T_{⇓/⇑}$ times of ∼100 s for isolated single phosphorus atoms in silicon [8].

We provide a detailed summary of previous work examining the stability of the nuclear spins [40, 41, 42], quantified by their flip rates, in Supporting Information V. The primary error mechanisms previously studied (namely ionization shock [8] and multi‐spin ionization shock [29]) are both known to become stronger with increasing $A^{\left(i\right)}$, as both are caused by mixing between the nuclear and electron states. This is the opposite of what we observe here between nuclear spins ${\hat{I}}^{\left(1\right)}$ and ${\hat{I}}^{\left(2\right)}$. Specifically, both the lifetime (∼5 s) and readout fidelity (98.7%) of nuclear spin ${\hat{I}}^{\left(1\right)}$ are higher than that of nuclear spin ${\hat{I}}^{\left(2\right)}$ (≲1 s and 80.7%, respectively), despite ${\hat{I}}^{\left(1\right)}$ having a hyperfine strength of $A^{\left(1\right)}/2\pi =197.5$ MHz, larger than $A^{\left(2\right)}/2\pi =45$ MHz. Hence, a more careful consideration of nuclear spin errors is required to understand the nuclear spin lifetimes and measurement fidelities in this multi‐nuclear spin register (shown in Figure 2h–j). Additional error mechanisms (specifically, dipolar coupling and the anisotropic hyperfine interaction) also need to be included and have often been overlooked as error mechanisms for phosphorus atom qubits in silicon. By developing a quantitative understanding of these extra error mechanisms, we will show that fidelities above 99.9% can be reliably achieved by engineering electron tunnel rates, nuclear hyperfine coupling strengths, and the global magnetic field.

## 3 Direct Measurement of Nuclear–Nuclear Dipolar‐Coupling

To understand the factors limiting readout fidelities on the multi‐nuclear spin register studied here, we start by focusing on the readout of nuclear spin 1. Motivated by the fact that ${\hat{I}}^{\left(2\right)}$ has the highest error rate and that ${\hat{I}}^{\left(1\right)}$ is physically closer to ${\hat{I}}^{\left(2\right)}$ than ${\hat{I}}^{\left(3\right)}$ (as the hyperfine $A^{\left(2\right)}$ is stronger than $A^{\left(3\right)}$, per Figure 1a,b), we investigate interactions between ${\hat{I}}^{\left(1\right)}$ and ${\hat{I}}^{\left(2\right)}$, which could cause spin flips on ${\hat{I}}^{\left(2\right)}$ to be passed to ${\hat{I}}^{\left(1\right)}$. Because the device studied here has particularly slow tunnel rates of $T_{↓,in}∼200$ μs, we focus on interactions, which occur during readout. One such mechanism is the direct through‐space nuclear–nuclear dipolar coupling [43, 44, 45, 46]. In a strong magnetic field $B_{0}$, nuclear–nuclear dipolar coupling $b_{D}^{\left(i,j\right)}$ introduces an additional term $H_{D}$ to the Hamiltonian (see Supporting Information VI):

(5) $$\begin{array}{cc} H_{D} & =\sum_{i<j}b_{D}^{\left(i,j\right)}\left[3I_{z}^{\left(i\right)}I_{z}^{\left(j\right)}-{\hat{I}}^{\left(i\right)}\cdot {\hat{I}}^{\left(j\right)}\right].\; \end{array}$$

The dipolar coupling strength $b_{D}^{\left(i,j\right)}$ is defined by

(6) $$\begin{array}{c} b_{D}^{\left(i,j\right)}=\frac{1}{\hbar }\frac{\mu_{0}{\left(\hbar \gamma_{N}\right)}^{2}}{4\pi |r_{i,j}{|}^{3}}\left(\frac{3\cos^{2}\theta_{i,j}-1}{2}\right)\; \end{array}$$

where $\mu_{0}$ is the vacuum permeability, ℏ is the reduced Planck constant, $|r_{i,j}|$ is the length of the vector joining spins i and j, and $\theta_{i,j}$ is the angle between $r_{i,j}$ and $B_{0}$ (i.e., computational *z*‐axis). All terms are in SI units, with the exception of $b_{D}^{\left(i,j\right)}$ and $\gamma_{N}$, which are in units of ℏ=1 as per the rest of this paper. If we evaluate these constants for phosphorus nuclei in silicon, then in units of the silicon lattice constant (a=0.543095 nm), we get

(7) $$\begin{array}{c} \frac{1}{\hbar }\frac{\mu_{0}{\left(\hbar \gamma_{N}\right)}^{2}}{4\pi }=2\pi \cdot 122.886\mathrm{Hz}a^{3}\; \end{array}$$

Typically, dipolar coupling strengths are on the order of 100 Hz (from Equation 7). This means that when the electron is present, nuclear–nuclear dipolar coupling is effectively suppressed, since the hyperfine interaction energy scale is ∼100 MHz ≫100 Hz. Hence, when the electron is loaded on the register (as it is during typical coherent operation [14]), the nuclear–nuclear dipolar coupling does not significantly affect the nuclear spin dynamics (see Supporting Information VI). However, when the register is ionised, which occurs during the readout of an |↑⟩ electron, all nuclear spin splittings become approximately degenerate with each other, with a common splitting of $\gamma_{n}B_{0}$. This degeneracy has the result that the nuclear–nuclear dipolar coupling can now have a significant effect on the nuclear spin dynamics.

Specifically, the nuclear–nuclear dipolar interaction leads to pairs of dipolar‐coupled nuclear spins swapping with each other (i.e., |⇑⇓⟩↔|⇓⇑⟩). In the case that a pair of nuclei i,j start antiparallel, the probability that nuclei i and j will swap is given by (per Supporting Information VI)

(8) $$\begin{array}{c} P\left(\left|⇑⇓\right\rangle ↔\left|⇓⇑\right\rangle \right)=\frac{1}{2}-\frac{1}{2}\cos\left(b_{D}^{\left(i,j\right)}t\right).\; \end{array}$$

**FIGURE 3:**
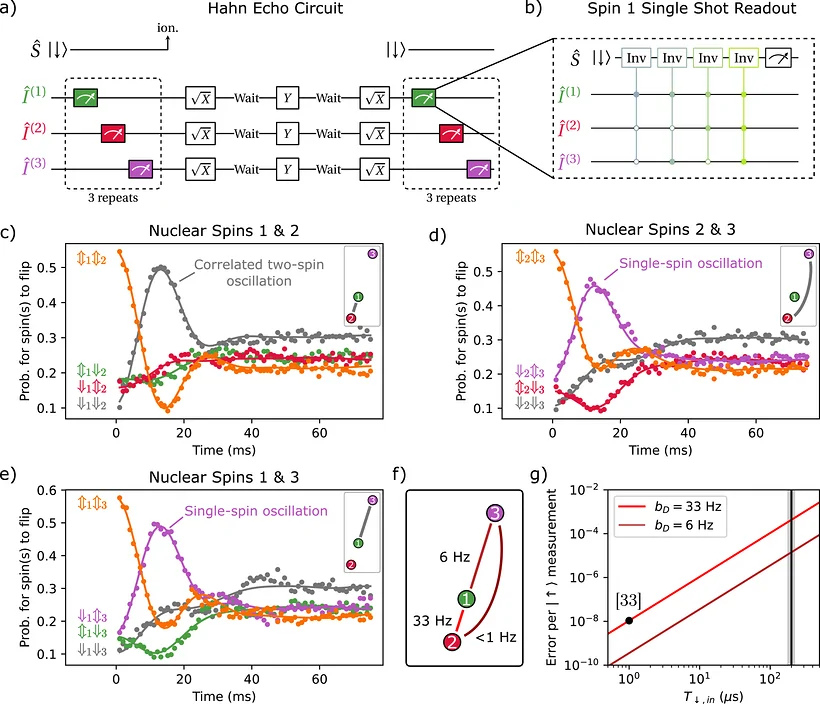
Direct measurement of the nuclear–nuclear dipolar coupling and correlated spin flip errors. (a) Hahn echo sequence used to measure nuclear–nuclear dipolar oscillations by refocusing $T_{2}^{*}$ noise. Note that the symbol “ion.” is introduced to depict ionization of the electron from the register, and |↓⟩ is later used to depict the loading of an initialized electron later in the circuit. For nuclear spin measurements, each shot is measured by initializing the electron, inverting it according to the ESR transitions corresponding to nuclear‐|⇑⟩ states as specified inset in Figure 2b–d, and then measuring the electron. (b) An example circuit illustrating a single‐shot measurement of nuclear spin 1. (c) Probabilities for nuclear spins 1 and/or 2 to flip, as a function of the total Hahn echo time. Specifically, we show the probabilities for both spins to flip (labeled ${⇕}_{1}{⇕}_{2}$), just spin 1 to flip (${⇕}_{1}{⇓}_{2}$), just spin 2 to flip (${⇓}_{1}{⇕}_{2}$), and for neither spin to flip (${⇓}_{1}{⇓}_{2}$). Note that the curve for ${⇓}_{1}{⇓}_{2}$ oscillates strongly, indicating correlated flips between nuclear spins 1 and 2. (d,e) Similar plots as compared to (c) but for nuclear spins 2 and 3 and 1 and 3 instead. Note that in (d) and (e), the single spin flip curves of ${⇓}_{2}{⇕}_{3}$ and ${⇓}_{1}{⇕}_{3}$, respectively, show significant oscillation, indicating no significant correlated oscillations between these pairs and hence that the strongest dipolar coupling is between nuclear spins 1 and 2. Data in (c–e) is postselected for readout of nuclear spin 2, only keeping measurements where all single‐shot measurements are in agreement. (f) The fitted dipolar coupling strengths $b_{D}^{\left(i,j\right)}/2\pi$ between each pair of nuclear spins obtained from (c–e). (g) Error incurred per |↑⟩ measurement due to the nuclear–nuclear dipole coupling, as a function of the |↓⟩‐in tunnel time, for each dipolar strength $b_{D}$ observed for this device. The tunnel rate for this device ($T_{↓,in}\approx 200$ μs) is indicated as a vertical black line, with the gray region, indicating the experimental uncertainty in the tunnel rate. The circular point indicates the impact of using a different device with a faster tunnel rate of $T_{↓,in}∼1$ μs, as reported by Keith et al. [39]

To experimentally measure this nuclear–nuclear dipolar coupling, a Hahn echo sequence was employed [47] (as illustrated in Figure 3a). Traditionally, a Hahn echo sequence is used to refocus slow‐varying noise in the frequency of a qubit [32]. However, when all nuclei are controlled simultaneously during ionised NMR control, the dipolar coupling Hamiltonian commutes with the refocusing pulse of the Hahn echo (as shown in Supporting Information VII). This commutation causes dipolar coupling between nuclear spins to produce oscillations in the Hahn echo experiment as a function of the delay time (as shown in Supporting Information VII), with more complex pulse sequences required to refocus the effects of dipolar coupling [48].

Since nuclear spin 2 was seen to have large error rates in Figure 2e–g, we used a different procedure to readout the nuclear spins compared to Figure 2. The aim was to reduce the number of operations used to measure the nuclei, so that the electron was removed from the register fewer times, in turn allowing fewer opportunities for errors to occur for spin 2. Instead of driving a single ESR frequency followed by an immediate electron measurement (Figure 2a), which results in low‐fidelity readout of nuclear spin 2, all 4 ESR frequencies corresponding to a particular nuclear spin i being |⇑⟩ were driven sequentially before measuring the electron. For example, nuclear spin ${\hat{I}}^{\left(1\right)}$ is measured by inverting the transitions $\nu_{5}^{e},\nu_{6}^{e},\nu_{7}^{e}$, and $\nu_{8}^{e}$ followed by an electron measurement, as illustrated in Figure 3b. A total of N=3 non‐demolition measurements of each nuclear spin was applied for each readout of the register, in the order shown at the start/end of Figure 3a. Such a readout scheme allows for each nucleus to be measured without a large number of ESR pulses and electron readouts, with 4·3·3=36 ESR pulses and 9 electron readouts used, as opposed to the 8·30=240 ESR pulses and electron readouts for Figure 2. This reduction in the number of ESR and readout pulses means that there is less chance for errors to occur, hence improving nuclear spin readout.

To further improve measurement fidelity, the readout of spin 2 was also postselected. Here we only keep data where either 0 or 3 of the 3 measurements performed on spin 2 registered electron |⇑⟩ (where 0 indicates ⇓ and 3 indicates ⇑), since these measurements have a lower probability of ${\hat{I}}^{\left(2\right)}$ flipping during readout (see Supporting Information VIII). Accounting for both sets of readouts in Figure 3a, this postselection procedure omits 68.4% of the original data taken.

Under the assumption that nuclear spins 1 and 2 have slightly different ionised NMR frequencies (as per Supporting Information IX, which uses methods from [49]), the resulting Hahn echo data was analyzed by binning pairs of before/after measurements surrounding the Hahn echo pulse (Figure 3a), to analyze which spin(s) flipped between the two measurements. For example, if the initial measurement registered 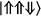 and the final measurement registered 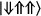, then this run would be binned along with a run initially measuring 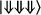 and finally measuring 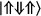. This is because in both cases spins 1 and 3 flip between the initial and final measurements, but spin 2 does not. The resulting correlations between the flipping of pairs of nuclei as a function of the total Hahn echo wait time can be seen in Figure 3c–e.

Importantly, Figure 3c shows a correlation between spins 1 and 2—either both nuclei flip (labeled 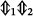), or neither flip (labeled 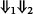), with the populations of these two cases oscillating out of phase, which indicates correlated oscillations between these nuclear spins. This correlation shows that there is a direct dipolar interaction between nuclei 1 and 2. In contrast, Figure 3d shows the flipping of spin 2 is mostly independent of spin 3 (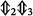 and 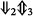 show anticorrelated oscillations), and similarly Figure 3e shows spin 1 oscillations are mostly independent of spin 3 (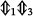 and 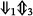 show anticorrelated oscillations). Combined, this behavior indicates substantially lower coupling between spins 1 and 3 and spins 2 and 3, respectively.

The dipolar oscillations in Figure 3c–e were fit to a numerical model (as described in Supporting Information X). The numerical fits are shown as the solid lines in Figure 3c–e, and provide an estimate for the strengths of the nuclear–nuclear dipolar couplings of $b_{D}^{\left(1,2\right)}/2\pi =32.8\pm 0.2$ Hz, $b_{D}^{\left(1,3\right)}/2\pi =6.0\pm 0.2$ Hz, $b_{D}^{\left(2,3\right)}/2\pi =0.0\pm 0.9$ Hz, as summarized in Figure 3f. According to Equation (6), a dipolar coupling of $b_{D}^{\left(1,2\right)}/2\pi =32.8\pm 0.2$ Hz between phosphorus nuclei implies a separation of $|r_{1,2}|\leq 1.554\pm 0.003\;a$, taking a maximum when $B_{0}$ is aligned with the inter‐nuclear vector. Simulations using NEMO‐3D can predict phosphorus atom locations using the measured hyperfine values [50, 51], as shown in Figure 1a, which gives a separation of $|r_{1,2}|=1.58\;a$ (as per Supporting Information I). The reason for the small (≳0.02a) discrepancy remains unknown, but is thought to originate from nearby charge defects, unexpected lattice strain, or leakage of the electron wavefunction, with further research needed for confirmation.

Knowledge of the dipolar coupling strength $b_{D}^{\left(1,2\right)}$ between nuclear spins 1 and 2 can now be used to predict the amount of error experienced by nuclear spin 1 during a readout event. During a readout event, if an |↑⟩ electron tunnels out, the dot will remain ionized for an exponentially distributed stochastic amount of time, with characteristic time $T_{↓,in}∼200$ μs. Using this distribution, and the dipolar oscillation frequency as per Equation (8), we can calculate the expectation value for nuclei 1 and 2 to flip‐flop during a readout ionization event

(9) $$\begin{array}{cc} & P\left(|{⇑}_{1}{⇓}_{2}\rangle ↔|{⇓}_{1}{⇑}_{2}\rangle \right)\\ & \;=\int_{0}^{T_{m}}\frac{1}{T_{↓,in}}e^{-t/T_{↓,in}}\left(\frac{1}{2}-\frac{1}{2}\cos\left(b_{D}^{\left(1,2\right)}t\right)\right)dt\\ & \;\approx \frac{{\left(b_{D}^{\left(1,2\right)}\right)}^{2}}{2}T_{↓,in}^{2}\; \end{array}$$

(10) $$\begin{array}{cc} & \approx 8.6\times 10^{-4}.\;\; \end{array}$$

Here, we assume that $b_{D}^{\left(1,2\right)}t\ll 1$ (i.e., the tunnel‐in time is sufficiently fast that we have much less than 1 flip‐flop oscillation), and $T_{↓,in}\ll T_{m}$ (i.e., the electron tunnel‐in time is much shorter than the full readout duration) for integration over the measurement time $T_{m}$. For the device studied here, $b_{D}^{\left(1,2\right)}T_{↓,in}\approx 0.0066$, $T_{↓,in}\approx 200\;\mu s$, and $T_{m}=0.8$ ms, justifying these assumptions. Under the assumption that each set of 8 readouts in Figure 2 had on average 1 electron‐|↑⟩ ionizsation event, assuming that only one nuclear configuration is populated, then the error rate in Equation (10) predicts that nuclear spins 1 and 2 should pick up an average error rate of $4.3\times 10^{-4}$ per ionization event, due to an assumed 50% chance for nuclei 1 and 2 to be in the states $|{⇑}_{1}{⇓}_{2}\rangle$ or $|{⇓}_{1}{⇑}_{2}\rangle$.

In Figure 2, we see that the average relaxation time for nuclear spin 1 $T_{⇓/⇑}^{\left(1\right)}$ is 12.5 s. We can use this to estimate the error rate per nuclear spin readout shot of this nuclear spin in this processor using $1-e^{-\tau_{R}/T_{⇓/⇑}}$ where $\tau_{R}=8$ ms is the time taken for a single shot of all 8 ESR peaks (see Supporting Information XI), which gives an error rate of $6.4\times 10^{-4}$ per ionization event. This is a close match to the observed error rate of $4.3\times 10^{-4}$ per ionization event calculated from the nuclear–nuclear dipolar coupling, explaining the observed lifetime of nuclear spin 1. Specifically, the presence of nuclear–nuclear dipolar coupling in multi‐nuclear spin registers causes nuclei to oscillate during periods of ionization (such as during readout), which explains the observed flip rate of nuclear spin 1 for the device studied here.

As is evident from Equation (9), the error rate due to nuclear–nuclear dipolar coupling is strongly dependent on the tunnel rates during readout. To be explicit, the electron‐|↓⟩ tunneling time onto the dot, $T_{↓,in}$, determines the duration of ionization. The calculated error rates expected as a function of $T_{↓,in}$ are plotted in Figure 3g for each of the three dipolar coupling strengths determined in this work. We now identify multiple pathways for mitigating these readout errors via atomic‐scale engineering. First, the multi‐nuclear spin registers can be engineered such that the separation between phosphorus atoms $r_{i,j}$ or the angle $\theta_{i,j}$ between phosphorus atom‐pairs minimizes $b_{D}^{\left(i,j\right)}$, and hence the error in Equation (9). Likewise, the angle of the magnetic field $B_{0}$ can be tuned to minimize $b_{D}^{\left(i,j\right)}$. However, the most straightforward avenue for avoiding the errors caused by dipolar coupling is to reduce the time spent ionized by shortening the electron tunnel time. Such tunnel times can be shortened by decreasing the distance between the phosphorus atoms and the reservoir, with shorter tunnel times being previously demonstrated as low as $T_{↓,in}∼1\;\mu s$ [21, 39]. Such short tunnel times would give negligible dipolar error rates ($∼10^{-8}$, as indicated by the circular black point on Figure 3g), and importantly is agnostic of the exact arrangement of nuclei within the register. For example, even if the dipolar coupling in a future device was 10× larger due to nuclei being separated by only 0.7 lattice constants (in‐plane next‐to‐nearest neighbor in silicon), the dipolar error rate would be suppressed to $∼10^{-6}$. Indeed, as plotted in Supporting Information V, reducing tunnel rates in this fashion would effectively remove dipolar coupling as a source of error, since other error sources are typically on the order of $10^{-6}-10^{-4}$. This trend further reinforces the requirement that electron tunnel rates need to be engineered sufficiently fast to facilitate high‐fidelity quantum operations.

The nuclear–nuclear dipolar interaction alone does not explain the flip rate observed for nuclear spin 2—if it did, we would expect nuclear spin 2 to have a similar flip rate to nuclear spin 1. To match the behavior of nuclear spin 2, we now need to also consider another overlooked mechanism, namely electron–nuclear anisotropic hyperfine coupling, which we quantify in the following section.

## 4 Electron–Nuclear Anisotropic Hyperfine Interaction

**FIGURE 4:**
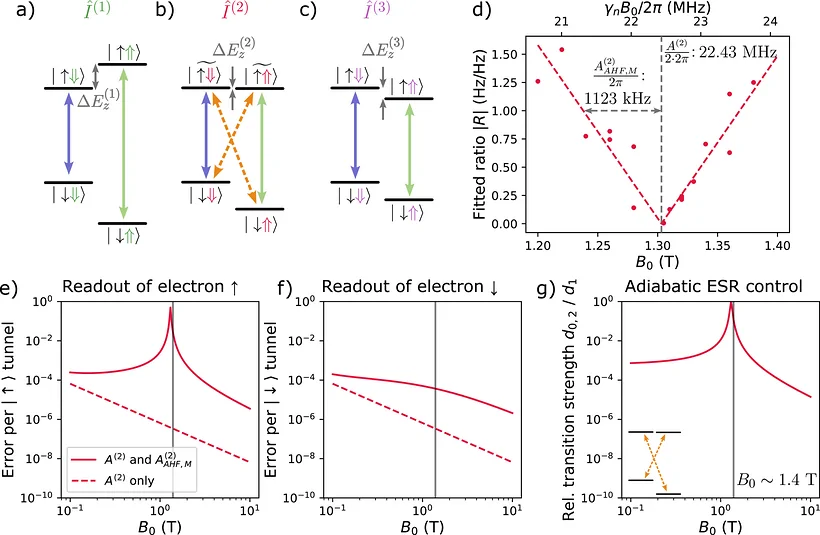
Impact of anisotropic hyperfine interactions on the nuclear spin qubit readout fidelity. (a–c) Energy levels in the subspace of the electron and nuclear spin 1, 2, and 3, respectively (not to scale). The ESR transitions (as per Figure 1) are shown (blue/lime), along with the $\Delta E_{z}^{\left(i\right)}$ splitting defined in main text Equation (11) (gray). Note that for nuclear spin 2 (b), we see near‐degenerate $|\tilde{↑⇓}\rangle$ and $|\tilde{↑⇑}\rangle$ states. Here, magnetic driving at ESR frequencies now gains access to two new transitions (orange dashed lines) where the nuclear spin flips along with the electron, giving a change of total spin of the register of 0 or 2. (d) Fitted ratio between $\Delta E_{z}^{\left(i\right)}$ and the anisotropic hyperfine (AHF) strength, $R\equiv \left|\Delta E_{z}^{\left(2\right)}/A_{AHF,M}^{\left(2\right)}\right|$, as a function of $B_{0}$ near the error hotspot. A linear fit (Equation 16) gives a contact hyperfine of $A^{\left(2\right)}/2\pi =44.87\pm 0.11$ MHz and an anisotropic hyperfine of $A_{AHF,M}^{\left(2\right)}/2\pi =1123\pm 70$ kHz, quantifying the amount of mixing caused by the AHF. (e,f) Theoretical errors induced per electron |↑⟩ or |↓⟩ tunnel event, including errors resulting from both contact ($A^{\left(2\right)}$) and anisotropic hyperfine ($A_{AHF}^{\left(2\right)}$) couplings, showing a narrow error hotspot for |↑⟩. The dashed lines indicate when we only consider errors due to the contact hyperfine interaction. (g) Theoretical errors due to the spin‐0/2 transitions during ESR driving, compared to the intended spin‐1 ESR transitions, expressed as a ratio of the transition dipole moments $d_{0/2}$ and $d_{1}$, also showing a narrow error hotspot. The gray line in (e–g) indicates the $B_{0}$ field used in the rest of this paper of 1.4 T. For simplicity, errors due to nuclear–nuclear dipolar coupling are ignored for this figure.

Having established the dominant error source for nuclear spin 1, we now consider possible error sources, which limit the lifetime of nuclear spin 2. Specifically, we look for a mechanism, which can cause errors to nuclear spin 2 but not nuclear spins 1 or 3. This rules out error mechanisms such as ionization shock [8, 29], as well as the dipolar coupling investigated in the previous section. To see what might affect only nuclear spin 2, in Figure 4a–c, we illustrate the energy levels in the subspace of the electron spin along with nuclear spins 1, 2, and 3, respectively. To first order, the energy splitting $\Delta E_{z}^{\left(i\right)}$ between |↑⇓⟩ and |↑⇑⟩ is

(11) $$\begin{array}{c} \Delta E_{z}^{\left(i\right)}\equiv \gamma_{n}B_{0}+A^{\left(i\right)}/2.\; \end{array}$$

Using Equation (11), we find the experimental values of $\Delta E_{z}^{\left(1\right)}/2\pi \approx 74.8\pm 0.5$ MHz, $\Delta E_{z}^{\left(2\right)}/2\pi \approx -1.5\pm 0.5$ MHz, and $\Delta E_{z}^{\left(3\right)}/2\pi \approx -16.5\pm 0.5$ MHz. Here, negative values of $\Delta E_{z}$ indicate that |↑⇓⟩ has a higher energy than |↑⇑⟩. From the measured values we see that nuclear spin 2 has a very small absolute energy splitting between |↑⇓⟩ and |↑⇑⟩, indicated by $\Delta E_{z}^{\left(2\right)}=-1.5$ MHz in Figure 4b. This is in contrast to spins 1 and 3, which have $\Delta E_{z}^{\left(1\right)}=74.8$ MHz and $\Delta E_{z}^{\left(3\right)}=-16.5$ MHz, respectively. This difference is due to the contact hyperfine strength of nuclear spin 2 ($A^{\left(2\right)}=45\pm 1$ MHz) in this particular processor being comparable in magnitude to twice the nuclear Zeeman splitting ($2\gamma_{n}B_{0}=-47.9$ MHz). That is, for nuclear spin 2, $A^{\left(2\right)}/2\approx \left|\gamma_{n}\right|B_{0}$, which is equivalent to $|\Delta E_{z}^{\left(2\right)}|\approx 0$. This causes the |↑⇓⟩ and |↑⇑⟩ states to be near‐degenerate, meaning that any weak interactions between |↑⇓⟩ and |↑⇑⟩ will now become relevant and lift the degeneracy. One such interaction is the anisotropic hyperfine (AHF) interaction, which modifies the Hamiltonian in Equation (1) as follows:

(12) $$\begin{array}{cc} {\hat{H}}_{\mathrm{load}.}^{\prime }\left(B_{0}\right) & ={\hat{H}}_{\mathrm{load}.}\left(B_{0}\right)+\sum_{i=1}^{3}\hat{S}\cdot \overset{↔}{A_{AHF}^{\left(i\right)}}\cdot {\hat{I}}^{\left(i\right)}\; \end{array}$$

where ${\hat{H}}_{\mathrm{load}.}\left(B_{0}\right)$ is the Hamiltonian from Equation (1). The above expression additionally considers the contribution of the anisotropic hyperfine tensor $\overset{↔}{A_{B}^{\left(i\right)}}$ which arises from the magnetic dipolar interaction between the electron and the nuclear spins [25]. This tensor not only introduces terms proportional to ${\hat{S}}_{x}{\hat{I}}_{x}$, ${\hat{S}}_{y}{\hat{I}}_{y}$, and ${\hat{S}}_{z}{\hat{I}}_{z}$, similar to the contact hyperfine, but it also introduces numerous additional terms proportional to ${\hat{S}}_{x}{\hat{I}}_{y}$, ${\hat{S}}_{x}{\hat{I}}_{z}$, ${\hat{S}}_{y}{\hat{I}}_{x}$, and so on. The effect of the anisotropic hyperfine interaction has been typically ignored in silicon systems since it is predicted to be small (∼100 kHz) compared to the contact hyperfine A which is typically ∼100 MHz (around 2–3 orders of magnitude larger) [25]. However, since the anisotropic hyperfine tensor contains z,x and z,y terms, which are off‐diagonal in the {|↑⇓⟩,|↑⇑⟩} basis, when $\Delta E_{z}∼0$ (as is the case for nuclear spin 2), there can be significant mixing between |↑⇓⟩ and |↑⇑⟩ [28, 52]. Indeed, this mixing becomes maximized when $\Delta E_{z}=0$. We can characterize the strength of this off‐diagonal nuclear mixing term ${\left[A_{AHF}^{\left(i\right)}\right]}_{z,x}\pm i{\left[A_{AHF}^{\left(i\right)}\right]}_{z,y}$ in the AHF tensor by its magnitude

(13) $$\begin{array}{c} A_{AHF,M}^{\left(i\right)}\equiv \sqrt{{\left[A_{AHF}^{\left(i\right)}\right]}_{z,x}^{2}+{\left[A_{AHF}^{\left(i\right)}\right]}_{z,y}^{2}} \end{array}$$

which produces mixing in the |↑⇑⟩ and |↑⇓⟩ subspace (per Supporting Information XII)

(14) $$\begin{array}{cc} {\hat{H}}_{\mathrm{load}.}^{\prime }\left(B_{0}\right) & \approx \left[\begin{array}{cc} \frac{\Delta E_{z}^{\left(i\right)}}{2} & \frac{A_{AHF,M}^{\left(i\right)}}{4}\\ \frac{A_{AHF,M}^{\left(i\right)}}{4} & -\frac{\Delta E_{z}^{\left(i\right)}}{2} \end{array}\right]. \end{array}$$

To understand what impact the AHF interaction has on readout fidelity when $\Delta E_{z}∼0$, we first note that the chirped ESR inversion pulse used for nuclear spin readout has a chance to not only flip the electron spin but also flip the nuclear spin. This is illustrated by the orange dashed lines in Figure 4b, and arises due to mixing between $|\tilde{↑⇓}\rangle$ and $|\tilde{↑⇑}\rangle$. This means that ESR can change the total spin of the register by either 0 or 2 (i.e., $|\tilde{↑⇓}\rangle ↔\left|↓⇑\right\rangle$ or $\left|↓⇓\right\rangle ↔|\tilde{↑⇑}\rangle$). This is different compared to usual ESR transitions, which only flip the electron and hence change the total spin of the register by 1 (i.e., |↑⇓⟩↔|↓⇓⟩ or |↓⇑⟩↔|↑⇑⟩). We refer to these unusual ESR transitions as spin‐0/2 errors. As $\Delta E_{z}\to 0$, these spin‐0/2 errors approach the ESR inversion rate, which can be arbitrarily close to 100% (see Supporting Information XIII, using analysis techniques from [53]). We also note that the introduction of these transitions may cause limited loss of fidelity during ESR gates due to frequency crosstalk, but these errors are expected to be less significant than the SPAM errors considered here due to the improved frequency addressability for coherent single‐tone drives. A thorough analysis of the impact on coherent control is beyond the scope of this work.

Second, this mixing between $|\tilde{↑⇓}\rangle$ and $|\tilde{↑⇑}\rangle$ can cause the ionization shock to have an increased impact on nuclear spin readout, as derived in Supporting Information XII. The mixing between nuclear |⇓⟩ and |⇑⟩ due to the AHF can become suddenly activated (deactivated) if an electron‐|↑⟩ tunnels onto (off) the dot, causing error as mixed states are projected onto non‐mixed states or visa versa. The AHF has a minimal effect for electron‐|↓⟩ tunnel events, since |↓⇓⟩ and |↓⇑⟩ are not close in energy, but the AHF will still cause a small amount of mixing for |↓⟩. As $\Delta E_{z}\to 0$, the ionization shock error (during electron‐|↑⟩ tunnel events) is now dominated by the AHF, and approaches 50% due to maximal mixing between $|\tilde{↑⇓}\rangle$ and $|\tilde{↑⇑}\rangle$.

For both of these mechanisms, it is important to note that mixing caused by the AHF interaction is only significant when $\Delta E_{z}$ is at a similar magnitude to the AHF mixing term $A_{AHF,M}$. Due to the small magnitude of $A_{AHF,M}$, both of the above effects only occur over very small ranges or “hotspots” as a function $B_{0}$ and $A^{\left(i\right)}$. To quantify this effect, we introduce the ratio R:

(15) $$\begin{array}{c} R\equiv \frac{\Delta E_{z}^{\left(i\right)}}{A_{AHF,M}^{\left(i\right)}} \end{array}$$

which must be small for AHF errors to be relevant. Estimates for $\Delta E_{z}^{\left(2\right)}\equiv \gamma_{n}B_{0}+A^{\left(2\right)}/2$ can be made from the applied magnetic field $B_{0}=1.39$ T (or directly measuring $\gamma_{n}B_{0}$ using ionized NMR) and the measured value of $A^{\left(2\right)}/2\pi =45\pm 1$ MHz from Figure 1c, meaning that the AHF hotspot location can be readily calculated after standard characterization of the device. However, the anisotropic hyperfine mixing strength $A_{AHF,M}^{\left(2\right)}$ is not directly measurable, meaning that the width of the hotspot is less straightforward to determine. Instead, we determine the anisotropic hyperfine strength indirectly through the nuclear spin error rate. Specifically, we perform non‐demolition readout of both nuclear spin 1 and 2 using N=49 repetitions. We do this for a series of different $B_{0}$ strengths, ensuring that the ESR frequency chirps are performed in increasing frequency for $\Delta E_{z}^{\left(2\right)}>0$ and decreasing frequency for $\Delta E_{z}^{\left(2\right)}<0$ to avoid any errors that could be caused by EDSR [27]. By examining histograms similar to Figure 2e–g for the first n readouts (n∈[1,N]) as a function of n, we can determine how nuclear spin errors accumulate as more ESR and electron readouts are applied. This reshaping allows us to fit the ratio |R| at each $B_{0}$ field (see Supporting Information XIV), which are plotted in Figure 4d. A linear fit for |R| according to

(16) $$\begin{array}{cc} \left|R(B_{0})\right| & =\left|\frac{\gamma_{n}B_{0}+\frac{A^{\left(2\right)}}{2}}{A_{AHF,M}^{\left(i\right)}}\right|\; \end{array}$$

gives us the value of the contact hyperfine directly as $A^{\left(2\right)}/2\pi =44.87\pm 0.11$ MHz, and, more importantly, we can now extract a value for the anisotropic hyperfine of $A_{AHF,M}^{\left(2\right)}/2\pi =1123\pm 70$ kHz. This value is somewhat higher than the value expected from past work of $A_{AHF,M}^{\left(2\right)}/2\pi ∼100-200$ kHz [25, 54]. The AHF strength is dependent on the anisotropy of the electron wavefunction, the discrepancy between theoretical and observed values of $A_{AHF,M}^{\left(2\right)}$ suggests that the electron wavefunction is very anisotropic in the device studied. A potential source for this could include the anisotropic electric field environment present in nanoelectronic devices. Another potential cause could be secondary electron tunneling events causing increased error rates.

Using the observed value of the anisotropic hyperfine found in Figure 4d, we now predict the effect of AHF errors over a wide range of $B_{0}$ values. The predicted errors induced by ionization shock (due to the presence of both contact and anisotropic hyperfine interactions) on nuclear spin 2 are shown in Figure 4e,f. Figure 4e shows narrow error regions as high as 50% for electron‐|↑⟩ tunnel events. It is important to note that such high errors only occur when the tunnel event involves an |↑⟩ electron, since it is these states that are nearly degenerate (see Figure 4b). This error hotspot is not observed for |↓⟩‐electron tunnel events in Figure 4f.

If we examine the hotspot in Figure 4e where significant anisotropic hyperfine induced ionisation shock errors occur, we observe that it is very narrow. Specifically, we see that readout errors are only above 1% over a range of ∼300 mT in $B_{0}$, or equivalently a range of ∼10 MHz in $A^{\left(2\right)}/2\pi$ (see Supporting Information XV). This implies errors are only above 1 % when $|\Delta E_{z}|≲2.5A_{AHF,M}^{\left(i\right)}$, or equivalently |R|≲2.5. This then quantifies the width of the AHF error hotspot at 1 % error. Similarly, errors are above 0.1% error during electron readout when |R|≲7.5. Experimentally, this gives a quantitative bound on how large |R| must be made in order to achieve low nuclear spin errors during electron readout. The contact hyperfine mediated ionization shock is even lower ($∼10^{-8}-10^{-4}$ per ionization event [8]) meaning that it is the anisotropic hyperfine errors which dominate over a wide range of $B_{0}$ values.

The error rate for spin‐0/2 transitions compared to the intended spin‐1 transitions for nuclear spin 2 are shown in Figure 4g, as a function of $B_{0}$, again showing a narrow region of very high error 1. Since spin‐0/2 errors require the ESR chirp to pass over their transition frequencies, if ESR chirp widths are kept to within 10 MHz, these errors will not extend further than ∼580 mT from a given hotspot. They are plotted over a large range, however, to show their general trend in Figure 4g.

As is plotted in Supporting Information V, the AHF‐mediated ionization shock can become the dominant error—especially, if dipolar coupling errors have already been mitigated by moving to faster tunnel rates. To avoid such errors, |R| should be maximized to operate as far as possible from the hotspot, which can be achieved in three ways. First, for devices that only contain a few nuclear spins, one can model the hotspot locations and choose a magnetic field where nuclear spin error is minimized (e.g., extending the experimental demonstration in Figure 4d by setting $B_{0}≳$ 2 T or $B_{0}≲$ 1 T according to Figure 4e–g). However, if more nuclear spins are present, it may not be possible to find a magnetic field value, which avoids all hotspots to an acceptable level. Instead, as devices scale up, it is better to fix the $B_{0}$ field, and instead tune the contact hyperfine strengths so that the error hotspots are far from the desired operating $B_{0}$ field strength. This can be achieved by engineering donor positions, provided that one can achieve sufficiently accurate placement of phosphorus atoms using STM‐hydrogen lithography [31, 55, 56, 57, 58]. If such accurate placement cannot be achieved, hyperfine strengths could still be engineered to avoid hotspots—for example, using the fact that a 2P register will have average hyperfine ≳50 MHz [25], and setting the magnetic field such that $2\gamma_{n}B_{0}\ll 50$ MHz (i.e., $B_{0}\ll 1.46$ T). Finally, the Stark shift can be used to further tune contact hyperfine strengths [59]. Using these methods to ensure $\gamma_{n}B_{0}+A^{\left(2\right)}/2≳2.5A_{AHF,M}^{\left(i\right)}∼2\pi \cdot 2.8$ MHz, nuclear spin errors from the anisotropic hyperfine can be reduced below 1% as per Supporting Information XV. Alternatively, if $\gamma_{n}B_{0}+A^{\left(2\right)}/2≳7.5A_{AHF,M}^{\left(i\right)}∼2\pi \cdot 8.4$ MHz, errors drop below 0.1%.

## 5 Summary and Discussion

The scaling of quantum hardware requires a deep understanding of the error sources affecting the qubit platform to ensure error rates can be reliably kept low over millions of qubits. In this paper, we consider how nuclear readout fidelities can be optimized in nanometre‐scale nuclear spin registers by characterizing material‐specific properties. First, we considered magnetic dipolar interactions between each pair of phosphorus nuclear spins, where we observe a dipolar interaction between nuclear spins 1 and 2 of $b_{D}^{\left(1,2\right)}/2\pi =33$ Hz. Second, anisotropic hyperfine interactions between the phosphorus nuclear spin and the bound single electron spin were considered, where we observed mixing of nuclear spin 2 due to the anisotropic hyperfine with a strength of $A_{AHF,M}^{\left(2\right)}=1123\pm 70$ kHz. Importantly, we found that to reduce both these dipolar coupling errors below 0.1 % error, the following is required:

- To reduce errors from nuclear–nuclear dipolar coupling, one can reduce the distance between the SET reservoir and the register to reduce the tunnel time $T_{↓,in}$ for an electron‐|↓⟩ to tunnel onto the dot, which can be readily achieved using atomically precise fabrication using STM hydrogen‐resist lithography [31, 55]. Alternatively, one can minimize the strength of dipolar coupling, again through atomic‐precision manufacturing techniques to increase the inter‐nuclear distance $|r_{i,j}|$, and/or through reorientation of the global magnetic field $\theta_{i,j}$. These techniques work because, in order to achieve dipolar‐coupling errors below 0.1%, the time for an electron‐|↓⟩ to tunnel onto the dot ($T_{↓,in}$) must be reduced below $1/\sqrt{0.001}=32$ times smaller than the reciprocal of the dipolar coupling strength $b_{D}^{\left(i,j\right)}$; that is, $T_{↓,in}<1/\left(\sqrt{0.001}\;b_{D}^{\left(i,j\right)}\right)$.
- To reduce errors from the anisotropic hyperfine coupling, one can engineer the strength of the contact hyperfine $A^{\left(i\right)}$ can be engineered using atomic‐precision manufacturing techniques to achieve target hyperfine strengths, and then fine‐tuned with electric fields by utilizing the Stark shift. Alternatively, the strength of the global magnetic field $B_{0}$ can be tuned to produce a target ionized nuclear energy splitting. Finally, the arrangement of the phosphorus atoms and direction of the $B_{0}$ magnetic field can be atomistically engineered to minimize the coupling term $A_{AHF,M}^{\left(2\right)}$. These techniques work because, to achieve AHF errors below 0.1%, the frequency of the contact hyperfine $A^{\left(i\right)}$ needs to be further than $16A_{AHF,M}^{\left(i\right)}$ away from twice the absolute ionized nuclear energy splitting $|\gamma_{n}B_{0}|$; that is, $|2\gamma_{n}B_{0}+A^{\left(i\right)}|<15A_{AHF,M}^{\left(i\right)}$. For the device studied here, the hyperfine of nuclear spin 2 would need to be changed by $15A_{AHF,M}^{\left(2\right)}\approx 2\pi \cdot 16.8$ MHz, or alternatively the magnetic field would need to be changed by $7.5A_{AHF,M}^{\left(2\right)}/\gamma_{n}\approx 500$ mT to achieve these low error rates.

Because the tunnel rate, magnetic field and contact hyperfine strength are engineered with different physical parameters (dot‐reservoir distance, applied field strength and donor arrangement respectively), both error mechanisms studied in this work can be optimised simultaneously. As discussed in detail in Supporting Information V, other error sources affecting nuclear spin flips can also be optimized to below $10^{-3}$. As such, the engineering strategies outlined in this work can be utilized to achieve fidelities in excess of 99.9%. With future experimental confirmation of these error mitigation strategies, the SPAM performance of multi‐nuclear spin register devices will be significantly improved for both near‐term and long‐term applications.

## 6 Conclusion

Understanding and characterizing dipolar‐induced errors is important as we scale spin qubit architectures. As both dipolar and anisotropic hyperfine mechanisms stem from stochastic tunneling events, alternate readout schemes where the electron is not stochastically removed from the register, such as dispersive readout [60], can intrinsically avoid both effects. Even with stochastic‐tunneling‐based readout, nuclear–nuclear dipolar coupling errors can be avoided by decreasing the tunnel time $T_{↓,in}$, which can achieved through atomic‐scale engineering of the distance between the register and reservoir [39]. Anisotropic hyperfine errors can be minimized in a variety of ways, either through engineering of the phosphorus atom locations or using the Stark shift to control nuclear hyperfine couplings, or by tuning of the global magnetic field $B_{0}$ to avoid error hot spots. Through these techniques, we have shown that engineering of the tunnel rate, hyperfines and magnetic field will reliably produce nuclear SPAM errors below 0.1%, further improving recent experimental results, which show SPAM fidelities above 99.5% [30].

## 7 Experimental Section

### 7.1 Manufacture of the Multi‐Nuclear Spin Register

The device studied here was manufactured using Scanning Tunneling Microscope (STM) hydrogen lithography adapted to include epitaxial growth of isotopically pure silicon (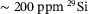) to suppress the nuclear spin noise and increase the spin coherence times. The epitaxial growth includes two separate steps: a high‐temperature growth of ∼ 40 nm purified buffer layer that decouples the device from the spin bath of the natural silicon substrate, followed by a low‐temperature growth of ∼ 40 nm purified encapsulation layer, carried out after the STM lithography and phosphorus (P) incorporation steps. The encapsulation layer growth parameters (∼ 250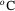, 0.14 nm/min) were optimized to ensure high‐quality epitaxy, reducing the charge noise around the qubits, while limiting the thermal budget to avoid phosphorus atom segregation and diffusion that would destroy the atomically defined structures.

**FIGURE 5:**
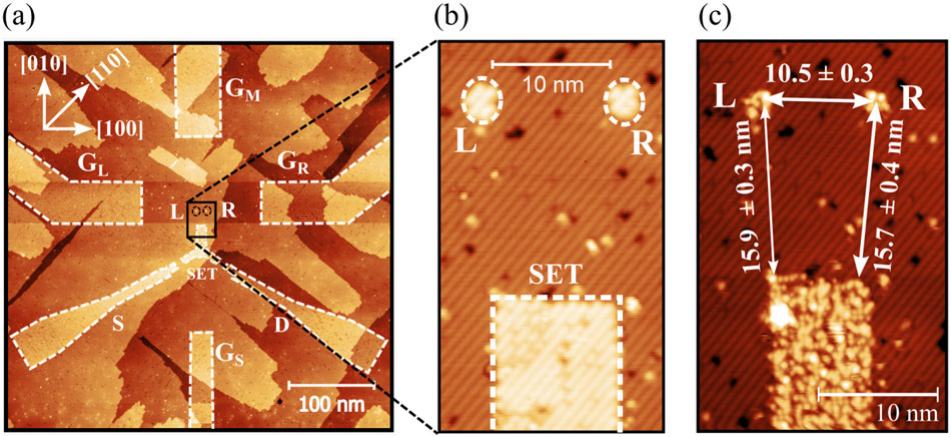
Scanning probe microscope images of the device containing the left dot multi‐nuclear spin register with three phosphorus nuclei. (a) Outline of the device hydrogen resist lithography. The device comprises of two registers (only the left register L is considered in this work, which contains three phosphorus atoms), an SET charge sensor coupled to source (S) and drain (D) leads, and four control gates ($G_{S}$, $G_{L}$, $G_{R}$, and $G_{M}$). (b) Zoomed‐in topography of the edge of the SET island and left (L) and right (R) registers after hydrogen resist desorption. (c) Same area as in (b) but after phosphine dosing. The ${\mathrm{PH}}_{x}$ molecules attach to reactive Si bonds where the resist is removed, allowing for phosphorus incorporation into the silicon lattice, with 3P in the left register and 2P on the right register.

An image of the device, taken with the STM, can be seen in Figure 5a. The device consists of four gates used to control the electrochemical potential of two multi‐nuclear spin registers and a large quantum dot used as a single‐electron tramsistor (SET) for readout shown in Figure 5b. The bright regions correspond to where the atomic hydrogen layer has been desorbed using the STM tip to define the active regions of the device. After dosing the system with phosphine gas (${\mathrm{PH}}_{3}$) partial adsorption (${\mathrm{PH}}_{x<3}$ species) can be seen in Figure 5c. Additionally, the SET acts as an electron reservoir with the multi‐nuclear spin registers weakly tunnel coupled to the end of the SET quantum dot. The device consists of two individual multi‐nuclear spin registers denoted by L and R in Figure 5. In the main text, we focus on the L multi‐nuclear spin register since the R register was strongly coupled to a fluctuator(s) making the ESR spectrum not reproducible, and hence we could not perform high‐fidelity nuclear spin readout.

## Author Contributions

P.M., J.R., L.K., S.K.G., and M.Y.S. conceived the project. J.R., S.H.M., Y.C., and J.G.K. fabricated the device. I.D.T., P.M., J.R., D.K., D.P., and B.T. performed the measurements. I.D.T., S.M., S.S., Y.L.H., and R.R. performed the theoretical calculations. The manuscript was written by I.D.T., P.M., J.R., S.M., S.K.G., and M.Y.S. with input from all authors. M.Y.S. supervised the project.

## Conflicts of Interest

M.Y.S. is a director of the company Silicon Quantum Computing Pty. Ltd. P.M., J.R., S.H.M., Y.C., L.K., D.K., S.M., D.P., Y.‐L.H., B.T., R.R., J.G.K., S.K.G., and M.Y.S. declare equity interest in Silicon Quantum Computing Pty. Ltd. I.D.T. and S.S. are staff members of Silicon Quantum Computing Pty. Ltd. without equity interest.

## Supporting information


**Supporting FIle**: advs77080‐sup‐0001‐SuppMat.pdf.

## Data Availability

The data and analysis code that support the findings of this study are available at: http://doi.org/10.5281/zenodo.22073204.

## Endnotes

1 The probability of an error occurring due to a spin‐0/2 transition depends on the inversion rate of the intended spin‐1 transitions. The exact expression for this relationship can be found in Supporting Information VIII, but can be arbitrarily close to 100%.
