# Supplementary material for: Dipolar Coupling Errors in Nuclear Spin Qubits
Source: Adv Sci (Weinh). 2026 Sep 27:e77080. Online ahead of print. doi: 10.1002/advs.77080 (PMC13616344; doi:10.1002/advs.77080)
Supplement: Supplementary file 1 [file ADVS-9999-e77080-s001.pdf]

Supporting Information: Dipolar Coupling Errors in Nuclear Spin Qubits

I. D. Thorvaldson,* P. Macha,* J. Reiner,* S. H. Misha, Y. Chung, L. Kranz, D. Keith, S. Monir, D. Poulos, S. Sutherland, Y.-L. Hsueh, B. Thorgrimsson, R. Rahman, J. G. Keizer, S. K. Gorman, and M. Y. Simmons
Corresponding author: michelle.simmons@sqc.com.au
Silicon Quantum Computing Pty. Ltd., UNSW Sydney, 2052, Australia and
Centre of Excellence for Quantum Computation and Communication Technology,
School of Physics, UNSW Sydney, 2052, NSW Australia
(Dated: September 2, 2026)

I. MODELLING OF PHOSPHORUS ATOM LOCATIONS USING NEMO

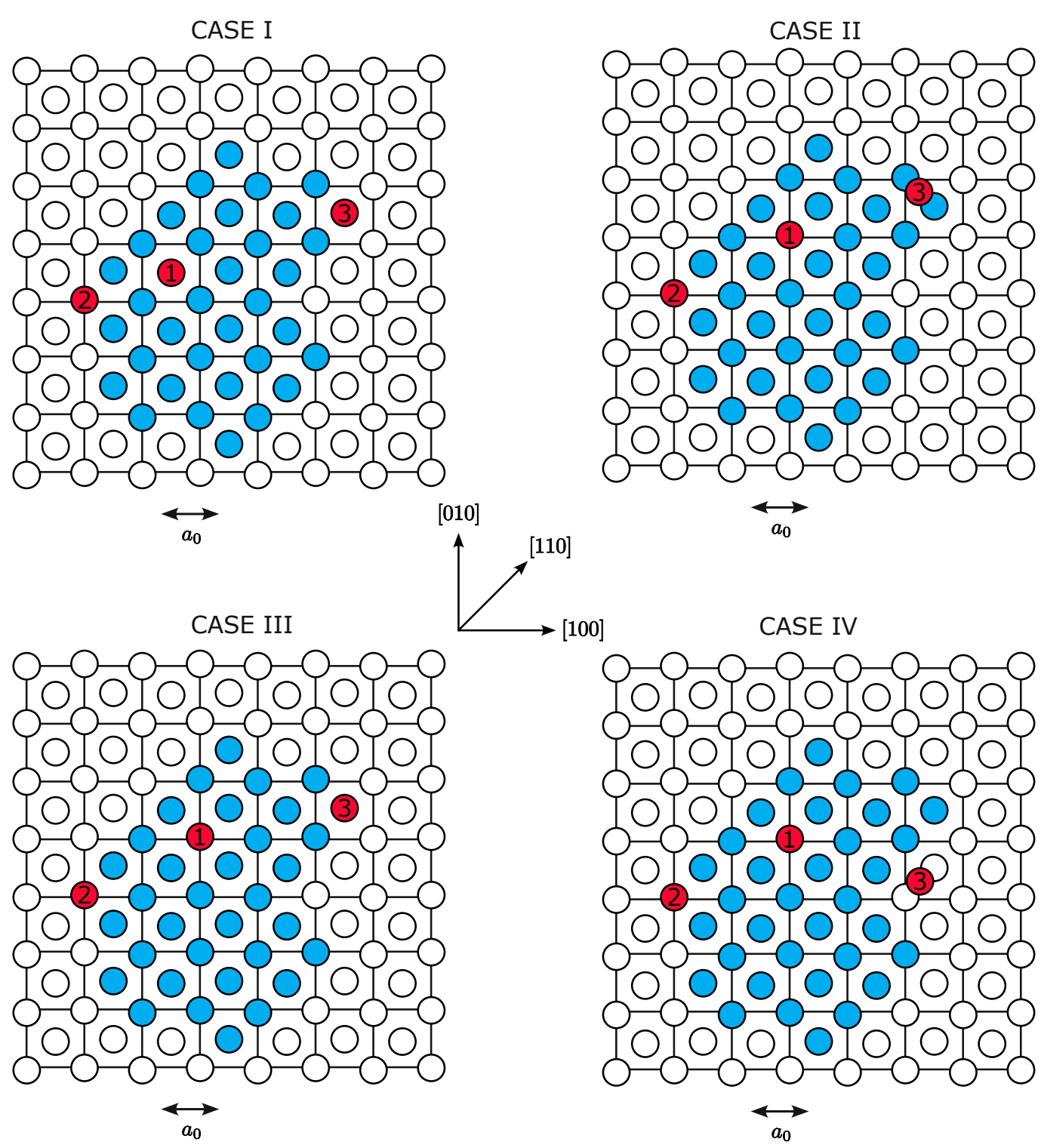

		A_1 (MHz)	A_2 (MHz)	A_3 (MHz)	Maximum nuclear dipole-dipole strength (Hz)
EXPERIMENT		197.5	45	15	31
NEMO-3D	CASE I	200.8	60.9	18.1	31
	CASE II	183.4	51.7	19.5	11
	CASE III	165.9	62.5	23.1	11
	CASE IV	179	55.3	19.9	11

FIG. S1. **Atomistic modelling of expected phosphorus atom configuration.** (Top) The shape of the patch of hydrogen which was removed during STM lithography of the left (L) register studied in this work is shown in blue. Any silicon atom could be replaced with a phosphorus atom during incorporation. In red, we show the expected final phosphorus atom locations in the patch, as modelled by NEMO-3D and found to have a close match to observed hyperfine strengths. We show here four best matches with experiments. The silicon lattice constant $a_0 = 0.543$ nm is shown for scale. CASE II and CASE IV each has one donor that is not placed in the same grid as the Si atoms and the other two donors. This is because we have also considered out-of-plane donor incorporation. Here in these two cases, the donors are one Si monolayer apart from the rest of the donors and the atoms in these layers are shifted by $(\pm 0.25, \pm 0.25)a_0$. (Bottom) A table comparing the experimentally observed hyperfines for the left register with those predicted by NEMO-3D for the configurations shown in red in the top Figure.

* These authors contributed equally to the work

The hyperfine values extracted from the Electron Spin Resonance (ESR) spectrum serve as a metrology tool for determining the phosphorus atom arrangement inside the multi-nuclear spin register. To calculate the hyperfine values, we use the semi-empirical tight-binding modeling tool NEMO-3D [33, 34] and compute the electronic wavefunctions in a system of three phosphorus (P) donors placed inside the silicon (Si) crystal. These wavefunctions are expressed at each lattice site in a spin resolved $sp^3d^5s^*$ atomic orbital basis. Each P atom is modeled as a single positive charge screened by the Si dielectric constant. At the P atom location, which is a substitutional lattice site, a cut-off potential U_0 is applied which represents the central-cell correction. Furthermore, the on-site energies of p and d orbitals are adjusted to give $\backsim 1\mu eV$ accuracy of the energy states [35]. We compute the hyperfine value for each P atom by evaluating the ground-state electron density ($|\psi(R_{donor})|^2$) at the P atom location. In addition to providing good agreement with experiments on single P atoms in Si [25, 36, 37], this method has been used before to successfully determine phosphorus atom arrangements in registers with many phosphorus atoms [22, 38, 55].

We consider all the possible 3P atom arrangements within a 2.7 nm × 1.9 nm area of Si layer, which is of similar size as the lithographic patch in the experiment. We also consider donor atoms incorporating out-of-plane, which can happen in these systems during silicon growth [22]. We limit our search to the cases where only one donor moves out-of-plane within two Si monolayers of the intended donor incorporation plane. There are a few arrangements that give close matches with the hyperfine values observed in the experiment as shown in S1. Among these arrangements, we consider CASE I to be the best possible candidate since, in addition to the close hyperfine matches with the experiment, this arrangement gives a nuclear dipole-dipole interaction strength of 31 Hz between the two donors located $1.58a_0$ ($a_0 = 0.543$ nm is the Si lattice constant) apart, similar to experimental results. The discrepancy could be due to nearby charge defects and/or leakage of the wavefunction to the nearby gates/SET.

II. QUANTUM NON-DEMOLITION MEASUREMENT PROCEDURE OF FULL REGISTER

The Quantum Non-Demolition (QND) measurements used in Fig. 2 leverage the nuclear-configuration dependence of ESR frequencies to establish the nuclear spin state. We start by loading an electron-$\downarrow$ using Elzerman initialisation [40]. By then applying an adiabatic inversion of ν_1^e, we invert the electron only if the nuclear spins are in the configuration $|\Downarrow\Downarrow\Downarrow\rangle$. If the electron is then measured, a detection of electron-$|\uparrow\rangle$ represents a measurement of the nuclear spins being in state $|\Downarrow\Downarrow\Downarrow\rangle$, whereas a detection of electron-$|\downarrow\rangle$ means that nuclei could be in any state except $|\Downarrow\Downarrow\Downarrow\rangle$. This procedure (initialisation - inversion - measurement) represents a quantum non-demolition (QND) measurement of the nuclear spin configuration because the nuclei are left in the measured nuclear spin state after measurement is complete.

Multiple QND readouts can be performed, with the nuclear spins remaining in the measured state. As described in the main text, this allows for measurements to be performed over all ESR transitions, and hence all nuclear spin configurations, as illustrated in Fig. 2a of the main text. While this procedure can be used to determine the nuclear spin configuration by picking whichever peak is most likely to produce a measurement of electron-$|\uparrow\rangle$, we are more interested in examining the evolution of each nuclear spin individually. If nuclear spin 1 is $|\Downarrow\rangle$, then regardless of the evolution of the other nuclei, one of the transitions ν_1^e, ν_2^e, ν_3^e and ν_4^e must have a high chance of inverting the electron, and hence producing a measurement of electron-$|\uparrow\rangle$, since these transitions all have nuclear spin 1 in state $|\Downarrow\rangle$ (see Fig. 1d). If other nuclei have high error, it is also possible for multiple peaks to invert the electron rather than just one. In contrast to transitions ν_1^e, ν_2^e, ν_3^e and ν_4^e, the transitions ν_5^e, ν_6^e, ν_7^e and ν_8^e must all have a low chance of inverting the electron, as these have nuclear spin 1 in state $|\Uparrow\rangle$. Hence, by comparing the total probability of detecting electron-$|\uparrow\rangle$ for transitions $\{\nu_1^e, \nu_2^e, \nu_3^e, \nu_4^e\}$ with the total probability of detecting electron-$|\uparrow\rangle$ for transitions $\{\nu_5^e, \nu_6^e, \nu_7^e, \nu_8^e\}$, we produce a Random Telegraph Signal (RTS) trace for nuclear spin 1, independent of the behaviour of the other two nuclear spins, as per the formula in main text Eq. 4.

By comparing probabilities of detecting electron-$|\uparrow\rangle$ for $\{\nu_1^e, \nu_2^e, \nu_3^e, \nu_4^e\}$ to $\{\nu_5^e, \nu_6^e, \nu_7^e, \nu_8^e\}$, if electron SPAM fidelities or ESR adiabatic inversion probabilities vary, the nuclear spin state can still be determined. In addition, in the case of complete electron readout failure (eg. the period between $\sim$ 110 s and 115 s in Fig. 2b-d), there is no longer any difference between the probability of detecting electron-$|\uparrow\rangle$ for $\{\nu_1^e, \nu_2^e, \nu_3^e, \nu_4^e\}$ compared to $\{\nu_5^e, \nu_6^e, \nu_7^e, \nu_8^e\}$, which means that such readout failures can be detected using double thresholding (see Supporting Information III). In contrast to this method, if just the transitions $\{\nu_1^e, \nu_2^e, \nu_3^e, \nu_4^e\}$ were driven (and not $\{\nu_5^e, \nu_6^e, \nu_7^e, \nu_8^e\}$), complete electron readout failure would cause a period where the nuclear spin is always classified as $\Uparrow$, as no ESR peaks will detect electron-$|\uparrow\rangle$. As such, when measuring nuclear spin 1 in Fig. 2 of the main text, we compare the transitions $\{\nu_1^e, \nu_2^e, \nu_3^e, \nu_4^e\}$ and $\{\nu_5^e, \nu_6^e, \nu_7^e, \nu_8^e\}$, rather than examining $\{\nu_1^e, \nu_2^e, \nu_3^e, \nu_4^e\}$ only. The same is true for the corresponding transitions for nuclear spins 2 and 3, and allows measurement of the nuclear spin states in a way robust against SPAM and ESR inversion variability.

III. DOUBLE-THRESHOLDING PROCEDURE FOR NUCLEAR SPIN READOUT USED FOR DETERMINING NUCLEAR SPIN LIFETIMES

To read out the state of the full register, QND measurements are performed as outlined in Supporting Information II. Once this data has been taken, to determine the configuration of the nuclear spins one must apply some thresholding procedure to the random telegraph signal (RTS) traces $R_A^{(i)}(t)$ obtained in the main text Fig. 2b-d. When measuring the lifetimes of the nuclear spins in Fig. 2, we design a procedure which is robust periods of electron readout failure (eg. the period between 110-115 s in Fig. 2b-d), so that such failures do not artificially shorten the nuclear spin lifetimes. As such, a single-thresholded scheme which categorises nuclear spin $\Uparrow$ if $R_A^{(i)}(t) > 0.5$, else $\Downarrow$, is not ideal as during readout failure many artificial nuclear spin flips would be falsely detected.

Instead, we start by fitting histograms of the RTS traces to a triple-Gaussian model, as per Fig. 2e-g of the main text. A triple-Gaussian is required as a third peak arises at 0 due to electron readout failure. From these fits, we use the centres of the upper and lower Gaussian distributions as upper and lower thresholds. That is, a given spin will only become $\Uparrow$ if $R_A^{(i)}(t)$ exceeds the upper threshold, and similarly will only become $\Downarrow$ once $R_A^{(i)}(t)$ falls below a lower threshold. During readout failure, the signal is close to 0.5, and so is unlikely to cross the opposing threshold (which would cause a nuclear spin flip to be detected when there likely was not a nuclear spin flip). However, if electron readout is functioning, and the nuclear spin flips, there is still a 50 % chance per shot for the thresholded nuclear spin state to change (since we have taken the centre of the upper and lower Gaussians), meaning that after an average of 2 shots the correct nuclear state will be established.

An important caveat here is that if nuclear spin errors are high, for example as is the case with nuclear spin 2, the nuclear spin could flip before $R_A^{(i)}(t)$ crosses the appropriate threshold. As such, for nuclear spins with high error the lifetimes calculated using this method will give an over-estimate. Nuclei with high error will also have their lifetime extended due to the lack of flips detected during electron readout failure. Ultimately these factors produce an over-estimate of the lifetimes of nuclear spin 2. As such, the lifetimes of $T_{\Uparrow}^{(2)} \leq 1.4$ s and $T_{\Uparrow}^{(2)} \leq 0.9$ s reported in Fig. 2i should be taken as an upper bound.

IV. ESTIMATING NUCLEAR SPIN READOUT ERRORS FROM READOUT HISTOGRAMS

To estimate the error of nuclear spin readout in Fig. 2e-g of the main text, we use the triple-Gaussian fitted to the observed nuclear spin readout histograms, in conjunction with the observed lifetimes of each nuclear spin. Firstly, we estimate the error from the double thresholding procedure by integrating the part of the Gaussian for the upper threshold ($\mathcal{N}(\mu_u, \sigma_u)$) which crosses the lower threshold, as well as the same for the lower threshold ($\mathcal{N}(\mu_l, \sigma_l)$):

$$\epsilon_{\text{thresh}} = \frac{1}{2}\int_{-\infty}^{\mu_l} \frac{1}{\sigma_u\sqrt{2\pi}} e^{-\frac{1}{2}\left(\frac{x-\mu_u}{\sigma_u}\right)^2} dx + \frac{1}{2}\int_{\mu_u}^{\infty} \frac{1}{\sigma_l\sqrt{2\pi}} e^{-\frac{1}{2}\left(\frac{x-\mu_l}{\sigma_l}\right)^2} dx \tag{17}$$

where each term is weighted by $\frac{1}{2}$, as the nuclear spin can only be in one of the states $\Uparrow$ or $\Downarrow$.

In addition, we factor in the error due to the limited nuclear spin lifetimes. To calculate an average error, we average the nuclear lifetimes for $\Uparrow$ and $\Downarrow$ given in Fig. 2h-j to produce an average $T_{\Downarrow/\Uparrow}$ for each nuclear spin. Then, we calculate the probability that the nuclear spin will flip during an $N = 30$ readout sequence according to the distribution of lifetimes:

$$\epsilon_{\text{lifetime}} = 1 - e^{-0.008N/T_{\Downarrow/\Uparrow}} \tag{18}$$

where 0.008 is the time, in seconds, of a single readout ($N = 1$) of the nuclear spin configuration. Hence, we determine the fidelity of readout f_R for a given nuclear spin according to:

$$f_R = (1 - \epsilon_{\text{thresh}})(1 - \epsilon_{\text{lifetime}}) \tag{19}$$

since we must have no thresholding nor lifetime error to have a successful measurement. This method was used to find the fidelity of readout using the results taken in Fig. 2 of the main text, and is useful as a baseline to compare to the errors predicted from the dipolar coupling found later in the main text.

V. SUMMARY OF ERROR SOURCES AFFECTING PHOSPHORUS NUCLEAR SPINS IN MULTI-NUCLEAR SPIN REGISTERS IN SILICON

In this section, we provide a summary of mechanisms causing (and respective rates of) nuclear spin flips for multi-nuclear spin registers in silicon, and also include for completeness a summary of the strengths and interaction terms for the nuclear dipolar and anisotropic hyperfine error mechanisms studied in this work.

For the purposes of this summary, we assume that the electron bound to the register is being used as the ancilla qubit to perform non-demolition readout, which is the case for the readout performed in this work. Under this assumption, nuclear spin flips could occur at three places during QND readout (recalling that such a process tunnels the electron off and then back onto the register): firstly, the nuclear spin(s) could flip while the electron is present on the register, secondly they could flip while the electron is not present (ie. the register is ionised), and finally there is a chance for the nuclear spin(s) to discretely flip upon each tunnelling event. To characterise the nuclear spin flip probability, we specifically examine nuclear spins in their eigenbasis with the electron loaded, comparing the state populations before and after a single $\mathrm{CR}\binom{0}{1}$ QND readout operation (including electron re-initialisation), and maximise the probability that the nuclear state is the same before and after readout. This is what is done throughout this work, but we define this assumption directly as it disagrees with the convention of some previous literature (eg. [8, 29]) while agreeing with other previous literature (eg. [28]). Our summary is restricted exclusively to the measurement of the Z-projection of the nuclear spins of the register, because the phase of the nuclear spins is extremely sensitive to the exact duration of measurement and requires more sophisticated methods of readout [18].

A. Nuclear spin flips while electron is loaded

The first type of nuclear spin flip that we seek to characterise is when the electron is loaded on the register. The main mechanism which contributes to nuclear spin flips in this case is the relaxation pathway $|\widetilde{\uparrow\Downarrow}\rangle \to |\widetilde{\downarrow\Uparrow}\rangle$ which releases a phonon to the environment via the contact hyperfine interaction, as studied by Hsueh *et. al.* [41]. Importantly, this phonon-relaxation pathway is not the dominant source of nuclear spin flips during QND readout, and so can be largely ignored. This is because phonon relaxation exhibits the following general behaviours:

- For fields $B_0 \sim 1.5$ T, the rate of this relaxation is on the order of $\Gamma \sim 5\times10^{-3} - 5\times10^{-2}$ Hz.
- For low fields ($\lesssim 3$ T, depending on donor location specifics), the relaxation rate is proportional to B_0^3.
- The relaxation is proportional to the strength of hyperfine, A_i, as well as the strength of hyperfine for the excited orbital, $A_i^{(e)}$

As is plotted in Fig. S2, for typical readout durations, the rate of $\Gamma \sim 5\times10^{-3} - 5\times10^{-2}$ Hz contributes a nuclear flip probability of $\sim 10^{-6}$ error per QND readout event, which is significantly lower than other error sources. Moreover, this relaxation pathway is only available when the electron is $|\uparrow\rangle$, and so the contribution of phonon relaxation to the nuclear flip rate primarily depends on the duration over which the electron is $|\uparrow\rangle$ during QND measurement. When performing these simulations, we assume that at 1.5 T and 100 MHz hyperfine, the relaxation rate is 10^{-2} Hz (typical value per Hsueh *et. al.*), which is then scaled using the above rules. We also assume that four ESR pulses are performed consecutively (per readout in Fig. 3 of the main text), each with an inversion time of 200 μs.

B. Nuclear spin flips while register is ionised

The second nuclear spin flip pathway is for idling nuclear spins when the electron is not loaded on the register, or in other words the register is ionised. These errors have not been studied extensively in previous work on multi-nuclear spin registers in silicon. However, these errors are possible due to the direct nuclear-nuclear dipolar coupling studied in this work (see main text and Supporting Information VI), which occurs due to the small distances between phosphorus atoms within the register ($\sim 0.8 - 1.6$ nm). The nuclear-nuclear dipolar interaction is suppressed when the electron is loaded, due to the energy scale of the dipolar interaction, $\sim 10 - 100$ Hz for pairs of phosphorus atoms on the same multi-nuclear spin register, being much smaller than other energy scales of the system. In contrast, when the register is ionised the dipolar coupling allows antiparallel nuclear spins to oscillate between $|\Downarrow\Uparrow\rangle$ and $|\Uparrow\Downarrow\rangle$; however, these flip-flops can be significantly minimised by shortening the duration the register spends in the ionised state, which is possible to engineer using atomic-precision fabrication techniques [31] to control electron tunnelling rates. Specifically,

in the case where nuclei i and j are antiparallel, the chance for these nuclei to flip due to the dipolar interaction is given by:

$$\epsilon \approx \frac{1}{2}\left(b_D^{i,j}\right)^2 T_{\text{ion.}}^2 \tag{20}$$

where $b_D^{i,j}$ is the dipolar coupling strength between nuclei i and j ($b_D^{i,j} \sim 5-40$ Hz, observed at 6 and 33 Hz in this work), and $T_{\text{ion.}}$ is the time spent ionised during the electron readout. If the nuclei start parallel, the dipolar coupling will cause no effect to the polarisation of the nuclear spins. Because of the quadratic dependence on the ionisation time in Eq. 20, dipolar coupling errors dominate for devices with slow electron tunnelling times, but can be quadratically suppressed for faster devices. Specifically, for devices with tunnel rates on the order of 1 MHz (such as that reported by Keith *et. al.* [39]), these errors are on the order of 10^{-8}, and hence are negligible; whereas for slower tunnel rates on the order of 1 kHz (such as that reported by Broome *et. al.* [42]), dipolar coupling errors grow to order 10^{-2}, and have the ability to dominate nuclear spin flip rates. Because the device studied here also exhibits unusually slow tunnel rates (of ~ 5 kHz), these dipolar errors are indeed noticeable, contributing $\sim 8.6\times10^{-4}$ error rate as per the main text. This means that atomically-precise STM-based fabrication techniques [31] can ensure these dipolar flip-flops are negligible by shortening the tunnel gap between the register and reservoir, thereby minimising the time the register spends ionised during readout, which we propose should be done on future devices.

C. Nuclear spin flips due to tunnel events

The final nuclear spin flip mechanisms we consider are flips that occur due to the stochastic electron tunnelling events themselves. These spin flips are generally referred to as "ionisation shocks", because they are sudden discrete spin-flip events that happen for each tunnelling event that occurs (either ionising or loading the electron), rather than being continuous processes like the dipolar coupling or phonon relaxation discussed above. Ionisation shocks occur due to mixing of the nuclear eigenbases only while the electron is loaded, meaning that tunnelling events will project between two different sets of eigenbases, potentially causing nuclei to flip. While there are multiple types of ionisation shock, as we will see they can all be mitigated through tuning of the B_0 static magnetic field, or by tuning the hyperfine coupling strength A_i through deterministic donor placement [31] or the Stark shift effect [59].

The first type of ionisation shock we consider here was originally studied by Pla *et. al.* [8], which results because of mixing from the contact hyperfine interaction, a mechanism we refer to as one-spin ionisation shock (or just "ionisation shock"). Importantly, nuclear spin flips due to one-spin ionisation shock are not dominant over typical operating magnetic fields of $B_0 \sim 1.5$ T, because the mixing caused by the contact hyperfine is quadratically suppressed for higher magnetic fields. Specifically, the mixing is caused by the presence of $\hat{S}_x\hat{I}_x$ and $\hat{S}_y\hat{I}_y$ terms in the contact hyperfine Hamiltonian causes mixing between the $|{\uparrow\Downarrow}\rangle$ and $|{\downarrow\Uparrow}\rangle$ states of the eigenbasis while loaded. However, when the electron is removed during readout, this mixing is no longer present, and the nuclear states are projected onto the $\{|{\Uparrow}\rangle, |{\Downarrow}\rangle\}$ basis, which potentially causes a nuclear spin flip upon ionisation. The same is true when the electron is loaded back onto the register, with the mixing term diabatically reintroduced. While the difference in mixed and non-mixed eigenstates is small (typically exhibiting overlaps of $\sim 99.9\%$ [8]), a small chance remains for the nuclear spin to flip with each electron readout [8], referred to as contact hyperfine ionisation shock. As examined by Joecker *et. al.* [28], this mixing primarily facilitates the pathways $|\widetilde{\uparrow\Downarrow}\rangle \to |0\Uparrow\rangle$ and $|0\Downarrow\rangle \to |\widetilde{\downarrow\Uparrow}\rangle$ for electron $\uparrow$-out and $\downarrow$-in tunnel events respectively (where $|0\rangle$ represents the ionised state, ie. when the electron is not present on the register). The probabilities of these spin flips for each tunnelling event are given by:

$$\epsilon \approx \left(\frac{A}{2\gamma_e B_0}\right)^2 \tag{21}$$

where A is the contact hyperfine strength, γ_e is the electron gyromagnetic ratio, and B_0 is the constant magnetic field. The approximation here assumes $A, \gamma_n B_0 \ll \gamma_e B_0$ (where γ_n is the nuclear gyromagnetic ratio), with higher order corrections existing on order $O(\epsilon^2)$. Such corrections are typically not required for phosphorus atom spin systems with magnetic fields of $B_0 > 0.5$ T [28]. Note that each pathway requires the respective initial state (eg. initial state of $|\widetilde{\uparrow\Downarrow}\rangle$ for the pathway $|\widetilde{\uparrow\Downarrow}\rangle \to |0\Uparrow\rangle$), and will only have a chance of occurring for the corresponding electron tunnelling event (eg. electron $\uparrow$-out for the pathway $|\widetilde{\uparrow\Downarrow}\rangle \to |0\Uparrow\rangle$). This means that the probability in Eq. 21 is conditional on both the system starting in the relevant initial state and the relevant electron tunnel event

occurring, reducing the actual spin flip chance relative to Eq. 21. These Markovian errors implicitly assume that tunnel rates are significantly slower than the hyperfine coupling strength, ie. $A \gg 1/T_{\uparrow,\text{out}}, 1/T_{\downarrow,\text{in}}, 1/T_{\downarrow,\text{out}}, 1/T_{\uparrow,\text{in}}$ as discussed by Monir *et. al.* [29]. Due to the inverse quadratic scaling with respect to B_0 seen in Eq. 21, we will find that these errors do not dominate spin flips in multi-nuclear spin registers under typical operating fields of $B_0 \sim 1.5$ T, and indeed they do not explain the high errors observed in this work. When modelling this in Fig. S2, we assume that only the leading-order term in Eq. 21 is needed.

Another form of ionisation shock arises when two nuclear spins in the register have near-equal hyperfine interaction strengths. While this error source is noticeable on the lower end of typical operating fields ($B_0 \sim 0.5 - 1$ T), it can be minimised both by increasing the magnetic field and by engineering nuclear hyperfines (eg. through atomic-precision manufacturing [31]) to ensure nuclear spins all have significantly different hyperfine strengths. When two nuclei have near-equal hyperfines, they will experience an effective interaction mediated by the electron, which mixes the states $\{|\Uparrow\rangle, |\Downarrow\rangle\}$. Similar to one-spin ionisation shock, the interaction is turned on/off during electron tunnelling events, potentially causing nuclear spin flips. The two-spin ionisation shock probability for a single electron readout (tunnel off and tunnel on), provided the nuclear spins i and j are in an anti-parallel configuration, is given by:

$$\epsilon \approx \frac{1}{2}\left[\frac{A_i A_j}{\gamma_e B_0 (A_i - A_j)}\right]^2 \tag{22}$$

where A_i, A_j are the hyperfines of a pair of nuclear spins. Again, the leading-order approximation is that $A, \gamma_n B_0 \ll \gamma_e B_0$, but also that $|A_i - A_j| \gg A_i A_j/\gamma_e B_0$, true for the vast majority of hyperfines seen in multi-nuclear spin registers [29]. However, if A_i approaches A_j, then the probability for error approaches 1/2 [29]. Note that we do not consider higher-order degeneracy hotspots for which the error scales as $O\left((\gamma_e B_0)^{-3}\right)$ or smaller, as these would be so narrow as to dominate over a negligible fraction of parameter space. For two-spin ionisation shock, the flip rates given in Eq. 22 can be minimised if the difference of hyperfines $|A_i - A_j|$ can be maximised, which can be achieved through atomically-precise donor placement [31] and Stark shift [59]. Moreover, these two-spin ionisation shocks can be minimised by increasing the static B_0 magnetic field, due to the inverse quadratic dependence on B_0 in Eq. 22. In this work, such flips are indeed not the dominating source of error, because the two closest hyperfines (45 and 15 MHz between nuclear spins 2 and 3) are sufficiently far apart that this error is non-dominant (see Fig. S2). When modelling this error channel in Fig. S2, we assume that only the leading-order term in Eq. 22 is needed, which is violated when $A_i \sim A_j$; however, the region in which this assumption is violated is so narrow that for simplicity we cap the error rate to 1/4 (which is the maximum average error rate which can be achieved by two-spin ionisation shock, given the additional 50% chance that two nuclei start antiparallel in the first place).

The last kind of ionisation shock we consider is not mediated by the contact hyperfine interaction, but instead the anisotropic hyperfine (AHF) coupling between each nuclear spin and the bound electron which is present for multi-nuclear spin registers [25]. This interaction is typically ignored for multi-nuclear spin registers [25], but nonetheless in this work we find it to actually be the dominant error source for spin 2, as described in detail in the main text and Supporting Information XII. Such high errors are not typical, as we find that the nuclear spin flips caused by AHF ionisation shock fall only in narrow windows of magnetic field and hyperfine, meaning they can be readily mitigated by using similar techniques as for two-spin ionisation shock to avoid these error hotspots. To understand why these errors fall into hotpots, we need to understand how (and when) the AHF causes nuclear states to be mixed. The AHF introduces off-diagonal terms coupling the $|\uparrow\Downarrow\rangle$ and $|\uparrow\Uparrow\rangle$ states, and the $|\downarrow\Downarrow\rangle$ and $|\downarrow\Uparrow\rangle$ states. Similar to the contact hyperfine, such mixing is switched on/off during electron tunnel events, which projects (and potentially flips) the nuclear spins. Errors due to the AHF are minimal for single phosphorus atoms, as their hyperfine interaction is nominally isotropic [28] and so the AHF vanishes. However, in multi-nuclear spin registers, the anisotropic hyperfine interaction is present, and so to determine the mixing caused by the AHF we need to consider the relative strength of AHF coupling terms compared to the (diagonal) energy differences of the states that it mixes. For multi-nuclear spin registers, the AHF coupling terms are predicted to be around 3 orders of magnitude smaller than the contact hyperfine strength [25]. As contact hyperfine strengths are typically in the range of 10-100 MHz for multi-nuclear spin registers [27], this means anisotropic hyperfine strengths of around 10-100 kHz are expected. Including the diagonal energy differences for the relevant pairs of states, the probability of a nuclear spin to flip during an electron tunnel event is given by (Supplementary Information XII):

$$\epsilon = \frac{a^2}{1 + a^2} \tag{23}$$

where

$$a \equiv \frac{1}{\sqrt{4R^2+1}+2R} \tag{24}$$

$$R \equiv \frac{|\gamma_n B_0 \pm A/2|}{A_{\mathrm{AHF,M}}} \tag{25}$$

where A is the contact hyperfine strength, γ_n is the nuclear gyromagnetic ratio ($\gamma_n \sim -17.24$ MHz/T for phosphorus nuclei), B_0 is the global magnetic field, and $A_{\mathrm{AHF,M}}$ is the mixing term caused by the anisotropic hyperfine. When calculating R, the $+$ term corresponds to electron-$\downarrow$ tunnel events (either in or out), and the $-$ term corresponds to electron-$\uparrow$ tunnel events. Importantly, for particular values of B_0 and A, $\gamma_n B_0 + A/2$ can be near-zero, for example if $B_0 = 1.5$ T and $A \sim 50$ MHz. In the worst case where $\gamma_n B_0 + A/2 = 0$, this can cause errors of $\epsilon = 50$ % for electron-$\uparrow$ tunnel events, because at that point the $|\uparrow\Downarrow\rangle$ and $|\uparrow\Uparrow\rangle$ states are maximally mixed. In contrast, nuclear spin flips can be minimised provided that one maximises $|\gamma_n B_0 + A/2|$, which can be achieved by tuning the magnetic field B_0, or adjusting the hyperfine A using atomic-precision fabrication [31] or Stark shift [59]. When modelling the AHF ionisation shock errors in Fig. S2, we omit the chirped-ESR corrections considered in Supporting Information XIII, as these only contribute significantly in a small window around the hotspot, and are anyway plotted in Fig. 4g of the main text.

D. Minimising overall nuclear flip probability

With such a variety of mechanisms affecting QND readout of nuclear spins in multi-nuclear spin registers, it is important to understand under what circumstances each effect dominates, and more importantly, to understand what combined set device parameters will mitigate all factors contributing to nuclear flip rates. Importantly, flip rates can be optimised by considering only a few key parameters:

- the global magnetic field B_0, which affects phonon relaxation, as well as both contact and anisotropic hyperfine induced ionisation shock,
- the contact hyperfine strengths A_i of all nuclei on the dot, and
- the tunnel time $T_{\mathrm{ion.}}$ for electron $|\downarrow\rangle$ to tunnel onto the dot during readout.

This list is not exhaustive, since it does not include parameters like $A_{\mathrm{AHF,M}}$ and $b_D^{i,j}$, which depend on the exact locations of the donor nuclei. Despite not being exhaustive, the listed parameters are useful to consider as they can all be controlled during fabrication and measurement of devices.

To illustrate the dependence of the above three factors on nuclear flip rates, Fig. S2a, b and c show modelled nuclear flip rates for the device studied here as a function of magnetic field, hyperfine and tunnel time. All parameters used are taken for the device studied here, except that the default magnetic field is taken as 1.5 T in Fig. S2b,c to avoid the anisotropic hyperfine error dominating the entire parameter space shown. These simulations are taken as an average over measuring the nuclear $|\Uparrow\rangle$ and $|\Downarrow\rangle$ states, using the same readout technique as per Fig. 3 of the main text.

From the results in Fig. S2, we can see that different mechanisms are dominant over different parameter ranges, making it important to consider their combined effect when optimising nuclear flip rates. From Fig. S2a, we see that at very low magnetic fields, ionisation shock dominates flip probabilities, and that at very high fields phonon relaxation will dominate. We also see a clear hotspot where $B_0 = A_2/2|\gamma_n| \sim 1.3$ T, where AHF ionisation shock increases the error rate to a maximum of 50% - which must be avoided to keep flip rates low. This hotspot was measured directly in Fig. 4 of the main text. In Fig. S2b, we see that same AHF ionisation shock hotspot show up as a function of hyperfine when $A_2 = |2\gamma_n B_0|$ 51.5 MHz (noting our choice of $B_0 \sim 1.5$ T). We also see the two hotspots corresponding to two-spin ionisation shock at $A_2 = A_1 = 15$ MHz and $A_2 = A_3 = 197.5$ MHz. The remaining errors tend to increase for higher values of hyperfine due to both ionisation shock and phonon relaxation, however these are largely dwarfed by the errors caused by two-spin ionisation shock and AHF ionisation shock. Finally, in Fig. S2c we see an increase in error for longer tunnel times, mainly due to dipolar coupling but also partially due to phonon relaxation; however, more importantly we see that errors due to dipolar coupling can be readily mitigated if tunnel times are reduced. Notably, in Fig. S2a and b, the worst errors occur at hotspots (locations labelled in the figures), with other error sources typically around $10^{-4} - 10^{-3}$ (with more typical operation $\sim 10^{-4}$ possible if the dipolar coupling errors where reduced by choosing a different assumed tunnel rate in these figures). These hotspots result from two-spin ionisation shock and AHF ionisation shock, with maximum error probabilities of 25% and 50% respectively. By avoiding these hotspots, we can keep nuclear spin flip rates minimal and therefore maximise QND

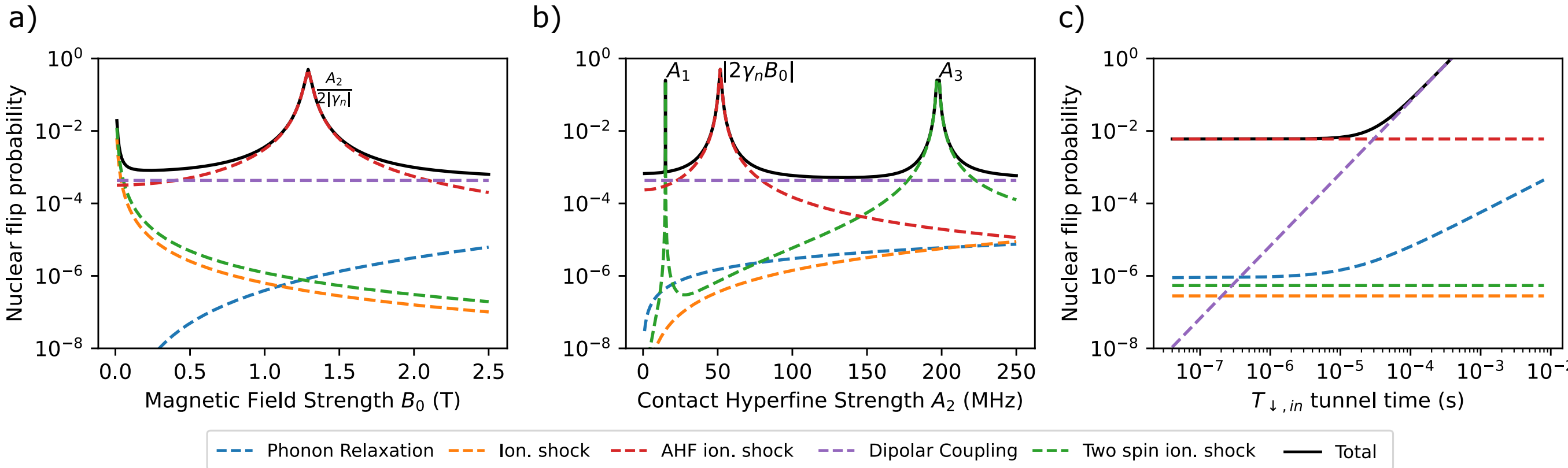

FIG. S2. **Summary of different mechanisms contributing to nuclear spin flip probabilities in multi-nuclear spin registers.** The total nuclear flip probabilities (solid lines) are shown as a function of (a) magnetic field strength B_0, (b) contact hyperfine strength A_2, and (c) the ↓-in tunnel time $T_{\downarrow,\text{in.}}$, averaged over initial nuclear states. Note that other tunnel rates are scaled by the same factor as $T_{\downarrow,\text{in.}}$ in (c). Individual nuclear flip pathways, as summarised in Supporting Information V, are shown as dashed lines. All device parameters are taken from the device studied here, except for the magnetic field in (b) and (c) which is taken as 1.5 T to avoid the AHF ionisation shock dominating errors.

SPAM fidelities, as defined quantitatively in the main text.

VI. NUCLEAR-NUCLEAR DIPOLAR SPIN DYNAMICS

The nuclear spins which are used as data qubits in multi-nuclear spin registers in silicon have magnetic dipole moments, which are used to encode the qubit state. However, magnetic dipoles in close proximity will experience a dipole-dipole interaction [43–45]. Here, we outline the form of this interaction and how it affects the nuclear spin flip rates during measurement.

A. Nuclear-Nuclear Dipolar Coupling Hamiltonian

For a pair of dipolar-coupled nuclear spins i and j, the direct through-space magnetic dipolar coupling is given by

$$H_D^{(i,j)} = b_D^{(i,j)} \left[\mathbf{I}^{(\mathbf{i})} \cdot \mathbf{I}^{(\mathbf{j})} - 3 \left(\mathbf{I}^{(\mathbf{i})} \cdot \hat{\mathbf{r}}_{\mathbf{ij}} \right) \left(\mathbf{I}^{(\mathbf{j})} \cdot \hat{\mathbf{r}}_{\mathbf{ij}} \right) \right] \tag{26}$$

where

$$b_D^{(i,j)} = \frac{1}{\hbar} \frac{\mu_0 (\hbar \gamma_n)^2 \hbar}{4\pi r_{ij}^3} \tag{27}$$

and $\mathbf{r_{ij}}$ is the displacement vector between the two nuclei, with corresponding unit vector $\hat{\mathbf{r}}_{\mathbf{ij}} = (\sin\theta\cos\varphi, \sin\theta\sin\varphi, \cos\theta)$, where $\hat{z}$ is defined as the direction of the global magnetic field B_0, so that θ is the angle w.r.t $\hat{z}$. In Eq. 27, μ_0 is the vacuum permeability, $\hbar$ is the reduced Planck's constant, and γ_n is the nuclear gyromagnetic ratio.

Assuming that the nuclei involved in the interaction are phosphorus nuclei, we can evaluate the physical constants in Eq. 27 to obtain:

$$\frac{1}{\hbar} \frac{\mu_0 (\hbar \gamma_n)^2 \hbar}{4\pi r_{ij}^3} = 2\pi \cdot 122.886 \text{ Hz a}^3 \tag{28}$$

where we write the result in units of $a = 0.543095$ nm, which is the silicon lattice constant, and we use angular frequency natural units with $\hbar = 1$. For multi-nuclear spin registers in silicon, the phosphorus nuclei are typically around 1.5-3 a apart [25, 38], which according to Eq. 27 results in values of $b_D^{(i,j)} \sim 5 - 40$ Hz in linear frequency. Indeed, this range agrees with the dipolar coupling strengths observed here of 6 and 33 Hz in Fig. 3 of the main text.

Under the application of a sufficiently large magnetic field B_0, specifically where $\gamma_n B_0 \gg b_D^{(i,j)}$ (for a field of $B_0 = 1.4$ T, $\gamma_n B_0 \sim 24$ MHz, which is many orders of magnitude higher than the $b_D^{(i,j)} \sim 5 - 40$ Hz calculated above), the secular approximation can be applied. The secular approximation suppresses all $[H_D]_{x,y}$, $[H_D]_{x,z}$ and $[H_D]_{y,z}$ terms in Eq. 26, except for terms proportional to $\hat{\mathbf{I}}^{(\mathbf{i})} \cdot \hat{\mathbf{I}}^{(\mathbf{j})}$ [46]. When the electron is removed (ionised) from the register, as occurs during readout of the electron, this produces the following dipolar Hamiltonian:

$$H_D^{(i,j)} = \sum_{i<j} b_D^{(i,j)} \left[3 I_z^{(i)} I_z^{(j)} - \hat{\mathbf{I}}^{(\mathbf{i})} \cdot \hat{\mathbf{I}}^{(\mathbf{j})}\right] \tag{29}$$

where we have absorbed an extra angular factor of $\left(\frac{3\cos^2\theta_{i,j} - 1}{2}\right)$ into $b_D^{(i,j)}$:

$$b_D^{(i,j)} = \frac{1}{\hbar} \frac{\mu_0 (\hbar \gamma_N)^2}{4\pi |r_{i,j}|^3} \left(\frac{3\cos^2\theta_{i,j} - 1}{2}\right) \tag{30}$$

In contrast, when the electron is present, as is the case during application of gate operations to multi-nuclear spin registers, the degeneracy between the nuclear states $|\Uparrow\Downarrow\rangle$ and $|\Downarrow\Uparrow\rangle$ is lifted. By the secular approximation, the terms proportional to $\hat{\mathbf{I}}^{(\mathbf{i})} \cdot \hat{\mathbf{I}}^{(\mathbf{j})}$ are therefore suppressed when the electron is present, leaving only the $I_z^{(i)} I_z^{(j)}$ term:

$$H_D^{(i,j)} = \sum_{i<j} b_D^{(i,j)} \left[2 I_z^{(i)} I_z^{(j)}\right] \tag{31}$$

However, the dipolar interaction is on the order of $b_D^{(i,j)} \sim 5 - 40$ Hz, which is much lower than all energy scales of the system. As such, the dipolar interaction does not significantly affect nuclear spin dynamics when the electron is loaded, and only causes noticeable errors to the multi-nuclear spin register during readout of the electron.

B. System Evolution due to Nuclear-Nuclear Dipolar Coupling while Ionised and Idling

The presence of nuclear-nuclear dipolar coupling with the system ionised can create oscillations between nuclear spins. This section derives the evolution of pairs of dipolar-coupled nuclei while idling when the electron spin is removed. Such ionisation periods occur during electron readout.

The presence of an electron suppresses any effects from dipolar coupling, as discussed in Supporting Information VI A. However, when the electron is removed/ionised, the dipolar Hamiltonian H_D in Eq. 29 will take effect. Exponentiating this matrix, we find the following unitary in the two-nuclear $\{|\Uparrow\Uparrow\rangle, |\Uparrow\Downarrow\rangle, |\Downarrow\Uparrow\rangle, |\Downarrow\Downarrow\rangle\}$ spin basis:

$$\mathrm{Exp}\left(-i\hat{H}_D t\right) = \begin{bmatrix} e^{-ib_D t/2} & 0 & 0 & 0 \\ 0 & \frac{1}{2} + \frac{1}{2}e^{ib_D t} & -\frac{1}{2} + \frac{1}{2}e^{ib_D t} & 0 \\ 0 & -\frac{1}{2} + \frac{1}{2}e^{ib_D t} & \frac{1}{2} + \frac{1}{2}e^{ib_D t} & 0 \\ 0 & 0 & 0 & e^{-ib_D t/2} \end{bmatrix} \tag{32}$$

which gives the following probabilities of a nuclear-nuclear spin flip-flop, provided that the pair of nuclear spins start in an anti-parallel configuration:

$$P\Big(|\Uparrow\Downarrow\rangle \leftrightarrow |\Downarrow\Uparrow\rangle\Big) = \frac{1}{2} - \frac{1}{2}\cos(b_D t) \tag{33}$$

If the ionised NMR frequencies of the pair of spins is slightly non-degenerate by some amount Δ, the Hamiltonian in Eq. 29 becomes:

$$\hat{H}_D = \frac{1}{2}\begin{bmatrix} b_D & 0 & 0 & 0 \\ 0 & -b_D+\Delta & -b_D & 0 \\ 0 & -b_D & -b_D-\Delta & 0 \\ 0 & 0 & 0 & b_D \end{bmatrix} \tag{34}$$

which, when exponentiated, gives:

$$\mathrm{Exp}\left(-i\hat{H}_D t\right) = \begin{bmatrix} e^{-ib_D t/2} & 0 & 0 & 0 \\ 0 & -\frac{(-\Delta-\Omega)e^{i(b_D-\Omega)t/2}}{2\Omega} + \frac{(-\Delta+\Omega)e^{i(b_D+\Omega)t/2}}{2\Omega} & -\frac{b_D e^{i(b_D-\Omega)t/2}}{2\Omega} + \frac{b_D e^{i(b_D+\Omega)t/2}}{2\Omega} & 0 \\ 0 & -\frac{b_D e^{i(b_D-\Omega)t/2}}{2\Omega} + \frac{b_D e^{i(b_D+\Omega)t/2}}{2\Omega} & -\frac{(\Delta-\Omega)e^{i(b_D-\Omega)t/2}}{2\Omega} + \frac{(\Delta+\Omega)e^{i(b_D+\Omega)t/2}}{2\Omega} & 0 \\ 0 & 0 & 0 & e^{-ib_D t/2} \end{bmatrix} \tag{35}$$

$$= \frac{1}{2\Omega}\begin{bmatrix} 2\Omega e^{-ib_D t/2} & 0 & 0 & 0 \\ 0 & 2e^{ib_D}\left(\Omega\cos(\Omega t/2) - \Delta\sin(\Omega t/2)\right) & 2ib_D e^{ib_D}\sin(\Omega t/2) & 0 \\ 0 & 2ib_D e^{ib_D}\sin(\Omega t/2) & 2e^{ib_D}\left(\Omega\cos(\Omega t/2) + \Delta\sin(\Omega t/2)\right) & 0 \\ 0 & 0 & 0 & 2\Omega e^{-ib_D t/2} \end{bmatrix} \tag{36}$$

where $\Omega \equiv \sqrt{b_D^2 + \Delta^2}$. Using this, we calculate that if the nuclear spin configuration starts in an anti-parallel state, the probability for them to flip is given by:

$$P\Big(\,|{\Uparrow\Downarrow}\rangle \leftrightarrow |{\Downarrow\Uparrow}\rangle\,\Big) = \frac{b_D^2}{\Omega^2}\sin^2\left(\frac{\Omega t}{2}\right) \tag{37}$$

However, if we assume t is sufficiently short, specifically that $t \ll \frac{2}{\Omega}$, we can expand the $\sin^2$ term:

$$P\Big(\,|{\Uparrow\Downarrow}\rangle \leftrightarrow |{\Downarrow\Uparrow}\rangle\,\Big) \approx \frac{b_D^2}{\Omega^2}\frac{\Omega^2 t^2}{4} + O(\Omega^4 t^4) \tag{38}$$

$$= \frac{b_D^2 t^2}{4} \tag{39}$$

which, for $t \ll b_D$ agrees with Eq. 33. Conveniently, Eq. 33 has no terms of Δ or Ω, allowing us to ignore the effect of Δ in the analysis of nuclear flip-flop errors during electron readout, provided the condition $t \ll \frac{2}{\Omega}$ is upheld. Because readout is sufficiently short, we use this assumption when studying the errors affecting nuclei during readout; however, we cannot use this assumption for the long durations used to characterise b_D experimentally in Fig. 3 (where $t \sim \frac{1}{\Omega}$).

VII. SYSTEM EVOLUTION DUE TO NUCLEAR-NUCLEAR DIPOLAR COUPLING WHILE IONISED DURING A HAHN ECHO SEQUENCE

This section derives the frequencies of oscillation between nuclear spin states we expect to observe in a Hahn Echo sequence while the register is ionised. We consider two cases - firstly, the evolution of a pair of nuclear spins with identical/degenerate NMR frequencies, and secondly when this degeneracy is lifted.

A. Degenerate NMR Frequencies

Firstly, we assume perfect degeneracy between the NMR frequencies $\gamma_n B_0$ of all nuclear spins when the electron is removed/ionised, meaning that the relevant Hamiltonian H_D for modelling dipolar interactions is as given by Eq. 29, reproduced here for convenience:

$$H_D^{(i,j)} = \sum_{i<j} b_D^{(i,j)}\left[3I_z^{(i)}I_z^{(j)} - \hat{\mathbf{I}}^{(\mathbf{i})}\cdot\hat{\mathbf{I}}^{(\mathbf{j})}\right] \tag{40}$$

A Hahn Echo sequence is implemented as the series of pulses $(\frac{\pi_x}{2} - \tau - \pi_y - \tau - \frac{\pi_x}{2})$ [32], where $\frac{\pi_x}{2}$ and π_y represent NMR-induced rotations about the x and y axes of the Bloch sphere respectively by an angle of $\pi/2$ and π respectively, and τ represents the duration between such rotations. Note that since the NMR frequencies are assumed to be degenerate, only a single tone is needed to control all nuclei simultaneously. In the basis $\{|\Uparrow\Uparrow\rangle, |\Uparrow\Downarrow\rangle, |\Downarrow\Uparrow\rangle, |\Downarrow\Downarrow\rangle\}$, these rotations are given by the following matrices:

$$\frac{\pi_x}{2} = \frac{1}{2}\begin{bmatrix} 1 & -i & -i & -1 \\ -i & 1 & -1 & -i \\ -i & -1 & 1 & -i \\ -1 & -i & -i & 1 \end{bmatrix} \tag{41}$$

$$\pi_y = \begin{bmatrix} 0 & 0 & 0 & 1 \\ 0 & 0 & -1 & 0 \\ 0 & -1 & 0 & 0 \\ 1 & 0 & 0 & 0 \end{bmatrix} \tag{42}$$

During a Hahn Echo, the nuclear Zeeman-splitting has no effect on the final state, and neither do small detunings of nuclear resonance frequencies from the NMR frequencies used to drive the $\frac{\pi_x}{2}$ and π_x rotations [32]. As such, during the τ wait times, the only term in the Hamiltonian we need consider is the dipolar coupling between the nuclear spins:

$$\begin{aligned} H &= b_D\left(3I_z^{(1)}I_z^{(2)} - \overline{I}^{(1)} \cdot \overline{I}^{(2)}\right) \\ &= \frac{b_D}{2}\begin{bmatrix} 1 & 0 & 0 & 0 \\ 0 & -1 & -1 & 0 \\ 0 & -1 & -1 & 0 \\ 0 & 0 & 0 & 1 \end{bmatrix} \end{aligned} \tag{43}$$

which features the singlet-triplet eigenstates:

$$\begin{aligned} |T_+\rangle &= |\Uparrow\Uparrow\rangle \\ |T_0\rangle &= \frac{1}{\sqrt{2}}\left(|\Uparrow\Downarrow\rangle + |\Downarrow\Uparrow\rangle\right) \\ |S\rangle &= \frac{1}{\sqrt{2}}\left(|\Uparrow\Downarrow\rangle - |\Downarrow\Uparrow\rangle\right) \\ |T_-\rangle &= |\Downarrow\Downarrow\rangle \end{aligned} \tag{44}$$

Since each of these are eigenstates, we can express their evolution due to Eq. 43 through exponentiation of their energies:

$$\begin{aligned} |T_+\rangle(t) &= \begin{bmatrix} 1 \\ 0 \\ 0 \\ 0 \end{bmatrix} e^{-ib_D t/2} & |T_0\rangle(t) &= \frac{1}{\sqrt{2}}\begin{bmatrix} 0 \\ 1 \\ 1 \\ 0 \end{bmatrix} e^{ib_D t} \\ |S\rangle(t) &= \frac{1}{\sqrt{2}}\begin{bmatrix} 0 \\ 1 \\ -1 \\ 0 \end{bmatrix} & |T_-\rangle(t) &= \begin{bmatrix} 0 \\ 0 \\ 0 \\ 1 \end{bmatrix} e^{-ib_D t/2} \end{aligned} \tag{45}$$

Now that we understand the evolution of these states, we can examine how the state evolves during a Hahn Echo. First, let us consider starting in the state $|\psi\rangle = |\Uparrow\Uparrow\rangle = |T_+\rangle$. Using Eq. 41, the state after the first $\frac{\pi_x}{2}$ pulse is:

$$\begin{aligned} |\psi\rangle &= \frac{1}{2}\begin{bmatrix} 1 \\ -i \\ -i \\ -1 \end{bmatrix} \\ &= \frac{1}{2}\left(|T_+\rangle - \sqrt{2}i\,|T_0\rangle - |T_-\rangle\right) \end{aligned} \tag{46}$$

Using the phase evolution of the eigenstates given in Eq. 45, we find that the state after time τ is:

$$|\psi\rangle = \frac{1}{2}\begin{bmatrix} e^{-ib_D\tau/2} \\ -ie^{ib_D\tau} \\ -ie^{ib_D\tau} \\ -e^{-ib_D\tau/2} \end{bmatrix} \tag{47}$$

Applying the π_y rotation we get:

$$|\psi\rangle = \frac{1}{2}\begin{bmatrix} -e^{-ib_D\tau/2} \\ ie^{ib_D\tau} \\ ie^{ib_D\tau} \\ e^{-ib_Dt/2} \end{bmatrix} \tag{48}$$

Again, we use Eq. 45 to find the state after the second wait time τ:

$$|\psi\rangle = \frac{1}{2}\begin{bmatrix} -e^{-ib_D\tau} \\ ie^{2ib_D\tau} \\ ie^{2ib_D\tau} \\ e^{-ib_D\tau} \end{bmatrix} \tag{49}$$

$$\sim \frac{1}{2}\begin{bmatrix} -1 \\ ie^{3ib_D\tau} \\ ie^{3ib_D\tau} \\ 1 \end{bmatrix} \tag{50}$$

where Eq. 50 differs from Eq. 49 only by a global phase of $e^{ib_D\tau}$. Under the application of the final $\frac{\pi_x}{2}$ operation, we find the final state as:

$$|\psi\rangle = \frac{1}{2}\begin{bmatrix} -1+e^{3ib_D\tau} \\ 0 \\ 0 \\ 1+e^{3ib_D\tau} \end{bmatrix} \tag{51}$$

Which results in the following probabilities to observe $|\Uparrow\Uparrow\rangle$ and $|\Downarrow\Downarrow\rangle$:

$$P\left(|\Uparrow\Uparrow\rangle\right) = \frac{1}{2}\left[1-\cos\left(3b_D\tau\right)\right] \tag{52}$$

$$P\left(|\Downarrow\Downarrow\rangle\right) = \frac{1}{2}\left[1+\cos\left(3b_D\tau\right)\right] \tag{53}$$

and hence if we start in state $|\Uparrow\Uparrow\rangle$, as a function of the total waiting time 2τ, we expect the final state after a Hahn Echo to oscillate between $|\Uparrow\Uparrow\rangle$ and $|\Downarrow\Downarrow\rangle$ at an angular frequency of $\omega_{\Uparrow\Uparrow} = 3b_D/2$. Similarly, if we start in the state $|\Downarrow\Downarrow\rangle$ we expect the same frequency of oscillation.

If instead, we start in the state $|\psi\rangle = |\Uparrow\Downarrow\rangle$, we find the final state of:

$$|\psi\rangle = \frac{1}{2}\begin{bmatrix} 0 \\ 1-e^{ib_D\tau} \\ 1+e^{ib_D\tau} \\ 0 \end{bmatrix} \tag{54}$$

with corresponding probabilities of $|\Uparrow\Downarrow\rangle$ and $|\Downarrow\Uparrow\rangle$ as:

$$P\left(|\Uparrow\Downarrow\rangle\right) = \frac{1}{2}\left[1 - \cos\left(b_D\tau\right)\right] \tag{55}$$

$$P\left(|\Downarrow\Uparrow\rangle\right) = \frac{1}{2}\left[1 + \cos\left(b_D\tau\right)\right] \tag{56}$$

resulting in oscillations between $|\Uparrow\Downarrow\rangle$ and $|\Downarrow\Uparrow\rangle$ at an angular frequency of $\omega_{\Uparrow\Downarrow} = b_D/2$ as a function of total waiting time 2τ. Note that the frequency differs from the $|\Uparrow\Uparrow\rangle$ case (derived above) by a factor of 3. Hence, we expect that if the NMR frequencies are perfectly degenerate, then parallel initial states should be seen to oscillate at three times the frequency of anti-parallel initial states.

B. Non-Degenerate NMR Frequencies

If, instead, we assume that the nuclear spins do not have the same NMR frequency $\gamma_n B_0$, ie. that the degeneracy is lifted by an amount $\gg b_D^{(i,j)}$ due to some interaction with the environment, then the evolution of the nuclei behaves differently. During a Hahn echo, the register remains ionised for up to 75 ms, which means we can no longer assume the same dynamics as if the degeneracy was not lifted. Instead, we must now use the following dipolar Hamiltonian H_D which applies in the case of degeneracy lifting (as derived for the case of the electron being loaded in Eq. 31):

$$H_D^{(i,j)} = \sum_{i<j} b_D^{(i,j)}\left[2I_z^{(i)}I_z^{(j)}\right] \tag{57}$$

The lifting of degeneracy could be, for example, due to local nano-scale variations in magnetic field due to unwanted nearby spins, or any other unwanted interaction which affects some but not all nuclei. Note that we assume this splitting is smaller than the linewidth of NMR, so that ionised NMR can still be applied simultaneously to all nuclei, as is observed in this experiment.

In this case, the eigenvectors are instead the Zeeman eigenstates:

$$|\Uparrow\Uparrow\rangle \tag{58}$$

$$|\Uparrow\Downarrow\rangle \tag{59}$$

$$|\Downarrow\Uparrow\rangle \tag{60}$$

$$|\Downarrow\Downarrow\rangle \tag{61}$$

with corresponding evolution:

$$\begin{aligned} |\Uparrow\Uparrow\rangle(t) &= \begin{bmatrix}1\\0\\0\\0\end{bmatrix} e^{-ib_Dt/2} \qquad |\Uparrow\Downarrow\rangle(t) = \begin{bmatrix}0\\1\\0\\0\end{bmatrix} e^{ib_Dt/2} \\ |\Downarrow\Uparrow\rangle(t) &= \begin{bmatrix}0\\0\\1\\0\end{bmatrix} e^{ib_Dt/2} \qquad |\Downarrow\Downarrow\rangle(t) = \begin{bmatrix}0\\0\\0\\1\end{bmatrix} e^{-ib_Dt/2} \end{aligned} \tag{62}$$

After the first $\frac{\pi_x}{2}$ rotation, we get:

$$|\psi\rangle = \frac{1}{2}\begin{bmatrix}1\\-i\\-i\\-1\end{bmatrix} \tag{63}$$

which after time τ, according to Eq. 62 will evolve to

$$|\psi\rangle = \frac{1}{2}\begin{bmatrix} e^{-ib_D\tau/2} \\ -ie^{ib_D\tau/2} \\ -ie^{ib_D\tau/2} \\ -e^{-ib_D\tau/2} \end{bmatrix} \tag{64}$$

If we start in the initial state $|\Uparrow\Uparrow\rangle$, the final state under this hamiltonian is given by:

$$|\psi\rangle = \frac{1}{2}\begin{bmatrix} -1 + e^{2ib_D\tau} \\ 0 \\ 0 \\ 1 + e^{2ib_D\tau} \end{bmatrix} \tag{65}$$

which has probabilities

$$P\left(|\Uparrow\Uparrow\rangle\right) = \frac{1}{2}\left[1 - \cos\left(2b_D\tau\right)\right] \tag{66}$$

$$P\left(|\Downarrow\Downarrow\rangle\right) = \frac{1}{2}\left[1 + \cos\left(2b_D\tau\right)\right] \tag{67}$$

meaning that as a function of the total waiting time 2τ, we expect oscillations at an angular frequency of b_D. Similarly, if we start from $|\Downarrow\Downarrow\rangle$, we would have oscillations at an angular frequency of b_D.

If we start from $|\Uparrow\Downarrow\rangle$, we obtain the following final state:

$$|\psi\rangle = \frac{1}{2}\begin{bmatrix} 0 \\ -1 + e^{2ib_D\tau} \\ -1 - e^{ib_D\tau} \\ 0 \end{bmatrix} \tag{68}$$

which has probabilities

$$P\left(|\Uparrow\Downarrow\rangle\right) = \frac{1}{2}\left[1 - \cos\left(2b_D\tau\right)\right] \tag{69}$$

$$P\left(|\Downarrow\Uparrow\rangle\right) = \frac{1}{2}\left[1 + \cos\left(2b_D\tau\right)\right] \tag{70}$$

As a consequence, as a function of the total waiting time 2τ, we expect oscillations at an angular frequency of b_D. Similarly, if we start from $|\Downarrow\Uparrow\rangle$, we would have oscillations at an angular frequency of b_D.

In summary, as a function of the total waiting time 2τ, if the NMR frequencies are degenerate then we expect oscillations due to the dipolar interaction with angular frequencies of $3b_D/2$ and $b_D/2$ for parallel and antiparallel initial states respectively; and for non-degenerate NMR frequencies, we expect angular frequencies of b_D regardless of initial state. For the device studied here, the latter is the case, as justified in Supporting Information IX.

VIII. IMPROVING NUCLEAR SPIN 2 READOUT FIDELITY VIA POSTSELECTION FOR HAHN ECHO EXPERIMENT

For the data taken in Figure 3 of the main text, instead of individually reading each of the 8 ESR transitions as per Figure 2, each nuclear spin was measured individually using a series of 4 ESR pulses, followed by a measurement of the electron. The full nuclear readout involved $N = 3$ repetitions, with each repetition reading out each nuclear spin sequentially; so the full readout order was 1, 2, 3, 1, 2, 3, 1, 2, 3. Each of these readouts only sampled non-demolition readout of $\hat{I}_z^{(i)} =\Uparrow$, which minimises errors during readout by limiting the number of potential ionisation events and ESR pulses applied. In addition, before the full procedure a random electron was loaded onto the register, which

was then initialised using Elzerman initialisation. However, as nuclear spin 2 exhibits high errors, it was postselected to further improve its readout fidelity by only considering readouts where either 0/3 or 3/3 blips were recorded.

For these postselected readouts of nuclear spin 2, we assume that if the measurement is postselected on 0/3 or 3/3 of the measurements recording $|\uparrow\rangle$, then we only need to consider errors which could occur before the first measurement of nuclear spin 2, or after the last measurement of nuclear spin 2. Any single intermediate error on nuclear spin 2 would cause the first few measurements to record one result, and the rest of the measurements to record the opposite result; hence, the sequence would be discarded by the postselection.

We can further simplify the assumption that only errors before the first/after the last nuclear spin 2 measurement are relevant after postselection. We do this by leveraging the regularity of the readout order of 1, 2, 3, 1, 2, 3, 1, 2, 3. The errors which affect nuclear spin 2 after postselection are as follows:

- Ionisation shock errors from initialising the electron spin prior to the full measurement;
- Errors during the first readout of nuclear spin 1;
- Errors during the final ESR inversion and measurement of nuclear spin 2, which will still result in electron $|\uparrow\rangle$ being measured if nuclear spin 2 started $|\Uparrow\rangle$, which is still a correct measurement result;
- Errors during the last readout of nuclear spin 3.

These errors include all error sources from readout of nuclear spins 1, 2 and 3 exactly once; as such, they are equivalent to a single repetition of the readout procedure. The same argument can be extended to errors on the other two nuclei. As such, after postselecting nuclear spin 2 to include only measurements where 0/3 or 3/3 measurements recorded electron-$|\uparrow\rangle$, the error incurred on nuclear spin 2 is equivalent to the error incurred on it in only one readout repetition, but the readout fidelity (due to other error sources like electron readout) is equivalent or better than for 3 non-demolition readouts. Hence, the readout fidelity of nuclear spin 2 is improved by performing postselection. Overall, including postselection for both the initial and final readout steps of main text Fig. 3a, this procedure omits 68.4% of the original data taken.

For reference, the same data fitted in Fig. 3c-e of the main text without postselection of nuclear spin 2 is shown below in Fig. S3. Since the data is not postselected, nuclear spin 2 exhibits significant readout errors. This means that any true signal with nuclear spin 2 flipping will also give a small signal in traces without nuclear spin 2 flipping, and visa-versa. Hence, in Fig. S3a we see some erroneous oscillatory evolution in the curves $\Updownarrow_1\Downarrow_2$ and $\Downarrow_1\Updownarrow_2$, due to readout error on nuclear spin 2 making these curves imitate $\Updownarrow_1\Updownarrow_2$ and $\Downarrow_1\Downarrow_2$. In contrast, after postselection, Fig. 3c of the main text has no such oscillation in $\Updownarrow_1\Downarrow_2$ and $\Downarrow_1\Updownarrow_2$.

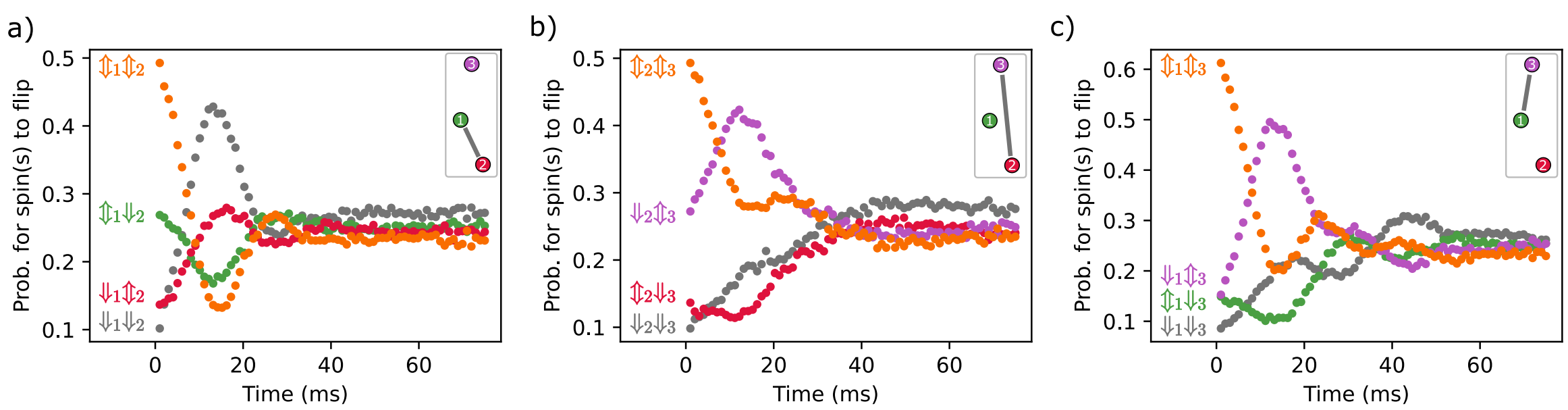

FIG. S3. **Dipolar oscillations shown in Fig. 3c-e of the main text, without postselection on nuclear spin 2.** The same plots as in Fig. 3c-e of the main text are reproduced here, but now include larger errors in nuclear spin 2, as the postselection (only taking readouts which involved nuclear spin 2 registering either 0/3 or 3/3 blips) is not applied. Similarly to main text Fig. 3c-e, (a) shows flips of nuclear spins 1 and 2, (b) shows flips of nuclear spins 2 and 3, and (c) shows flips of nuclear spins 1 and 3. The large errors for nuclear spin 2 are particularly visible because such errors cause oscillatory evolution for $\Updownarrow_1\Downarrow_2$ and $\Downarrow_1\Updownarrow_2$ in (a), which are not present in main text Fig. 3c.

IX. JUSTIFICATION OF LIFTED IONISED NUCLEAR FREQUENCY DEGENERACY IN THIS WORK

For the system studied here, we note that nuclear spins 1 and 2 (the pair of nuclei which have the strongest dipolar coupling) have non-degenerate NMR frequencies when the electron is ionised. Specifically, we note that the difference

in ionised NMR frequencies between nuclear spin 1 and nuclear spin 2 is $\gg b_D = 33$ Hz. We support this claim in two ways - firstly, with direct calibration of the qubit frequencies, and secondly, by the observed dipolar oscillation frequency.

A. Direct fine calibration of ionised NMR frequencies for nuclei 1 and 2

The first piece of evidence in support of a lifting of the degeneracy between the ionised NMR frequencies of nuclear spin 1 and nuclear spin 2 comes from a direct measurement of these NMR frequencies. To fine-tune the ionised NMR frequencies of all 3 nuclei, we used a modified version of the fast calibration used by Yang et. al. [49]. Specifically, we apply the pulse sequence illustrated in Fig. S4a, as a function of the rotation axis of the second NMR pulse (labelled ϕ). In the rotating frame of the applied drive, an idle qubit accumulates a phase according to

$$\hat{H}_{\text{idle}} = \delta_i \hat{I}_z^{(i)} \tag{71}$$

where δ_i is the detuning of nuclear spin i relative to the drive frequency. Over a wait time τ, the idle Hamiltionian $\hat{H}_{\text{idle}}$ in Eq. 71 implements a rotation about the z-axis of the Bloch sphere. In the absence of detuning, evaluation of the circuit in Fig. S4a produces sinusoidal oscillations in probability to flip as a function of ϕ:

$$P(\text{flip}) = \frac{1}{2} + \frac{1}{2}\cos(\phi) \tag{72}$$

However, the intermediate z-axis rotation caused by detuning in Eq. 71 will offset this sinusoid according to the amount rotated:

$$P(\text{flip}) = \frac{1}{2} + \frac{1}{2}\cos(\phi - \delta_i \tau) \tag{73}$$

Hence, we can use the phase offset $\delta_i \tau$ of the observed sinusoid, in conjunction with the applied wait time τ, to determine the detuning of each nuclear spin.

The results for nuclear spins 1-3 are given in Fig. S4b-d respectively, each with a wait time of $\tau = 500$ μs and an NMR drive frequency of $2\pi \cdot 23.954792$ MHz. We see that both nuclear spin 1 and nuclear spin 3 are close to resonance with the applied NMR frequency, but nuclear spin 2 is below resonance by $\sim 2\pi \cdot 200$ Hz. Such a splitting of $2\pi \cdot 200$ Hz satisfies $200 \gg b_D/2\pi = 33$ Hz, which suggests that the off-diagonal dipolar interaction terms are suppressed.

B. Inferred non-degeneracy through dipolar oscillation frequencies

The fine-tuned ionised NMR frequencies already suggest that nuclear spins 1 and 2 do not have the exact same NMR frequency when the electron is removed/ionised. However, we present additional evidence by examining the evolution of nuclei 1 and 2 during a Hahn echo.

As shown in Supporting Information VII, if the ionised NMR frequencies are degenerate, then parallel initial states will oscillate three times faster than antiparallel initial states, while if the NMR frequencies are degenerate, oscillations occur at the same frequency regardless of initial state. Hence, we can use the nuclear oscillation frequency for each initial state as an additional test for determining if ionised NMR frequencies are degenerate.

Figure 3c-e of the main text bins together flips of the same spin(s), for example binning together $|\Downarrow\Downarrow\rangle \rightarrow |\Downarrow\Uparrow\rangle$ and $|\Uparrow\Uparrow\rangle \rightarrow |\Uparrow\Downarrow\rangle$ as both include only the second nuclear spin flipping. However, to investigate whether the ionised NMR frequencies are degenerate or not, we do not bin in this way and instead investigate the dipolar evolution as a function of the initial state. These plots are shown in Fig. S5a-d, which mirrors the data in main text Fig. 3c, but are now separately plotted for all the possible initial states of nuclear spins 1 and 2. Specifically, Fig. S5a shows all traces contributing to $\Downarrow_1\Downarrow_2$ in Fig. 3c, and likewise Fig. S5b-d correspond to traces $\Downarrow_1\Updownarrow_2$, $\Updownarrow_1\Downarrow_2$, and $\Updownarrow_1\Updownarrow_2$ respectively in Fig. 3c. If the ionised NMR frequencies are degenerate, we should expect to see three times the oscillation frequency when nuclei start in states $|\Uparrow\Uparrow\rangle$ or $|\Downarrow\Downarrow\rangle$ when compared to oscillations starting in states $|\Uparrow\Downarrow\rangle$ or $|\Downarrow\Uparrow\rangle$. However, no such parity-dependent oscillation frequency is evident, instead showing identical frequency evolution regardless of

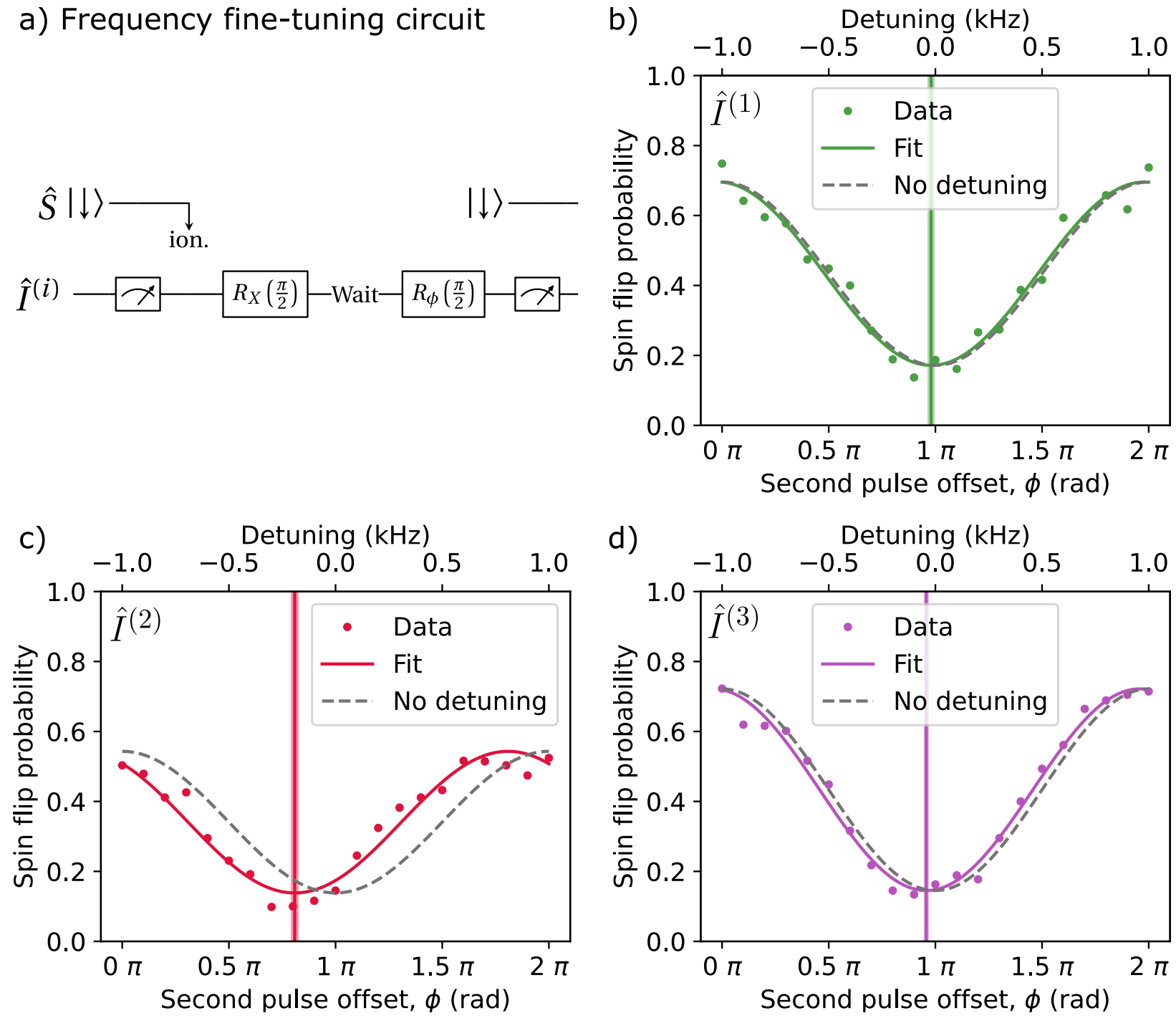

FIG. S4. **Fine-tuned calibration of ionised NMR frequencies.** After the approximate resonance frequency was been found, the circuit in (a) was applied separately to each of the nuclear spins studied here, as a function of the rotation axis of the second NMR pulse (labelled ϕ). The resulting probabilities of nuclear spin flips are given for (b) nuclear spin 1, (c) nuclear spin 2 and (d) nuclear spin 3, each taken with a wait time of 500 μs, and with an applied NMR frequency of $2\pi \cdot 23.954792$ MHz. Sinusoidal fits are performed on each trace, with a fixed period of 2π (solid curves). Based on the phase offsets of each curve, indicated by the vertical solid lines, one can determine the detuning of that nuclear spin (as per the upper axis labels, given as a linear frequency detuning). For reference, the fitted curve is also plotted with zero detuning (dashed grey line). We notice that the $2\pi \cdot 23.954792$ MHz drive frequency is on resonance with nuclear spins 1 and 3, but nuclear spin 2 is ~ 200 Hz below this drive frequency, supporting the claim the nuclear spin 1 and nuclear spin 2 have non-degenerate ionised NMR frequencies.

parity. The only differences observed are the different vertical offsets arising from nuclear readout errors. This further supports the claim that the ionised NMR frequencies of nuclear spin 1 and nuclear spin 2 are non-degenerate, and justifies the binning of flips of the same spin(s) used in the main text Fig. 3c-e.

X. SIMULATION OF IONISED NUCLEAR HAHN ECHO OSCILLATIONS

During a Hahn echo, we expect all pairs of nuclei to evolve according to the Hamiltonian

$$H_D = \sum_{i<j} b_D^{(i,j)} \left[2 I_z^{(i)} I_z^{(j)}\right] \tag{74}$$

However, in the experiment other sources of error are present. Specifically, there will be nuclear spin state preparation error, nuclear spin readout error, and dephasing errors on each nuclear spin. As such, to fit the observed oscillations in Fig. 3c-e, we model all these errors in addition to the evolution according to Eq. 74.

Specifically, the numerical model uses the following parameters:

- Dipolar coupling strengths $b_D^{(1,2)}$, $b_D^{(1,3)}$ and $b_D^{(2,3)}$ between all pairs of nuclei;
- Measurement fidelities $f_{m,\Uparrow}^{(i)}$ and $f_{m,\Downarrow}^{(i)}$ for measuring nuclear spin i correctly when it is $\Uparrow$ or $\Downarrow$ (respectively) before the final measurement;

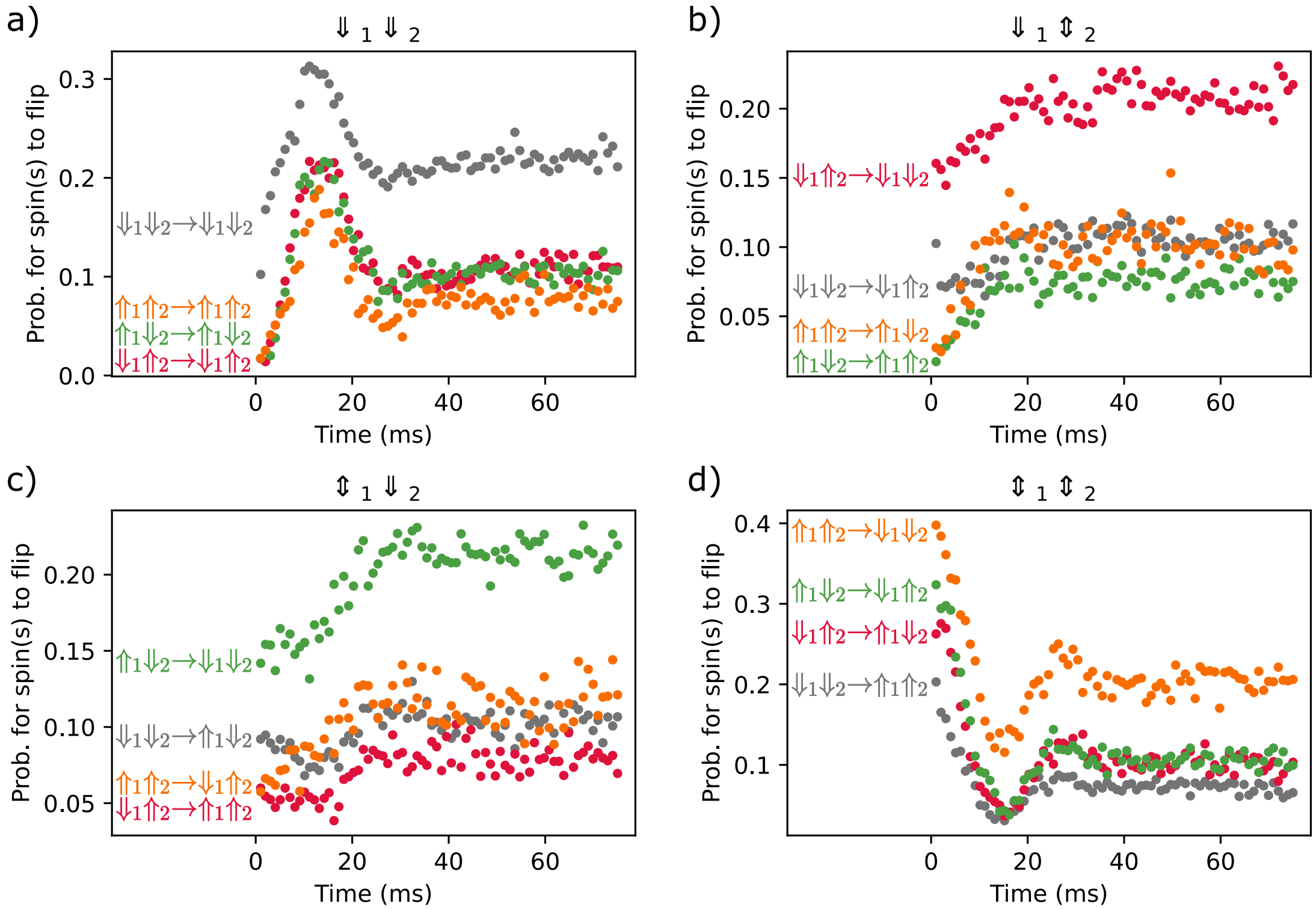

FIG. S5. **Dipolar oscillations shown in Fig. 3c of the main text, but without binning flips of the same spin(s).** The same data as in Fig. 3c of the main text is reproduced here, but without binning together flips of the same spin(s), eg. not binning together $|\Downarrow\Downarrow\rangle \to |\Downarrow\Uparrow\rangle$ and $|\Uparrow\Uparrow\rangle \to |\Uparrow\Downarrow\rangle$ since both flip the second nuclear spin. Specifically, we separately plot the individual traces used to construct (a) $\Downarrow_1\Downarrow_2$, (b) $\Downarrow_1\Updownarrow_2$, (c) $\Updownarrow_1\Downarrow_2$, and (d) $\Updownarrow_1\Updownarrow_2$ in Fig. 3c of the main text. In each sub-figure, the oscillation frequency appears identical, regardless of the initial state, further supporting the non-degeneracy of ionised NMR frequencies between nuclear spin 1 and 2. Differing readout fidelities between nuclei and between states $\Uparrow$ and $\Downarrow$ are responsible for the different vertical offsets between traces.

- State preparation fidelities $f^{(i)}_{sp,\Uparrow}$ and $f^{(i)}_{sp,\Downarrow}$ for nuclear spin i to be correctly $\Uparrow$ or $\Downarrow$ after the initial measurement, assuming that $\Uparrow$ or $\Downarrow$ is measured (respectively);
- The probability that readout spontaneously fails, $p_{\Downarrow\Downarrow\Downarrow}$, meaning that all nuclear spins are measured as $\Downarrow$ for that measurement (independent of any other measurements or the actual nuclear spin state);
- The probability of correlated readout failures $p_{c,\Downarrow\Downarrow\Downarrow}$, and an associated correlation time $\tau_{c,\Downarrow\Downarrow\Downarrow}$, such that for a measurement of duration t there is a $p_{c,\Downarrow\Downarrow\Downarrow}e^{-t/\tau_{c,\Downarrow\Downarrow\Downarrow}}$ chance for both the initial and final measurement to read all spins as $\Downarrow$;
- Dephasing times $T^{(i)}_{2,\mathrm{Hahn}}$ for each nuclear spin.

The model takes these parameters as input. Using whatever parameters are provided, we perform the following simulation to reconstruct the expected nuclear spin dynamics that would be measured.

Firstly, we simulate the evolution unitary of the nuclear spins in the absence of state preparation and measurement errors. We model the evolution by modifying Eq. 74. To model the Hahn-dephasing times, in the second wait time instead of simply exponentiating H_D, we now exponentiate a modified H'_D:

$$H'_D = H_D + \sum_i \delta_i \hat{I}^{(i)}_z \tag{75}$$

where δ_i is the difference in average detuning for each nuclear spin between the second and first wait times, taken in the rotating frame of the applied drive. This detuning is only applied to nuclear spins during the second wait time, because the Hahn echo pulse sequence will refocus any detuning which is the same in the first and second wait times. The detunings here are sampled according to a Gaussian with zero mean and with a standard deviation

$$\sigma^{(i)} = \frac{\sqrt{2}}{\pi T_{2,\text{Hahn}}^{(i)}} \tag{76}$$

from which values are generated according to Gaussian Inverse Transform Sampling (ITS) with $K = 201$ samples (taking an odd number to ensure the mean is sampled). These samples are then shuffled in a random order for each nuclear spin, so that only $K = 201$ simulations need to be performed (with each having detunings for each nuclear spin randomly-chosen from the sampled detunings), rather than having to perform $K^3 = 8.1 \times 10^3$ simulations for all combinations of sampled detunings. The random shuffling is seeded, so that the same curve is produced for the same input parameters, needed for stability of the fitting algorithm.

For each of these K simulations, elements of the resulting unitaries $U_{l,m}^k$ ($k \in [1, K]$) are then squared to produce $P_{l,m}^k = \left|U_{l,m}^k\right|^2$, the probability for the system to end in state l when starting in state m for detunings k. These probabilities are then averaged together over k to produce

$$P_{l,m} = \frac{1}{K}\sum_k P_{l,m}^k \tag{77}$$

which is the average probability for the system to end in state l when starting in state m over all sampled detunings, without readout errors.

After $P_{l,m}$ is calculated, SPAM errors are applied. Firstly, we apply the measurement fidelities $f_{m,\Uparrow}^{(i)}$ and $f_{m,\Downarrow}^{(i)}$, which are constructed as follows in the $\{|\Downarrow\rangle, |\Uparrow\rangle\}^{\otimes 3}$ basis:

$$F_m = \bigotimes_i \begin{bmatrix} f_{m,\Downarrow}^{(i)} & 1 - f_{m,\Uparrow}^{(i)} \\ 1 - f_{m,\Downarrow}^{(i)} & f_{m,\Uparrow}^{(i)} \end{bmatrix} \tag{78}$$

such that, if the true state of nuclear spin i is $|\Downarrow\rangle$, there is a $f_{m,\Downarrow}^{(i)}$ probability of measuring $|\Downarrow\rangle$ and a $1 - f_{m,\Downarrow}^{(i)}$ probability of measuring $|\Uparrow\rangle$, and similarly when starting $|\Uparrow\rangle$ (mutatis mutandis). Likewise, the state preparation errors are modelled as:

$$F_{sp} = \bigotimes_i \begin{bmatrix} f_{sp,\Downarrow}^{(i)} & 1 - f_{sp,\Uparrow}^{(i)} \\ 1 - f_{sp,\Downarrow}^{(i)} & f_{sp,\Uparrow}^{(i)} \end{bmatrix} \tag{79}$$

where now, if the *measured* spin state of nuclear spin i is $|\Downarrow\rangle$, the probability of the *true* spin state being $\Downarrow$ after measurement is $f_{sp,\Downarrow}^{(i)}$, and the probability of the *true* spin state being $\Uparrow$ after measurement is $1 - f_{sp,\Downarrow}^{(i)}$, and similarly when measuring $|\Uparrow\rangle$. These SPAM errors can be applied to the previously generated probabilities P (as per Eq. 77 as:

$$P \rightarrow F_m P F_{sp} \tag{80}$$

Next, we model the $\Downarrow\Downarrow\Downarrow$ readout errors. First, note that for longer experiments, if readout errors are no longer correlated (due to the typical correlation time $\tau_{c,\Downarrow\Downarrow\Downarrow}$), these errors will instead manifest as uncorrelated $\Downarrow\Downarrow\Downarrow$ readout errors. As such, for a total Hahn echo wait time t we make the following transformation:

$$p_{\Downarrow\Downarrow\Downarrow} \rightarrow p_{\Downarrow\Downarrow\Downarrow} + p_{c,\Downarrow\Downarrow\Downarrow}\left(1 - e^{-t/\tau_{c,\Downarrow\Downarrow\Downarrow}}\right) \tag{81}$$

$$p_{c,\Downarrow\Downarrow\Downarrow} \rightarrow p_{c,\Downarrow\Downarrow\Downarrow} e^{-t/\tau_{c,\Downarrow\Downarrow\Downarrow}} \tag{82}$$

$$\tag{83}$$

which allows us to simply use $p_{\Downarrow\Downarrow\Downarrow}$ to represent the probability for all uncorrelated readout failures (ie. individual readouts failing and measuring $\Downarrow\Downarrow\Downarrow$), and to use $p_{c,\Downarrow\Downarrow\Downarrow}$ to represent all correlated readout failures (where both the

initial and final measurements have a readout failure).

To model uncorrelated readout failures (ie. $p_{\Downarrow\Downarrow\Downarrow}$) of the initial measurement, we use the following:

$$F_{sp,\Downarrow\Downarrow\Downarrow} = \begin{bmatrix} \alpha+\beta & 0 & 0 & 0 & 0 & 0 & 0 & 0 \\ \beta & 1 & 0 & 0 & 0 & 0 & 0 & 0 \\ \beta & 0 & 1 & 0 & 0 & 0 & 0 & 0 \\ \beta & 0 & 0 & 1 & 0 & 0 & 0 & 0 \\ \beta & 0 & 0 & 0 & 1 & 0 & 0 & 0 \\ \beta & 0 & 0 & 0 & 0 & 1 & 0 & 0 \\ \beta & 0 & 0 & 0 & 0 & 0 & 1 & 0 \\ \beta & 0 & 0 & 0 & 0 & 0 & 0 & 1 \end{bmatrix} \tag{84}$$

where

$$\alpha = 1 - p_{\Downarrow\Downarrow\Downarrow} \tag{85}$$
$$\beta = p_{\Downarrow\Downarrow\Downarrow}/8 \tag{86}$$

which models that, if $\Downarrow\Downarrow\Downarrow$ is measured in the initial measurement, there is a $p_{\Downarrow\Downarrow\Downarrow}$ chance for the true state to be any (equally likely) state, and a $1-p_{\Downarrow\Downarrow\Downarrow}$ chance that the true state was in fact correctly measured as $p_{\Downarrow\Downarrow\Downarrow}$. Likewise, for uncorrelated readout failures of the final measurement, we use:

$$F_{m,\Downarrow\Downarrow\Downarrow} = \begin{bmatrix} 1 & p_{\Downarrow\Downarrow\Downarrow} & p_{\Downarrow\Downarrow\Downarrow} & p_{\Downarrow\Downarrow\Downarrow} & p_{\Downarrow\Downarrow\Downarrow} & p_{\Downarrow\Downarrow\Downarrow} & p_{\Downarrow\Downarrow\Downarrow} & p_{\Downarrow\Downarrow\Downarrow} \\ 0 & \alpha & 0 & 0 & 0 & 0 & 0 & 0 \\ 0 & 0 & \alpha & 0 & 0 & 0 & 0 & 0 \\ 0 & 0 & 0 & \alpha & 0 & 0 & 0 & 0 \\ 0 & 0 & 0 & 0 & \alpha & 0 & 0 & 0 \\ 0 & 0 & 0 & 0 & 0 & \alpha & 0 & 0 \\ 0 & 0 & 0 & 0 & 0 & 0 & \alpha & 0 \\ 0 & 0 & 0 & 0 & 0 & 0 & 0 & \alpha \end{bmatrix} \tag{87}$$

which represents that if the true state is any state other than $\Downarrow\Downarrow\Downarrow$ prior to the final measurement, there is a $1-p_{\Downarrow\Downarrow\Downarrow}$ chance that the state is measured correctly, and a $p_{\Downarrow\Downarrow\Downarrow}$ chance that $\Downarrow\Downarrow\Downarrow$ is measured instead. Equations 84 and 87 can now be used to update the matrix P obtained in Eq. 80:

$$P \to F_{m,\Downarrow\Downarrow\Downarrow} P F_{sp,\Downarrow\Downarrow\Downarrow} \tag{88}$$

Finally, we correct for correlated $\Downarrow\Downarrow\Downarrow$ readout failures. If a correlated readout error occurs, regardless of the previously calculated probabilities P, we will measure $\Downarrow\Downarrow\Downarrow$ for both the initial and the final measurement. As we wish for $P_{l,m}$ to remain a transition matrix of probabilities to measure l in the final measurement given m was measured in the initial measurement, correlated errors will only update the probabilities for when $m =\Downarrow\Downarrow\Downarrow$, as follows:

$$P_{l,m} \to \begin{cases} P_{0,0} + \sum_{l'\neq 0} (p_{c,\Downarrow\Downarrow\Downarrow} P_{l',0}) & \text{if } \ l = m = 0 \\ (1 - p_{c,\Downarrow\Downarrow\Downarrow}) P_{l,0} & \text{if } \ l \neq 0, m = 0 \\ P_{l,m} & \text{otherwise} \end{cases} \tag{89}$$

This update is applied to the probability matrix P obtained in Eq. 88 to produce the full modelled nuclear flip probabilities for the given set of parameters. This process is then repeated, according to a fitting algorithm, to find the best set of parameters which matches the data in Fig. 3c-e of the main text. To assist in fitting, the data which is fit is expanded to include the following:

- All $2^6 = 64$ individual traces $|\Downarrow\Downarrow\Downarrow\rangle \to |\Downarrow\Downarrow\Downarrow\rangle$, $|\Downarrow\Downarrow\Downarrow\rangle \to |\Downarrow\Downarrow\Uparrow\rangle$, ..., $|\Uparrow\Uparrow\Uparrow\rangle \to |\Uparrow\Uparrow\Uparrow\rangle$, weighted by a factor of 0.25;
- All 8 binned 3-qubit traces $\Downarrow\Downarrow\Downarrow$, $\Downarrow\Downarrow\Updownarrow$, $\Downarrow\Updownarrow\Downarrow$, ..., $\Updownarrow\Updownarrow\Updownarrow$, similar to those shown in Fig. 3c-e of the main text but with all 3 qubits rather than grouping oscillations in pairs, weighted by a factor of 0.5;
- All 6 single qubit traces $\Downarrow_1$, $\Updownarrow_1$, $\Downarrow_2$, ..., $\Updownarrow_3$, again similar to Fig. 3a-c but grouped for single spins rather than pairs, weighted by a factor of 0.6.

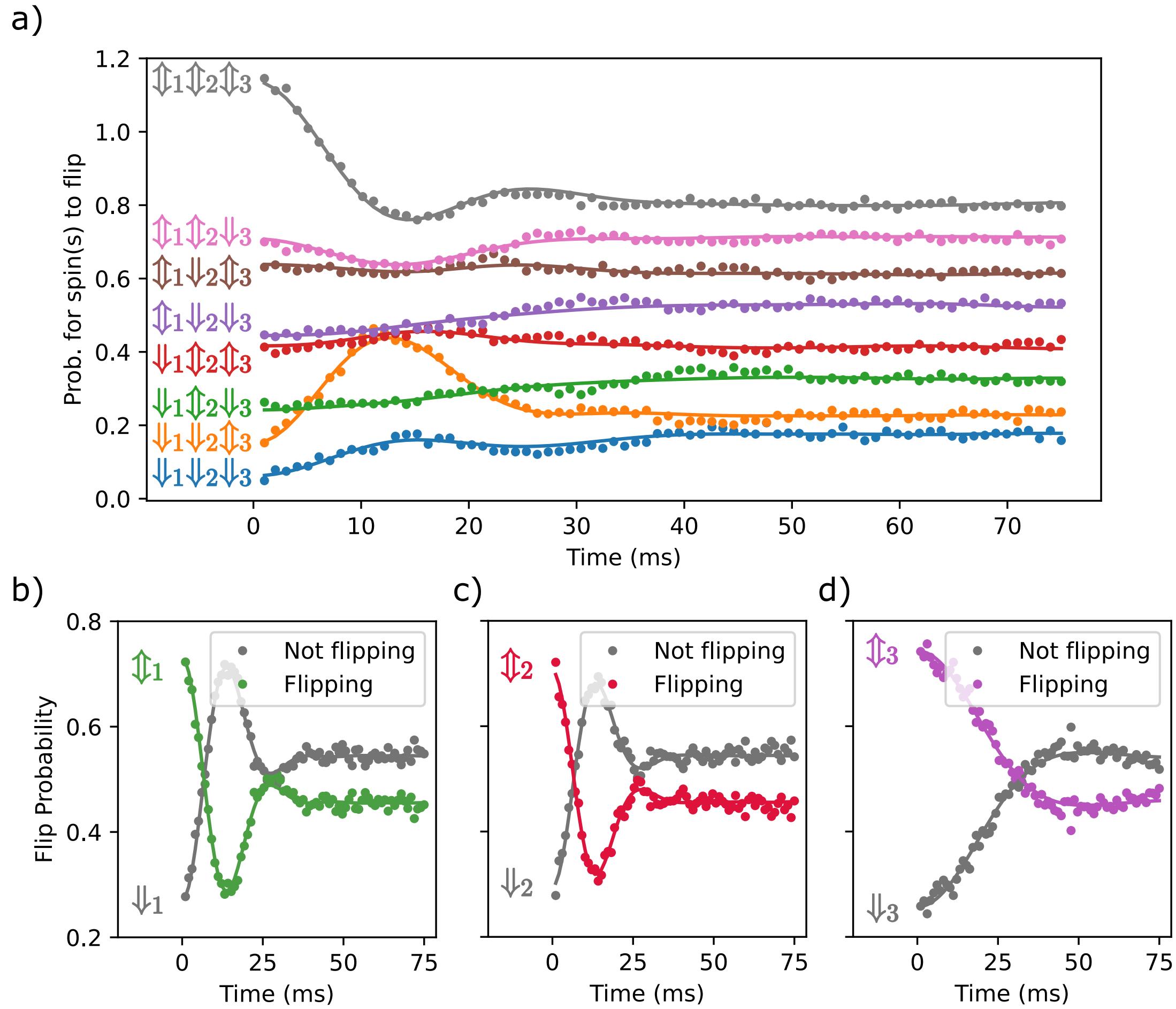

FIG. S6. **Additional data used to fit the dipolar oscillations in Fig. 3c-e of the main text.** In addition to the data in Fig. 3c-e of the main text, the fitting procedure was provided (a) all 8 binned 3-qubit traces, as well as the probabilities to measure (b) nuclear spin 1 flipping, (c) nuclear spin 2 flipping, and (d) nuclear spin 3 flipping individually. Note that the traces in (a) have been offset by progressive multiples of 0.1 for clarity. In addition to this data and the data in Fig. 3c-e of the main text, the fitting procedure was given all non-binned traces, as per the method outlined in Supporting Information X.

These extra traces are used to ensure that there is enough information to properly fit all parameters, and that there is a reduced chance of overfitting. Weighting is applied to each of these sets of data, scaling their contribution on the fit's objective function relative to the data shown in Fig. 3c-e of the main text. This weighting is used to place lower confidence on data with fewer averages (eg. the unbinned data), and to place higher emphasis on ensuring the correlations in Fig. 3c-e are properly fit. The fitted binned 3-qubit and single-qubit traces are shown in Fig. S6. Errors in the strength of the dipolar couplings are found by splitting the data into 5 equal-sized datasets, fitting each individually, and taking the standard deviation of the fitted parameters of each.

XI. ESTIMATION OF NUCLEAR SPIN 1 ERRORS FROM OBSERVED HISTOGRAMS

In Fig. 2h of the main text, we find that the average lifetime of nuclear spin 1 is 12.5 s. The lifetime of the nuclear spin T follows the distribution

$$P(T=t) = e^{-t/T^{(1)}_{\Downarrow/\Uparrow}} \tag{90}$$

If we let p be the probability that nuclear spin 1 has an error during an ionisation event, then the probability that no errors occur after n ionisations is given by

$$P = (1-p)^n \tag{91}$$

We now assume that error rates are sufficiently low that only one electron-$|\uparrow\rangle$ is measured, and hence only one ionisation event occurs, per set of 8 ESR transitions measured (ie. per single run of the circuit in Fig. 2a). This ignores when an error occurs for an early ESR transition, causing another ionisation event on a later ESR transition, but this effect is second-order in the nuclear error rate. Under this assumption, we can write that

$$n = t/\tau_R \tag{92}$$

where $\tau_R = 8$ ms is the time taken to measure all 8 ESR transitions. We can now equate Eqns. 90 and 91:

$$e^{-t/T^{(1)}_{\Downarrow/\Uparrow}} = (1-p)^{t/\tau_R} \tag{93}$$

which simplifies to

$$p = 1 - e^{-\tau_R/T^{(1)}_{\Downarrow/\Uparrow}} \tag{94}$$

which is used to estimate the average error rate of nuclear spin 1 in the main text.

XII. ANISOTROPIC HYPERFINE ERRORS

For multi-nuclear spin registers in silicon, the dominant interaction between the electron and the nuclear spins is the contact hyperfine interaction, determined by the value of the electron wavefunction at the locations of the phosphorus nuclei [25]. However, the electron also has a magnetic dipole moment, which causes an electron-nuclear interaction which depends on the larger shape of the electron wavefunction. Here, we outline the form of this interaction, and how it contributes to nuclear data qubit flips during readout of the electron spin.

A. Anisotropic Hyperfine Hamiltonian

The anisotropic hyperfine (AHF) interaction results from the through-space dipolar interaction between an electron spin and a nearby nuclear spin. In a multi-nuclear spin register, such AHF interactions couple the electron to each of the nuclear spins independently. Here, we discuss in detail the effect that the AHF interaction has on the dynamics of a multi-nuclear spin register in silicon.

First, we outline how the AHF interaction affects the electron-nuclear Hamiltonian. The standard Hamiltonian used when considering a multi-nuclear spin register, with a single electron loaded, is as follows [32]:

$$\hat{H}_{\mathrm{load}}(B_0) = \hat{H}_Z + \hat{H}_A = \left(\gamma_e \hat{S}_z + \sum_{i=1}^{n} \gamma_n \hat{I}_z^{(i)}\right) B_0 + \sum_{i=1}^{n} A^{(i)} \hat{\mathbf{S}} \cdot \hat{\mathbf{I}}^{(\mathbf{i})} \tag{95}$$

Where $\hat{H}_Z$ and $\hat{H}_A$ are the Zeeman and hyperfine Hamiltonians respectively, γ_e and γ_n are the electron and nuclear gyromagnetic ratios respectively (for multi-nuclear spin registers, the phosphorus gyromagnetic ratio is $\gamma_n/2\pi \approx -17.24$ MHz/T), n is the number of nuclear spins, $\hat{S}$ and $\hat{I}^{(i)}$ are the electron and i-the nuclear spin operators, B_0 is the global magnetic field strength, and $A^{(i)}$ is the contact hyperfine strength corresponding to nuclear spin i. However, in addition to the contact hyperfine terms $A^{(i)}$ presented in the usual Hamiltonian, there exist anisotropic hyperfine terms $\overleftrightarrow{A}^{(i)}_{AHF}$, which arise due to the dipolar interaction between the electron spin and the nuclear spins:

$$\hat{H}'_{\mathrm{load.}}(B_0) = \hat{H}_Z + \hat{H}_A + \hat{H}_B \tag{96}$$

$$\hat{H}_B = \sum_{i=1}^{n} \hat{\mathbf{S}} \cdot \overleftrightarrow{A}^{(i)}_{AHF} \cdot \hat{\mathbf{I}}^{(\mathbf{i})} \tag{97}$$

where $\overleftrightarrow{A_{AHF}^{(i)}}$ is the AHF tensor. Both the contact and anisotropic hyperfine terms are calculated from the electron wavefunction $|\Psi\rangle$ as follows [25]:

$$A^{(i)} = \frac{\mu_0}{4\pi}\gamma_e |\gamma_n| \frac{8\pi}{3} \left|\Psi\left(\mathbf{r^{(i)}}\right)\right|^2 \tag{98}$$

$$\left[\overleftrightarrow{A_{AHF}^{(i)}}\right]_{j,k} = \frac{\mu_0}{4\pi}\gamma_e |\gamma_n| \left\langle\Psi\middle|\frac{3(r_j - r_j^{(i)})(r_k - r_k^{(i)}) - \left|\mathbf{r} - \mathbf{r^{(i)}}\right|^2 \delta_{jk}}{\left|\mathbf{r} - \mathbf{r^{(i)}}\right|^5}\middle|\Psi\right\rangle \tag{99}$$

where μ_0 is the vacuum permeability, $\mathbf{r^{(i)}}$ is the position of the ith donor nucleus, $j,k \in \{x,y,z\}$ are electron position vector components, and δ_{jk} is the Kronecker delta. Typically, the anisotropic hyperfine terms are ~ 3 orders of magnitude smaller than the contact hyperfine terms for phosphorus dopants in silicon [25], and as such are ignored for normal operation. Since the contact hyperfine terms are typically on the order of $10-100$ MHz, anisotropic hyperfine terms are expected to be as high as ~ 100 kHz. However, if the contact hyperfine becomes close to twice the nuclear Zeeman splitting, or in other words $|\gamma_n|B_0 \approx A^{(i)}/2$, the anisotropic hyperfine couplings are likely to contribute to the nuclear spin dynamics. If we consider the terms of the Hamiltonian $\hat{H}_{\text{load}}(B_0)$, restricted to a subspace of the electron and only a single nuclear spin i, with only contact hyperfine and under the secular approximation [8], in the Zeeman basis $\{|{\uparrow\Uparrow}\rangle, |{\uparrow\Downarrow}\rangle, |{\downarrow\Uparrow}\rangle, |{\downarrow\Downarrow}\rangle\}$ we get:

$$\hat{H}_{\text{load}}(B_0) = \begin{bmatrix} \frac{\gamma_e+\gamma_n}{2}B_0 + \frac{A^{(i)}}{4} & 0 & 0 & 0 \\ 0 & \frac{\gamma_e-\gamma_n}{2}B_0 - \frac{A^{(i)}}{4} & 0 & 0 \\ 0 & 0 & \frac{-\gamma_e+\gamma_n}{2}B_0 - \frac{A^{(i)}}{4} & 0 \\ 0 & 0 & 0 & \frac{-\gamma_e-\gamma_n}{2}B_0 + \frac{A^{(i)}}{4} \end{bmatrix} \tag{100}$$

If $|\gamma_n|B_0 \approx \frac{A^{(i)}}{2}$, it is useful to rewrite this Hamiltonian as

$$\hat{H}_{\text{load}}(B_0) = \begin{bmatrix} \frac{\gamma_e}{2}B_0 + \frac{\Delta E_z^{(i)}}{2} & 0 & 0 & 0 \\ 0 & \frac{\gamma_e}{2}B_0 - \frac{\Delta E_z^{(i)}}{2} & 0 & 0 \\ 0 & 0 & \frac{-\gamma_e+\gamma_n}{2}B_0 - \frac{A^{(i)}}{4} & 0 \\ 0 & 0 & 0 & \frac{-\gamma_e-\gamma_n}{2}B_0 + \frac{A^{(i)}}{4} \end{bmatrix} \tag{101}$$

where $\Delta E_z^{(i)} = \gamma_n B_0 + A^{(i)}/2$ is the energy splitting between the nuclear spin states of spin i when the electron is $|{\uparrow}\rangle$. Note that γ_n is negative for phosphorus nuclei ($\gamma_n/2\pi = -17.24$ MHz/T), so $\Delta E_z^{(i)}$ can be close to zero for some combinations of B_0 and $A^{(i)}$, both of which are positive. To first order, adding AHF produces the following Hamiltonian in the electron $|{\uparrow}\rangle$ subspace:

$$\hat{H}'_{\text{load}}(B_0) \approx \begin{bmatrix} \frac{\Delta E_z^{(i)}}{2} + \frac{\left[A_{AHF}^{(i)}\right]_{z,z}}{4} & \frac{\left[A_{AHF}^{(i)}\right]_{z,x} - i\left[A_{AHF}^{(i)}\right]_{z,y}}{4} \\ \frac{\left[A_{AHF}^{(i)}\right]_{z,x} + i\left[A_{AHF}^{(i)}\right]_{z,y}}{4} & -\frac{\Delta E_z^{(i)}}{2} - \frac{\left[A_{AHF}^{(i)}\right]_{z,z}}{4} \end{bmatrix} + \frac{\gamma_e}{2}B_0 \tag{102}$$

The $\left[A_{AHF}^{(i)}\right]_{z,z}$ term only produces a shift in the energy splitting, and as such we can remove it by redefining ΔE_z:

$$\Delta E_z \rightarrow \Delta E_z^{(i)} - \frac{\left[A_{AHF}^{(i)}\right]_{z,z}}{2} \tag{103}$$

in which for $A_{AHF}^{(i)} \ll A^{(i)}$ (by a factor of around 1000 [25]), only a small correction to ΔE_z occurs. More importantly however is that the $\left[A_{AHF}^{(i)}\right]_{z,x}$ and $\left[A_{AHF}^{(i)}\right]_{z,y}$ terms represent off-diagonal elements which will mix any previously near-degenerate nuclear spin states, creating an anticrossing between $|{\uparrow\Uparrow}\rangle$ and $|{\uparrow\Downarrow}\rangle$. To approximate the strength of these off-diagonal mixing terms, $A_{AHF,M}^{(i)}$, we take their complex magnitude:

$$|A^{(i)}_{AHF,M}| \approx \sqrt{\left[A^{(i)}_{AHF}\right]^2_{z,x} + \left[A^{(i)}_{AHF}\right]^2_{z,y}} \tag{104}$$

Taking the complex magnitude is appropriate when considerng incoherent operations, ie. operations that do not depend on phase (eg. ionisation shock, chirped pulses). After neglecting the $\gamma_e B_0$ energy offset and absorbing $\left[A^{(i)}_{AHF}\right]_{z,z}$ into ΔE_z, we simplify the Hamiltonian in Eq. 102 to:

$$\hat{H}'_{\text{load}}(B_0) \approx \begin{bmatrix} \frac{\Delta E^{(i)}_z}{2} & \frac{A^{(i)}_{AHF,M}}{4} \\ \frac{A^{(i)}_{AHF,M}}{4} & -\frac{\Delta E^{(i)}_z}{2} \end{bmatrix} \tag{105}$$

From this Hamiltonian, we can see that the anisotropic hyperfine mixing due to $A^{(i)}_{AHF,M}$ is only relevant if $\left|\Delta E^{(i)}_z\right| \lesssim A^{(i)}_{AHF,M}$. In the following section, we calculate the effect of the Hamiltonian in Eq. 105 on electron tunnelling events. Note that the mixing of $|\uparrow\Uparrow\rangle$ and $|\uparrow\Downarrow\rangle$ also allows for magnetic driving of the normally magnetically forbidden transitions of $|\uparrow\Uparrow\rangle \leftrightarrow |\downarrow\Downarrow\rangle$ and $|\uparrow\Downarrow\rangle \leftrightarrow |\uparrow\Uparrow\rangle$. However, these transitions will only be driven if ESR pulses are close to resonant with them, and so for the simulations performed here we assume that for the majority of parameters simulated (ie. far from the AHF hotspot), that ESR will not be driven resonant with these transitions, and as such they can be neglected. This would not be the case if, for example, very wide chirps were used to invert the electron spin, or if one was specifically examining behaviour near the hotspot.

B. Nuclear spin SPAM errors resulting from AHF interaction during Ionisation Shock

As has been established in previous work on individual donor-dots in silicon [8], removing or adding the electron to either single or multidonor quantum dots has a small chance of flipping the nuclear spin(s), referred to as "ionisation shock". This is because, in the absence of anisotropic hyperfine as is generally assumed in previous experiments, the electron-(single) nuclear Hamiltonian before taking the secular approximation is:

$$\hat{H}'_{\text{load}}(B_0) = \begin{bmatrix} \frac{\gamma_e+\gamma_n}{2}B_0 + \frac{A^{(i)}}{4} & 0 & 0 & 0 \\ 0 & \frac{\gamma_e-\gamma_n}{2}B_0 - \frac{A^{(i)}}{4} & \frac{A^{(i)}}{2} & 0 \\ 0 & \frac{A^{(i)}}{2} & \frac{-\gamma_e+\gamma_n}{2}B_0 - \frac{A^{(i)}}{4} & 0 \\ 0 & 0 & 0 & \frac{-\gamma_e-\gamma_n}{2}B_0 + \frac{A^{(i)}}{4} \end{bmatrix} \tag{106}$$

which introduces a small mixing between states $|\uparrow\Downarrow\rangle$ and $|\downarrow\Uparrow\rangle$, leading to non-Zeeman eigenstates $|\widetilde{\uparrow\Downarrow}\rangle$ and $|\widetilde{\downarrow\Uparrow}\rangle$. As such, if an $\uparrow$ electron tunnels off from initial state $|\widetilde{\uparrow\Downarrow}\rangle$, or an electron $\downarrow$ tunnells onto the dot, there is a small chance that the nuclear spin will flip, with an error ϵ given by [28]:

$$\epsilon \approx \left(\frac{A}{2\gamma_e B_0}\right)^2 \tag{107}$$

where A is the contact hyperfine strength, γ_e is the electron gyromagnetic ratio, and B_0 is the constant magnetic field. The approximation here assumes $A, \gamma_n B_0 \ll \gamma_e B_0$ (where γ_n is the nuclear gyromagnetic ratio), with higher order corrections existing on order $O(\epsilon^2)$. Such corrections are typically not required for phosphorus atom spin systems with magnetic fields of $B_0 > 0.5$ T [28]. Note that each pathway requires the respective initial state (eg. initial state of $|\widetilde{\uparrow\Downarrow}\rangle$ for the pathway $|\widetilde{\uparrow\Downarrow}\rangle \rightarrow |0\Uparrow\rangle$), and will only have a chance of occurring for the corresponding electron tunnelling event (eg. electron $\uparrow$-out for the pathway $|\widetilde{\uparrow\Downarrow}\rangle \rightarrow |0\Uparrow\rangle$). Additional ionisation shock pathways can occur in multi-nuclear spin registers specifically [29], however these are only strong when two nuclei have similar hyperfines, and so this is not a dominant error source in this work.

In addition to ionisation caused by the contact hyperfine, AHF interactions can also cause ionisation shock by mixing nuclear states while the electron is present. Specifically, we consider mixing between $|\uparrow\Downarrow\rangle$ and $|\uparrow\Uparrow\rangle$, which is caused by the off-diagonal terms in Eq. 105. The eigenstates of Eq. 105 are:

$$\left\{ \frac{1}{\sqrt{a^2+1}} \begin{bmatrix} 1 \\ a \end{bmatrix}, \frac{1}{\sqrt{a^2+1}} \begin{bmatrix} -a \\ 1 \end{bmatrix} \right\} \tag{108}$$

with eigenenergies E_+ and E_- respectively, where

$$a = \frac{A^{(i)}_{AHF,M}}{2(2E_+ + \Delta E_z^{(i)})} \tag{109}$$

$$E_\pm = \pm\frac{1}{2}\sqrt{\left(\Delta E_z^{(i)}\right)^2 + \frac{1}{4}\left(A^{(i)}_{AHF,M}\right)^2} \tag{110}$$

This can be further simplified by defining R as the ratio between $\Delta E_z^{(i)}$ and $A^{(i)}_{AHF,M}$:

$$R \equiv \frac{\Delta E_z^{(i)}}{A^{(i)}_{AHF,M}} \tag{111}$$

$$a = \frac{1}{\sqrt{4R^2+1}+2R} \tag{112}$$

Note that for $\Delta E_z \geq 0$ (ie. $R \geq 0$), $0 \leq a \leq 1$; and for $\Delta E_z \leq 0$ (ie. $R \leq 0$), $a \geq 1$. In fact, $a(-R) = 1/a(R)$, which is a useful property as the errors induced by AHF interactions will be expressed in terms of the parameter a. Therefore, for $\Delta E_z \geq 0$, the E_+ eigenstate is mostly $|{\uparrow\Uparrow}\rangle$; and for $\Delta E_z \leq 0$, the E_+ eigenstate is mostly $|{\uparrow\Downarrow}\rangle$, according to Eq. 108. Hence, when comparing the eigenstates in Eq. 108 to the computational Zeeman basis, we map E_+ to $|{\uparrow\Uparrow}\rangle$ if $\Delta E_z \geq 0$, and E_+ to $|{\uparrow\Downarrow}\rangle$ if $\Delta E_z \geq 0$. The probability of ionisation shock occurring depends on the residual component of the eigenstate; ie. for E_+, the component of $|{\uparrow\Downarrow}\rangle$ if $\Delta E_z \geq 0$ and the component of $|{\uparrow\Uparrow}\rangle$ if $\Delta E_z \leq 0$. Using this property, we find the additional ionisation shock probabilities $P_{I.S.,AHF}$ that arise due to the presence of anisotropic hyperfine couplings:

$$P\left(|\widetilde{\uparrow\Uparrow}\rangle \leftrightarrow |0\Downarrow\rangle\right) = \left(\langle\widetilde{\uparrow\Uparrow}|0\Downarrow\rangle\right)^2 = P_{I.S.,AHF} \tag{113}$$

$$P\left(|\widetilde{\uparrow\Downarrow}\rangle \leftrightarrow |0\Uparrow\rangle\right) = \left(\langle\widetilde{\uparrow\Downarrow}|0\Uparrow\rangle\right)^2 = P_{I.S.,AHF} \tag{114}$$

$$P_{I.S.,AHF} = \begin{cases} \frac{a^2}{1+a^2} & \Delta E_z \geq 0 \\ \frac{1}{1+a^2} & \Delta E_z \leq 0 \end{cases} \tag{115}$$

Importantly, as $\Delta E_z \to 0$ (ie. $a \to 1$), the probability of ionisation shock $P_{I.S.,AHF} \to \frac{1}{2}$ per electron $\uparrow$ tunnel event. This is a significantly higher error rate than normal ionisation shock arising from the contact hyperfine, which is typically $10^{-8} - 10^{-4}$ per ionisation event. Such a large discrepancy arises due to the ability for AHF to fully mix the $|\widetilde{\uparrow\Uparrow}\rangle$ and $|\widetilde{\uparrow\Downarrow}\rangle$ states when $\Delta E_z = 0$. Anisotropic hyperfine ionisation shock only applies error rates close to $\frac{1}{2}$ in a small window where $\left|\Delta E_z^{(i)}\right| \lesssim A^{(i)}_{AHF,M}$. As has been previously predicted, $A^{(i)}_{AHF,M} \sim 100$ kHz, making this window very narrow. Despite this narrow window, the fact that other error sources are often $\lesssim 10^{-4}$ [8, 29], AHF-mediated ionisation shock is noticed over a wide range, as shown in the simulations of Fig. 4e of the main text.

If we instead limit ourselves to the electron-$\downarrow$ subspace, we get the following Hamiltonian:

$$\hat{H}'_{\text{load}}(B_0) \approx \begin{bmatrix} \frac{\gamma_n}{2}B_0 - \frac{A^{(i)}}{4} - \frac{\left[A^{(i)}_{AHF}\right]_{z,z}}{4} & \frac{\left[A^{(i)}_{AHF}\right]_{z,x} - i\left[A^{(i)}_{AHF}\right]_{z,y}}{4} \\ \frac{\left[A^{(i)}_{AHF}\right]_{z,x} + i\left[A^{(i)}_{AHF}\right]_{z,y}}{4} & -\frac{\gamma_n}{2} + \frac{A^{(i)}}{4} + \frac{\left[A^{(i)}_{AHF}\right]_{z,z}}{4} \end{bmatrix} - \frac{\gamma_e}{2}B_0 \tag{116}$$

which, after neglecting the global $\gamma_e B_0$ energy offset, can be written in the same form as Eq. 105:

$$\hat{H}'_{\text{load}}(B_0) \approx \begin{bmatrix} \frac{\Delta E_z^{(i)}}{2} & \frac{A^{(i)}_{AHF,M}}{4} \\ \frac{A^{(i)}_{AHF,M}}{4} & -\frac{\Delta E_z^{(i)}}{2} \end{bmatrix} \tag{117}$$

but where now

$$\Delta E_z^{(i)} = \gamma_n B_0 - \frac{A^{(i)}}{2} - \frac{\left[A_{AHF}^{(i)}\right]_{z,z}}{4} \tag{118}$$

Since γ_n is negative for phosphorus nuclear spins, ΔE_z will no longer be close to zero, and hence ionisation shock errors will not approach 50% as they do for electron-$|\uparrow\rangle$. However, using this modified expression for $\Delta E_z^{(i)}$, one can still model the residual nuclear spin errors incurred by AHF-induced ionisation shock for electron-$|\downarrow\rangle$ tunnel events. As such, Eq. 115 can be used to model both electron-$\uparrow$ and electron-$\downarrow$ errors during electron tunnelling, allowing us to quantify the errors from AHF-induced ionisation shock in Fig. 4e,f of the main text.

XIII. ADDITIONAL NUCLEAR SPIN SPAM ERRORS FROM AHF MEDIATED SPIN-0/2 TRANSITIONS

In addition to errors incurred during electron tunnelling, the AHF interaction can also induce errors during adiabatic electron inversion when operating very close to the hotspot. As explained in the main text, adiabatic electron spin resonance (ESR) is employed to perform non-demolition read out nuclear spin states. For any coupling $\hat{H}_C$ to drive transitions between two (undriven) eigenstates $|\psi_i\rangle$ and $|\psi_f\rangle$, the transition dipole matrix element

$$d_{i,f} \equiv \langle\psi_i| \hat{H}_C |\psi_f\rangle \tag{119}$$

must be non-zero, with its magnitude determining the strength/speed of the transition [53]. Without anisotropic hyperfine coupling, adiabatic ESR transitions implement a coupling proportional to any linear combination of σ_x and σ_y, ie. the electron Pauli x and y matrices. In this case, magnetic driving at ESR frequencies will only couple eigenstates which flip the electron spin but not nuclear spins as illustrated in Fig. 4a,c of the main text. Specifically, the total spin of the register is changed by exactly 1, and so these transitions are referred to as spin-1 transitions, a requirement of all magnetic drives as photons have spin 1. However, when anisotropic hyperfine coupling is introduced, the eigenstates involving electron $\uparrow$ become mixed, and so for the usual ESR transitions the strength is reduced:

$$d_{\widetilde{|\uparrow\Uparrow\rangle},|\downarrow\Uparrow\rangle} = \frac{1}{\sqrt{1+a^2}} d_0 \tag{120}$$

$$d_{\widetilde{|\uparrow\Downarrow\rangle},|\downarrow\Downarrow\rangle} = \frac{1}{\sqrt{1+a^2}} d_0 \tag{121}$$

with additional transitions becoming accessible:

$$d_{\widetilde{|\uparrow\Downarrow\rangle},|\downarrow\Uparrow\rangle} = \frac{a}{\sqrt{1+a^2}} d_0 \tag{122}$$

$$d_{\widetilde{|\uparrow\Uparrow\rangle},|\downarrow\Downarrow\rangle} = \frac{a}{\sqrt{1+a^2}} d_0 \tag{123}$$

where d_0 is the transition dipole moment for ESR in the absence of AHF. Here, for simplicity, $\Delta E_z \geq 0$ has been assumed - however, for $\Delta E_z \leq 0$, one can simply swap the terms $\frac{a}{\sqrt{1+a^2}}$ and $\frac{1}{\sqrt{1+a^2}}$. Equation 122 represents a transition which would normally be inaccessible to ESR, as the net spin changes by 0. Likewise, Eq. 123 represents a transition which normally cannot be driven by ESR as it is a spin-2 transition. That is, it changes the total spin of the register by 2. Both transitions are illustrated in Fig. 4b of the main text. Note that the energy splittings of the transitions $\widetilde{|\uparrow\Uparrow\rangle} \leftrightarrow |\downarrow\Uparrow\rangle$ and $\widetilde{|\uparrow\Downarrow\rangle} \leftrightarrow |\downarrow\Uparrow\rangle$ are roughly equal when operating close to the hotspot, differing by ΔE_z, and similarly $\widetilde{|\uparrow\Uparrow\rangle} \leftrightarrow |\downarrow\Uparrow\rangle$ and $\widetilde{|\uparrow\Downarrow\rangle} \leftrightarrow |\downarrow\Uparrow\rangle$ can be very close in frequency if ΔE_z is small. Importantly, for nuclear spin 2 in the device studied, both are within the chirp width of 10 MHz used to perform nuclear spin readout. As such, whenever any ESR transition is driven, these other pseudo spin-0/2 transitions will also be weakly driven, which now provides another mechanism for nuclear spin 2 to flip. In contrast, when investigating operation far from the hotspots (as per main text Fig. 4e-f), such transitions can be assumed to be sufficiently far outside the width of the frequency chirp used for adiabatic inversion, and so can be ignored.

During an adiabatic inversion pulse, the probability for an adiabatic transition to be taken (ie. to invert the system according to the transition) is given by the Landau-Zener formula:

$$P(\text{inv.}) = 1 - e^{-kd^2} \tag{124}$$

where k is a positive term that depends on the chirp rate, and d is proportional to the transition dipole moment of the transition. Experimentally, we observe that the chirped electron inversion probability is around $P(\text{inv.})_{d=d_0} = 98\%$ for nuclear spins 1 and 3. We use $d = d_0$ here because $\left|\Delta E_z^{(1)}\right| \gg A_{AHF,M}^{(1)}$ (ie. $a \to 0$), and similarly for spin 3. Hence, we can write the probabilities for a spin-1 and a spin-0/2 transition to be driven:

$$P(\text{inv.,spin-1}) = 1 - \left(1 - P(\text{inv.})_{d=d_0}\right)^{1/(1+a^2)} \tag{125}$$

$$P(\text{inv.,spin-0/2}) = 1 - \left(1 - P(\text{inv.})_{d=d_0}\right)^{a^2/(1+a^2)} \tag{126}$$

Whenever a spin-0/2 transition is inverted, nuclear spin 2 will be flipped. As such, Eq. 126 was used to generate Fig. 4g of the main text.

The probability in Eq. 126 assumes that the system starts in a state which is able to be inverted by the relevant spin-0/2 transition. This is not always true; eg. the spin-2 transition $|\downarrow\Downarrow\rangle \leftrightarrow |\widetilde{\uparrow\Uparrow}\rangle$ will not have any impact if the system is $|\downarrow\Uparrow\rangle$ or $|\widetilde{\uparrow\Downarrow}\rangle$. In particular, the direction of the frequency chirp (either increasing or decreasing frequency) dictates whether or not a spin-0/2 transition is likely to occur.

For example, consider a system which starts in the state $|\downarrow\Downarrow\rangle$, and a frequency chirp is applied to adiabatically invert the spin-1 transition $|\downarrow\Downarrow\rangle \leftrightarrow |\widetilde{\uparrow\Downarrow}\rangle$. For sufficiently low ΔE_z, the adiabatic frequency chirp will also cross the frequency of the spin-2 transition $|\downarrow\Downarrow\rangle \leftrightarrow |\widetilde{\uparrow\Uparrow}\rangle$. The resultant evolution of the state of the system is shown in Fig. S7 for the case where $\Delta E_z > 0$, with Fig. S7a showing the case where the chirp is applied with increasing frequency, and Fig. S7b showing the case where the chirp is applied with decreasing frequency. In Fig. S7a, the spin-1 transition $|\downarrow\Downarrow\rangle \leftrightarrow |\widetilde{\uparrow\Downarrow}\rangle$ is on-resonance first, meaning that the state $|\downarrow\Downarrow\rangle$ becomes $|\widetilde{\uparrow\Downarrow}\rangle$ with the spin-2 transition $|\downarrow\Downarrow\rangle \leftrightarrow |\widetilde{\uparrow\Uparrow}\rangle$ having no effect. In contrast, in Fig. S7b, the spin-2 transition is resonant first allowing for the system to evolve from $|\downarrow\Downarrow\rangle$ to $|\widetilde{\uparrow\Uparrow}\rangle$ and therefore allowing the possibility of error (ending in state $|\widetilde{\uparrow\Uparrow}\rangle$), and only in the case of no error does the system take the usual spin-1 transition to $|\widetilde{\uparrow\Downarrow}\rangle$. In the case that $\Delta E_z < 0$, the frequencies become resonant in the opposite order, meaning that a decreasing chirp would have a chance to have a spin-2 error, but an increasing chirp would not. Generally, the probability of a spin-0 error is:

$$P(\text{inv.,spin-0}) = \begin{cases} 1 - \left(1 - P(\text{inv.})_{d=d_0}\right)^{a^2/(1+a^2)} & \text{if } \ \Delta E_z > 0, \Delta f > 0 \\ 0 & \text{if } \ \Delta E_z > 0, \Delta f < 0 \\ 0 & \text{if } \ \Delta E_z < 0, \Delta f > 0 \\ 1 - \left(1 - P(\text{inv.})_{d=d_0}\right)^{a^2/(1+a^2)} & \text{if } \ \Delta E_z < 0, \Delta f < 0 \end{cases} \tag{127}$$

where $\Delta f > 0$ indicates an increasing chirp, and $\Delta f < 0$ indicates a decreasing chirp. Likewise, the probability of a spin-2 error is:

$$P(\text{inv.,spin-0}) = \begin{cases} 0 & \text{if } \ \Delta E_z > 0, \Delta f > 0 \\ 1 - \left(1 - P(\text{inv.})_{d=d_0}\right)^{a^2/(1+a^2)} & \text{if } \ \Delta E_z > 0, \Delta f < 0 \\ 1 - \left(1 - P(\text{inv.})_{d=d_0}\right)^{a^2/(1+a^2)} & \text{if } \ \Delta E_z < 0, \Delta f > 0 \\ 0 & \text{if } \ \Delta E_z < 0, \Delta f < 0 \end{cases} \tag{128}$$

As such, it is important to consider the sign of ΔE_z and the direction of frequency chirp when calculating spin-0/2 error rates.

XIV. FITTING OF ANISOTROPIC HYPERFINE ERRORS

In Figure 4d of the main text, non-demolition readouts of nuclear spins 1 and 2 are performed to establish the strength of the anisotropic hyperfine mixing term $A_{AHF,M}^{(2)}$ and characterise the errors it incurs on nuclear spin 2.

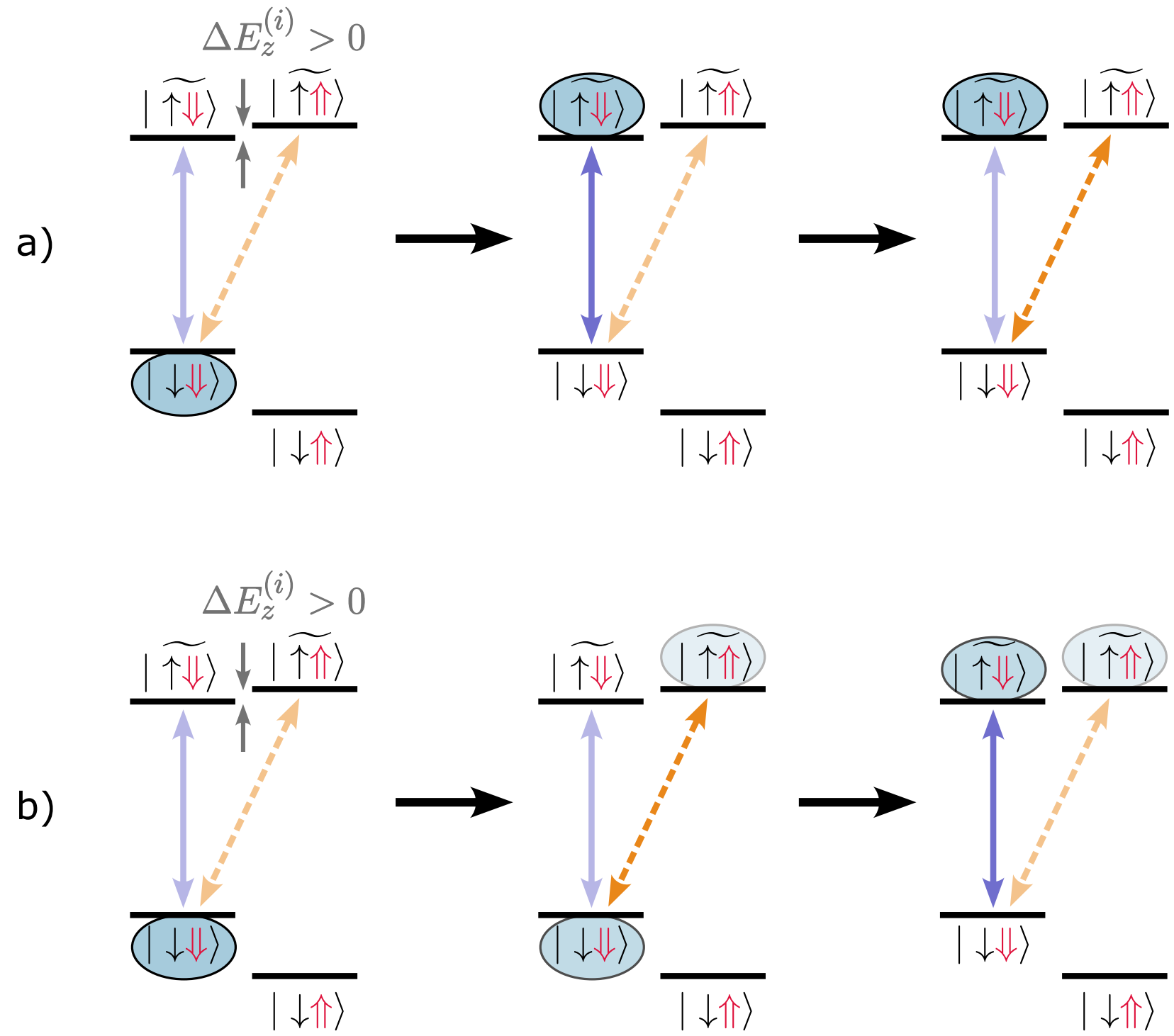

FIG. S7. **Impact of chirp direction on spin-0/2 errors.** The evolution of an electron-nuclear system starting in state $|\downarrow\Downarrow\rangle$, with a small-but-positive energy splitting $\Delta E_z > 0$. A frequency chirp is applied to adiabatically invert the spin-1 transition $|\downarrow\Downarrow\rangle \leftrightarrow |\widetilde{\uparrow\Downarrow}\rangle$ (blue), but this frequency chirp also crosses the resonant frequency of the spin-2 transition $|\downarrow\Downarrow\rangle \leftrightarrow |\widetilde{\uparrow\Uparrow}\rangle$ (orange, dashed). We illustrate two cases: (a) when the frequency chirp is applied with increasing frequency, and (b) when the frequency chirp is applied with decreasing frequency. In (a), the system is first inverted by the spin-1 transition (blue), avoiding the possibility of any spin-2 errors, whereas in (b) the spin-2 transition (orange) is resonant first, allowing for spin-2 errors. The circled state(s) represents the potential state(s) of the system at each step, with the relative intensity of the circle illustrating the probability of each state being occupied, and the highlighted transitions represent which transition is resonant at each step.

These measurements are repeated for different magnetic fields B_0, which allows us to vary the ratio R of ΔE_z and $A_{AHF,M}$ as defined by Eq. 111 (reproduced here for convenience):

$$R \equiv \frac{\Delta E_z^{(i)}}{A_{AHF,M}^{(i)}} \tag{129}$$

We can therefore vary the errors caused by the AHF coupling which affect nuclear spin 2. By finding the ratio $|R|$ separately at each magnetic field B_0, we can then fit R as a function of B_0 to find $A_{AHF,M}^{(2)}$ and $A^{(2)}$ using the process outlined in the main text.

For the measurements used to produce Fig. 4d of the main text, readout is performed in the order $\hat{I}_z^{(2)} = \Downarrow$, $\hat{I}_z^{(2)} = \Uparrow$, $\hat{I}_z^{(1)} = \Downarrow$, $\hat{I}_z^{(1)} = \Uparrow$, which is then repeated $N = 49$ times. Nuclear spin 1 is used as an experimental control, as it has significantly lower errors compared to nuclear spin 2 as per Fig. 2h-i. It can therefore be used to characterise errors related to electron readout, without confusing electron readout errors with those arising from the AHF.

A. Markov Modelling of Time Dynamics of the Multi-Nuclear Spin Register

To model the nuclear spin readout error rate and extract an estimate for the AHF strength via the parameter $|R|$ (as per Fig. 4d of the main text), the following parameters are considered for the fitting algorithm:

- The parameter a, given by Eq. 112 as $a = 1/(\sqrt{4R^2+1} + 2R)$, for nuclear spin 2.

- The probability for ESR to invert the electron in the absence of AHF coupling, $P(\text{inv.})_{d=d_0}$, as per Eq. 126. This is only used when calculating spin-0/2 errors.
- The probabilities for nuclear spins 1 or 2 to be $\Downarrow$ at the start of the nuclear non-demolition measurement, $P_0(\hat{I}_z^{(1)} = \Downarrow)$ and $P_0(\hat{I}_z^{(2)} = \Downarrow)$ respectively.
- The probability that a $|\uparrow\rangle$ is correctly measured (ie. a "blip" is seen) when the driven ESR peak is resonant, $P_b(\uparrow)$. This is a single parameter which absorbs all fidelities relating to electron initialisation, electron inversion (other than spin-0/2 errors), electron spin-to-charge and charge readout processes. For example, if nuclear spin 2 is $\Downarrow$, a single non-demolition readout shot of $\hat{I}_z^{(2)} = \Downarrow$ will register a blip with probability $P_m(\uparrow)$.
- The probability that a $|\downarrow\rangle$ is incorrectly measured (ie. a "blip" is seen) when the driven ESR peak is not resonant, $P_b(\downarrow)$, similar to $P_m(\uparrow)$ but when the nuclear spin is in a state that should not produce a blip.
- The probability that electron readout will be failing at the start of the non-demolition readout, $P(\text{fail})$. Such readout failure has been seen to occur for intervals of a few seconds, eg. as is seen in Fig. 2b-d of the main text at times 110-115s. During these periods, blips registered during nuclear non-demolition readout will be independent of the nuclear spin state.
- The probability, per non-demolition readout shot, that readout currently failing readout will stop failing, $P(\text{stop fail})$. Note that this is taken per individual shot (eg. a single measurement of $\hat{I}_z^{(2)} = \Downarrow$), so that in total there are $4N$ chances for the readout to stop failing for the entire experimental sequence, accounting for measuring both spins 1 and 2 for states $\Downarrow$ and $\Uparrow$.
- The probability that during a readout failure, electron readout produces a blip, $P_b(\text{fail})$.

In addition to this choice of parameters, we make the following assumptions:

- Nuclear spin 1 has negligible AHF error when compared to nuclear spin 2, so simulations of nuclear spin 1 can assume $a = 0$. This is because $\Delta E_z = 74.8126(1)$ MHz, which will greatly suppress any AHF effects for nuclear spin 1.
- When a series of 4 ESR peaks is driven to take a single non-demolition readout shot, we assume that the chance of two errors occurring is small. That is, we neglect situations where driving one ESR peak causes a spin-0/2 error, only for a later peak to *also* cause a spin-0/2 error, causing no error overall. This is assumption is equivalent to our choice of ESR chirp direction, which always ensures no spin-0 errors occur (only spin-2 errors), and spin-2 errors always act in the direction $\Downarrow \rightarrow \Uparrow$ (see Fig. S7). Hence, no secondary errors will occur in the measurements taken here.
- Closely related to the previous assumption, we assume that ESR will cause the electron to flip (subject to components of $P_b(\uparrow)$) if the nuclear spin starts in the correct state for a single shot of nuclear non-demolition readout, regardless of whether a spin-0/2 error occurs. That is, we neglect where driving one ESR peak causes a spin-0/2 error, only for a later ESR peak to invert the electron back to $|\downarrow\rangle$. This will never happen when reading out nuclear spin 2 (since when a spin-0/2 error happens, the subsequent ESR peaks are no longer on resonance), and we do not record the probability for blips when measuring nuclear spin 1 when modelling errors on nuclear spin 2, so that case is also not a problem. Instead, the only error source which this assumption neglects is a lack of ionisation shock, but only after a spin-0/2 error occurs when measuring nuclear spin 1, which is again second-order in the AHF error. Hence, provided the measured AHF error is small, these terms can be ignored.

Under this choice of parameters and assumptions, we simulate the accumulation of errors using a Markov model. Firstly, we construct nuclear spin error rates for a single shot of nuclear spin readout, assuming that no spin-0 errors occur (since we choose our chirp direction to avoid EDSR errors), so only spin-2 errors will occur, which only affect the nuclear spin 2 if it starts $|\Downarrow\rangle$. Under this assumption, we get the following:

$$\begin{aligned} \epsilon_{\Downarrow} &= P_{IS,AHF}\left[1 - P(\text{inv.,spin-0/2})\right] + \left[1 - P_{IS,AHF}\right] P(\text{inv.,spin-0/2}) && (130) \\ &= \frac{1}{1+a^2} + \frac{a^2-1}{a^2+1}\left(1 - P(\text{inv.})_{d=d_0}\right)^{a^2/(1+a^2)} && (131) \\ \epsilon_{\Uparrow} &= P_{IS,AHF} && (132) \\ &= \frac{a^2}{1+a^2} && (133) \end{aligned}$$

where $\epsilon_{\Downarrow}$ is the probability for the nuclear spin to flip during a single non-demolition measurement shot if it starts $\Downarrow$, provided it is on-resonance with ESR. Similarly, $\epsilon_{\Uparrow}$ is the same case but with the nuclear spin starting $\Uparrow$. For example, if errors on nuclear spin 2 are being considered, and it starts $\Downarrow$, there will be probability $\epsilon_{\Downarrow}$ for a nuclear spin flip during readout of $\hat{I}_z^{(2)} = \Downarrow$, and then if it is still $\Downarrow$, no chance for it to flip during readout of $\hat{I}_z^{(2)} = \Uparrow$, and

exactly one more chance for it to flip during readouts of $\hat{I}_z^{(1)} =\Downarrow$ and $\hat{I}_z^{(1)} =\Uparrow$, because reading out this pair will cover all ESR peaks exactly once.

The algorithm used will repeatedly solve the subproblem

$$P(n, m, n_{b,\downarrow}, n_{b,\uparrow}, s, f) \tag{134}$$

where all parameters are integers, defined by:

- $n \in [0, N]$ is the number of non-demolition readout cycles completed so far (where a cycle involves measuring all 4 of $\hat{I}_z^{(2)} =\Downarrow$, $\hat{I}_z^{(2)} =\Uparrow$, $\hat{I}_z^{(1)} =\Downarrow$, $\hat{I}_z^{(1)} =\Uparrow$).
- $m \in [0, 2]$ is the stage of the non-demolition readout cycle that has been completed ($m = 0 \implies \hat{I}_z^{(2)} =\Downarrow$ completed, $m = 1 \implies \hat{I}_z^{(2)} =\Uparrow$ completed, and $m = 1 \implies \hat{I}_z^{(1)} =\Downarrow$ and $\hat{I}_z^{(1)} =\Uparrow$ completed).
- $n_{b,\downarrow} \in [0, N]$ is the number of blips (ie. readouts registering electron-$|\uparrow\rangle$) measured during readouts of $\hat{I}_z^{(2)} =\Downarrow$ so far.
- $n_{b,\uparrow} \in [0, N]$ is the number of blips measured during readouts of $\hat{I}_z^{(2)} =\Uparrow$ so far.
- $s \in [0, 1]$ is the Markov-modelled true nuclear spin state of spin 2 ($s = 0 \implies \Downarrow$, $s = 1 \implies \Uparrow$).
- $f \in [0, 1]$ represents whether electron readout is in a failing state ($f = 0$ indicates no readout failure, $f = 1$ indicates readout failure)

and finally, the subproblem $P(n, m, n_{b,\downarrow}, n_{b,\uparrow}, s, f)$ represents the probability of the system being in the state (s, f) after measauring $(n_{b,\downarrow}, n_{b,\uparrow})$ blips at time (n, m). The initial state when the simulation starts with the nuclear spin in state s is given by:

$$P(n = 0, m, n_{b,\downarrow} = 0, n_{b,\uparrow} = 0, s, f = 0) = 1 - P(\text{fail}) \qquad \forall m \tag{135}$$

$$P(n = 0, m, n_{b,\downarrow} = 0, n_{b,\uparrow} = 0, s, f = 1) = P(\text{fail}) \qquad \forall m \tag{136}$$

with all other entries starting at 0. The subproblems are then solved in increasing order of m, and then increasing order of n (ie. in chronological order), with probabilities being updated at the next time step according to the aforementioned parameters, along with the error rates calculated in Eqns. 131 and 133. For example, the first step of the algorithm makes the following updates (modelling the first readout of $\hat{I}_z^{(2)} =\Downarrow$):

$$P(1, 0, 0, 0, 1, 0) += \big(1 - P_b(\downarrow)\big)\Big[P(0, 2, 0, 0, 1, 0) + P(\text{stop fail}) \cdot P(0, 2, 0, 0, 1, 1)\Big] \tag{137}$$

$$P(1, 0, 0, 0, 0, 0) += \big(1 - \epsilon_{\Downarrow}\big)\big(1 - P_b(\uparrow)\big)\Big[P(0, 2, 0, 0, 0, 0) + P(\text{stop fail}) \cdot P(0, 2, 0, 0, 0, 1)\Big] \tag{138}$$

$$P(1, 0, 1, 0, 1, 0) += P_b(\downarrow)\Big[P(0, 2, 0, 0, 1, 0) + P(\text{stop fail}) \cdot P(0, 2, 0, 0, 1, 1)\Big] \tag{139}$$

$$P(1, 0, 1, 0, 0, 0) += \big(1 - \epsilon_{\Downarrow}\big)P_b(\uparrow)\Big[P(0, 2, 0, 0, 0, 0) + P(\text{stop fail}) \cdot P(0, 2, 0, 0, 0, 1)\Big] \tag{140}$$

$$P(1, 0, 0, 0, 1, 0) += \epsilon_{\Downarrow}\big(1 - P_b(\uparrow)\big)\Big[P(0, 2, 0, 0, 0, 0) + P(\text{stop fail}) \cdot P(0, 2, 0, 0, 0, 1)\Big] \tag{141}$$

$$P(1, 0, 1, 0, 1, 0) += \epsilon_{\Downarrow}P_b(\uparrow)\Big[P(0, 2, 0, 0, 0, 0) + P(\text{stop fail}) \cdot P(0, 2, 0, 0, 0, 1)\Big] \tag{142}$$

where $+=$ is defined such that $x += y$ indicates the increment $x \to x + y$. In addition to the above updates, similar updates are made to all the failing cases (ie. $P(\ldots, 1)$) with all the same terms, except no $P(\ldots, 0)$ terms are considered, $P(\text{stop fail})$ is replaced with $1 - P(\text{stop fail})$, and all blip probabilities (ie. $P_b(\downarrow), P_b(\uparrow)$) are replaced with the failure blip probability $P_b(\text{fail})$.

This update procedure continues until all subproblems have been solved, which is run once starting in the state $s =\Downarrow$ and $s =\Uparrow$. Finally, these subproblems are combined in a weighted average according to the initial state probability $P_0(\hat{I}_z^{(2)} =\Downarrow)$. In aggregate, these simulations provide the probability distribution for numbers of observed blips during the full non-demolition readout for a given set of parameters $a, P(\text{inv.})_{d=d_0}$, $P_0(\hat{I}_z^{(2)} =\Downarrow), P_b(\uparrow), P_b(\downarrow), P(\text{fail}), P(\text{stop fail})$ and $P_b(\text{fail})$.

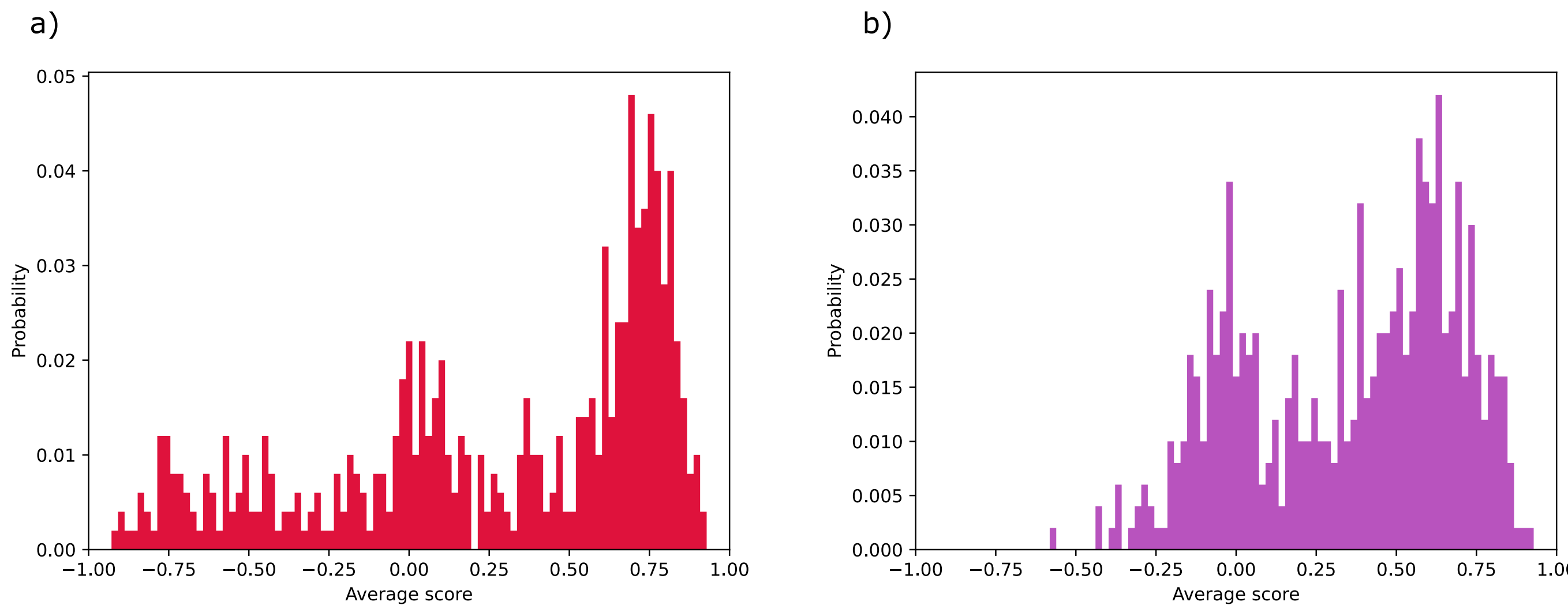

FIG. S8. **Example histograms of nuclear spin readout scores after doing non-demolition readout for $N = 49$ repetitions.** Histograms are given for nuclear spin 1 (a) and nuclear spin 2 (b), as per the method outlined in Supporting Information XIV, as measured for a magnetic field of $B_0 = 1.38$ T.

B. Fitting the anisotropic hyperfine error rates

Using the theoretical simulation model established in the previous section, we can now fit the experimental data observed at different magnetic fields B_0. At each magnetic field, nuclear non-demolition readout was performed with $N = 49$ repetitions, with each repetition recording a single shot of $\hat{I}_z^{(2)} = \Downarrow$, $\hat{I}_z^{(2)} = \Uparrow$, $\hat{I}_z^{(1)} = \Downarrow$, and $\hat{I}_z^{(1)} = \Uparrow$. Then, we separately analyse the readout result of nuclear spin 1 and nuclear spin 2. For each nuclear spin i, we assign a score of $+1$ to the case where a single pair of non-demolition readouts $\hat{I}_z^{(i)} = \Downarrow$ and $\hat{I}_z^{(i)} = \Uparrow$ produces a blip when measuring $\Uparrow$ but no blip when measuring $\Downarrow$. Similarly, we assign a score of -1 when measuring $\Downarrow$ produces a blip but $\Uparrow$ doesn't, and otherwise (if both or neither produce blips) we assign a score of 0. After multiple non-demolition readouts, an average score closer to $+1$ indicates nuclear spin i has been measured to be $\Uparrow$, and similarly an average score close to -1 indicates a measurement of $\Downarrow$. By repeating the non-demolition readout sequence many times, we can produce a histogram of the different average scores obtained using this method. An example of such histograms for nuclear spins 1 and 2 are shown in Fig. S8.

Instead of examining histograms such as those in Fig. S8, it is easier to examine nuclear spin error accumulation by considering how these histograms vary as a function of N, as each additional repetition has the chance to introduce error to nuclear spin 2, making errors accumulate with increasing N. Instead of retaking data for various values of N, we instead construct histograms from the first n ($3 \leq n \leq N$) non-demolition readouts performed in the $N = 49$ repetition measurement. These histograms are plotted as a function of N in Fig. S9 for $B_0 = 1.31$ T (close to the hotspot for nuclear spin 2), and $B_0 = 1.38$ T (far from the hotspot). This figure contains a strong vertical line at an average score of 0, which corresponds to non-demolition readouts where the electron readout was failing. Despite the presence of this central line, peaks far from 0 can also be seen. In these non-0 peaks, we can see that far from the hotspot (Fig. S9c-d), nuclear spin 1 and nuclear spin 2 have similar histograms as a function of n. In contrast, close to the hotspot (Fig. S9a-b), the average score of nuclear spin 2 rapidly approaches a value close to 0, indicating large error accumulation on nuclear spin 2, while nuclear spin 1 remains at a low error rate (with no significant bunching towards 0).

Data similar to Fig. S9 was taken at all B_0 values sampled in Fig. 4d of the main text. These were then fit, using the Markov model outlined above, to obtain a value for a at each magnetic field. Using the definition of a given by Eq. 112 (reproduced here for convenience):

$$a = \frac{1}{\sqrt{4R^2 + 1} + 2R} \tag{143}$$

values for $|R|$ were then calculated for each magnetic field, as shown in Fig. 4d of the main text. Example fits to the data shown in Fig. S9 are shown in Fig. S10.

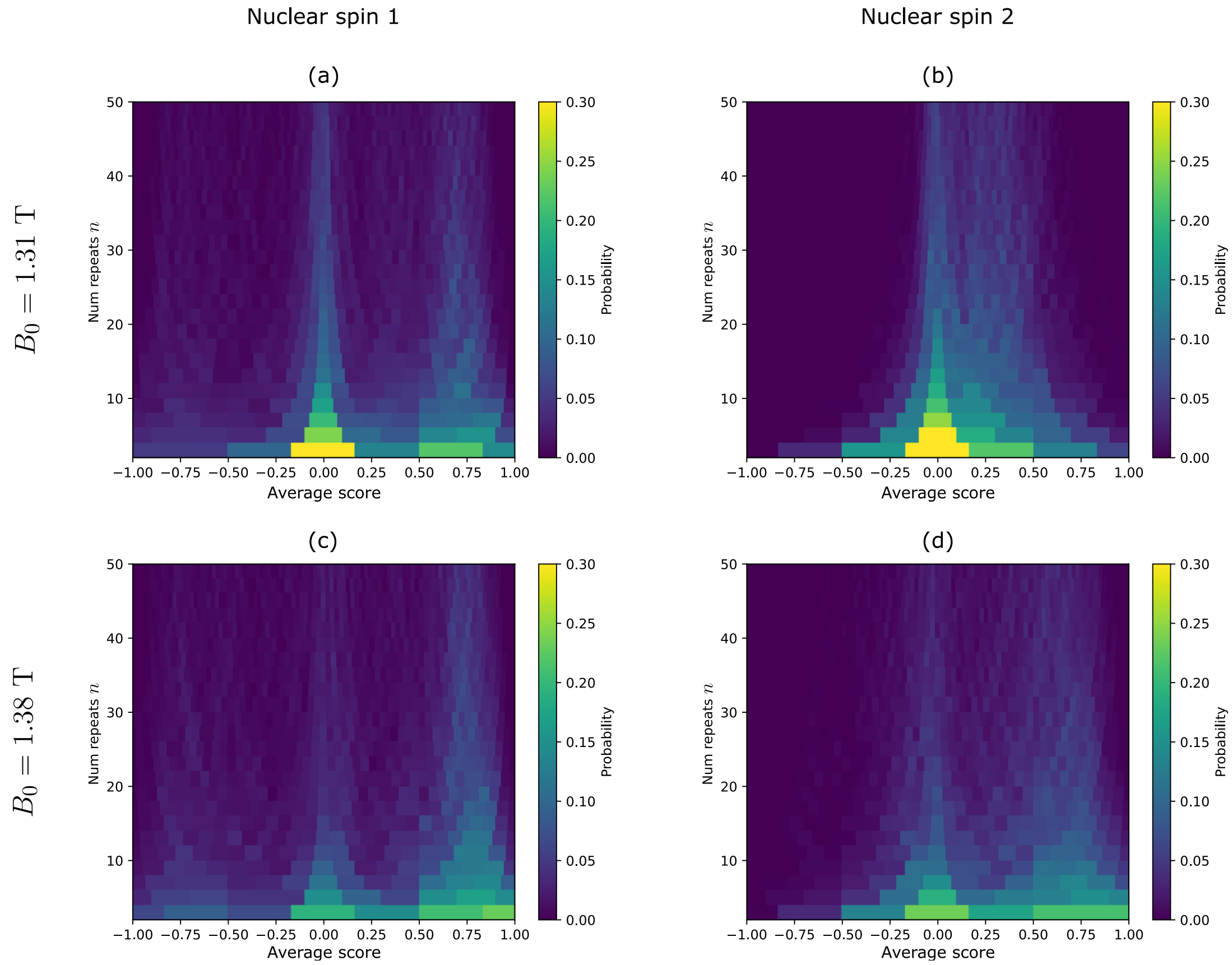

FIG. S9. **Example histograms of average nuclear spin readout scores for repetitions $\mathbf{n} \in [\mathbf{3}, \mathbf{49}]$.** Histograms are given for nuclear spin 1 (a,c) and nuclear spin 2 (b,d), as per the method outlined in Supporting Information XIV. (a) and (b) are taken at a magnetic field of $B_0 = 1.31$ T, and likewise (c) and (d) are taken at $B_0 = 1.38$ T. Histograms similar to these are used to fit the error for nuclear spin 2 due to the AHF interaction, according to the Markov method outlined in Supporting Information XIV to produce Fig. 4d of the main text.

Note that a single outlier was removed, corresponding to the measurement at $B_0 = 1.305$ T, which gave a ratio of $|R| = 1.874$. This outlier was likely caused by a failure of the above fitting procedure, or potentially caused by a temporary shift in the contact hyperfine of nuclear spin 2 (bringing it further from the hotspot).

XV. ESTIMATION OF HOTSPOT WIDTHS FOR NUCLEAR SPIN 2

To determine the width of the hotspot of errors induced for ionisation shock, we examine the error hotspot simulated in Fig. 4e and determine the range of magnetic fields within which errors are $> 1\%$ or $> 0.1\%$. A zoom near the error hotspot is shown in Fig. S11, which indicates that the width of the hotspot is ~ 300 mT for $> 1\%$ error, and ~ 1 T for $> 0.1\%$ error.

Since the amount of error caused by the anisotropic hyperfine depends on the energy splitting $\Delta E_z^{(i)}$ between $|\uparrow\Downarrow\rangle$ and $|\uparrow\Uparrow\rangle$:

$$\Delta E_z^{(i)} \equiv \gamma_n B_0 + A^{(i)}/2 \tag{144}$$

as per Eq. 11 of the main text, we can calculate the hotspot width in terms of contact hyperfine strength $A^{(i)}$. Specifically, to create the same range of $\Delta E_z^{(i)}$ values as variation in B_0, $A^{(i)}$ must vary by:

$$\Delta A^{(i)} = 2\gamma_n \Delta B_0 \tag{145}$$

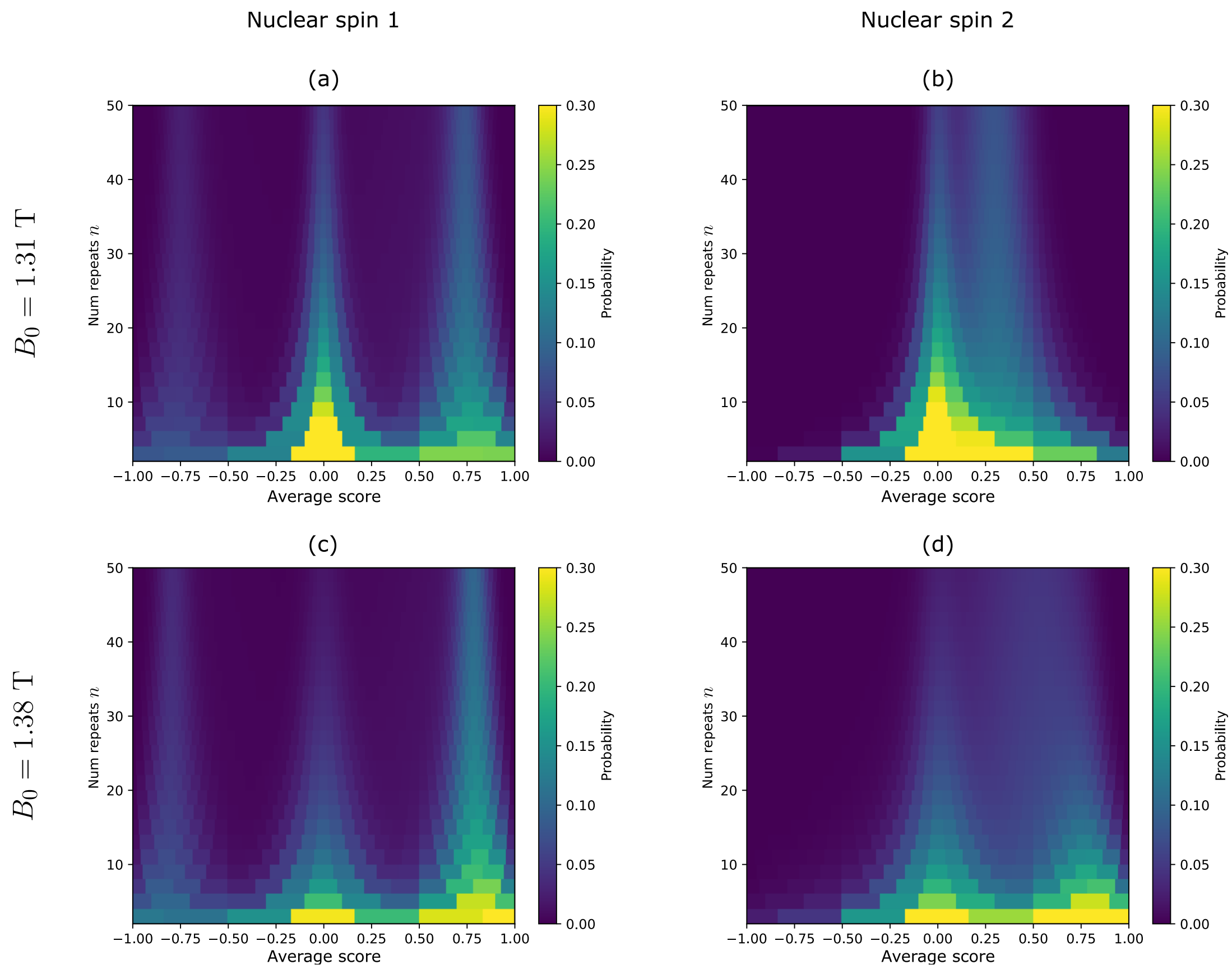

FIG. S10. **Example numerical fits to the data shown in Figure S9.** The plots given in Fig. S9 were fit using the Markov model outlined in Supplementary Information XIV. Subfigures correspond to those in Fig. S9, meaning (a,c) are for nuclear spin 1, (b,d) are for nuclear spin 2, (a,b) are for $B_0 = 1.31$ T, and (c,d) are for $B_0 = 1.38$ T. Such fits are used to find the error for nuclear spin 2 due to the AHF interaction to produce Fig. 4d of the main text.

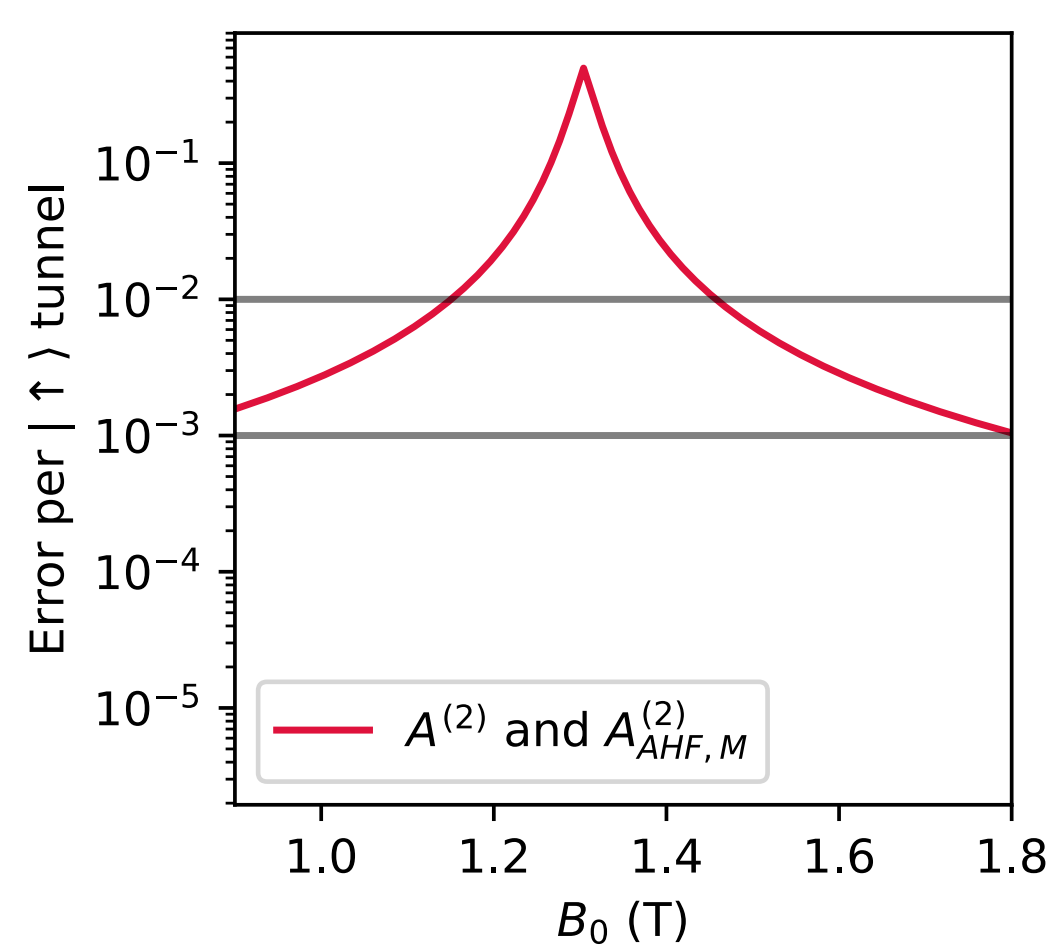

FIG. S11. **Zoom near the error hotspot in Fig. 4e of the main text.** The same procedure used to generate Fig. 4e is used here, but only for magnetic fields near the error hotspot. Horizontal grey lines indicate the 1% and 0.1% error levels, which are used to determine the width of the error hotspot in magnetic field. This was used to estimate the width of the hotspot in the main text.

where $\Delta A^{(i)}$ is the hotspot width in contact hyperfine, and ΔB_0 is the hotspot width in magnetic field. Using magnetic hotspot widths of ~ 300 mT and ~ 1 T, we get contact hyperfine hotspot widths of ~ 10 MHz and ~ 33 MHz respectively, as is used in the main text.
